# Supplementary material for: Capacity and quality of maternal and child health services delivery at the subnational primary healthcare level in relation to intermediate health outputs: a cross-sectional study of 12 low-income and middle-income countries
Source: BMJ Open. 2023 Jan 31;13(1):e065223. doi: 10.1136/bmjopen-2022-065223 (PMC9890757; doi:10.1136/bmjopen-2022-065223)
Supplement: Supplementary data [file bmjopen-2022-065223supp001.pdf]

Supplementary Methods

Supplementary Table 1: Definition of capacity, quality and output indicators utilized in the analysis

Domain	Subdomain	Indicator	Definition	Data Source
**Capacity**	Facility Infrastructure	SRI for basic amenities and infection prevention	Service readiness index for basic amenities and infection prevention	SPA
	Recent Supervision	Supervision within the past 6 months	Proportion of providers within a facility with supervision in past 6 months	SPA
	Supervisor Feedback	Supervisor provided verbal or written feedback	Proportion of providers within a facility provided verbal or written supervision feedback	SPA
	Outreach Services	Facility provides outreach services	Facility provides sick child, growth monitoring or immunization outreach service	SPA
	Not charging PHC Fees	Facility does not charge PHC fees	Facility does not charge fees for PHC services (fees for a specific PHC service or a fixed fee that covers all services that a client receives)	SPA
**Quality**	Waiting Time < 60 minutes	Wait time less than one hour	Proportion of observed visits with wait times less than one hour	SPA
	Provider Availability	Proportion of visits over 10 minutes	The proportion of ANC, FP, LD and sick child visits that last for more than 10 minutes.	SPA
	Provider Competence	Antenatal care quality score based on WHO guidelines	Average quality score for observed ANC visits based on WHO ANC guidelines	SPA
		Family planning quality score based on WHO guidelines	Average quality score for observed family visits based on WHO family planning guidelines	
		Sick child quality score based on IMCI guidelines	Average quality score for observed sick child visits based on WHO Integrated Management of Childhood Illness (IMCI) guidelines	
	Person Centeredness	Proportion of caregivers who were told the sick child diagnosis	Proportion of observed sick child visits where the health worker told the child's caretaker what illness(es) the child has	SPA
	Safety	Proportion of rooms with all infection control items	Proportion of rooms (FP, sick child, ANC and NCD) where all infection control tracer items are present	SPA
		Adequate waste disposal	Average score (out of 3) on adherence to standards for disposing of medical and hazardous waste, sharps, and having guidelines for waste disposal in place.	
	Comprehensiveness	Proportion of 3 NCD services offering diagnosis and treatment (diabetes, CRD, CVD)	Proportion of non-combinable disease services provided and for which guidelines are available (diabetes, chronic respiratory disease, and chronic cardiovascular disease) across all facilities	SPA
		Proportion of 3 primary ID services provided (STI, TB, HIV)	Proportion of infectious diseases services provided and for which guidelines are available for sexually transmitted infections, tuberculosis, and HIV across all facilities.	SPA
		Average availability of 5 tracer RMNCH services (SC, Vacc, FP, ANC, PMTCT)	Proportion of maternal and child health services provided and for which guidelines are available (sick child, vaccination, family planning, antenatal care, and prevention of mother-to-child transmission of HIV) across all facilities.	SPA
**Outputs**	Maternal	Antenatal care coverage - at least four visits (%)	Antenatal care coverage (4+) visits is the percent of women with a live birth who received antenatal care (ANC) 4 or more times.	DHS
		Demand for family planning satisfied with modern methods (%)	Proportion of married or in-union women of reproductive age (aged 15-49 years) who are married or in a union and have their need for family planning satisfied with modern methods.	DHS
		Perceived barriers due to treatment costs	Percentage of women who report specific problems in accessing health care when they are sick due to issues related to getting money for treatment	DHS
		Perceived barriers due to distance	Percentage of women who report specific problems in accessing health care when they are sick due to distance that they have to travel for treatment	DHS
	Child	Coverage of DTP3 immunization	Diphtheria-tetanus-pertussis (DTP) coverage measures the percent of one-year-olds who have received three doses of the combined diphtheria, tetanus toxoid and pertussis vaccine in a given year.	DHS
		DPT Dropout rate	Percent of children who do not receive three doses of DTP after receiving an initial dose. Calculated as (DPT1-DPT3/DPT1)	DHS
		Children aged < 5 years with pneumonia symptoms taken to a healthcare provider (%)	Percentage of children under 5 years of age with suspected pneumonia (cough and difficulty breathing NOT due to a problem in the chest and a blocked nose) in the two weeks preceding the survey taken to an appropriate health facility or provider.	DHS
		Children aged < 5 years with diarrhea receiving oral rehydration salts	The percent of children with diarrhea, a leading cause of death in children under five, who received appropriate treatment with oral rehydration solution.	DHS

Supplementary Figure 1: Linkage of SPA and DHS datasets

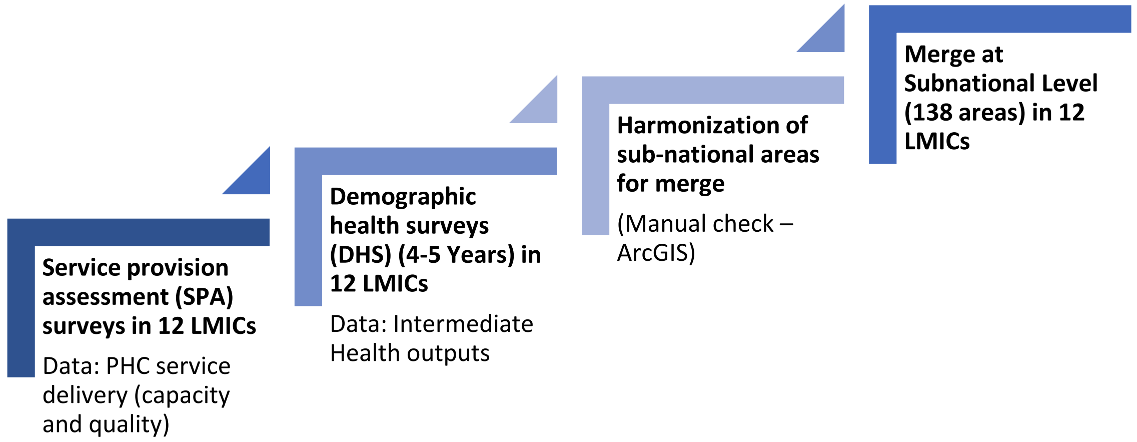

12 Countries: Afghanistan, Bangladesh, DRC, Haiti, Kenya, Malawi, Namibia, Nepal, Rwanda, Senegal, Tanzania, Uganda)

Supplementary Results

Supplementary Table 2: Capacity of PHC service delivery at the subnational level in the studied countries

Country	Division	Facility Infrastructure	Recent Supervision	Supervisor Feedback	Outreach Services	Not Charging PHC Fees	Average Capacity score	Range across capacity sub-domains*
Afghanistan	Balkh	83%	85%	63%	7%	56%	59%	78%
	Herat	85%	59%	29%	4%	71%	50%	81%
	Kabul	86%	63%	54%	31%	36%	54%	55%
	Kandahar	82%	99%	73%	29%	45%	66%	70%
	Kunduz	76%	91%	76%	0%	53%	59%	91%
	Nangarhar	75%	70%	63%	0%	45%	51%	75%
	Paktya	74%	70%	44%	25%	43%	51%	49%
Bangladesh	Barisal	49%	95%	93%	14%	67%	64%	81%
	Chittagong	51%	91%	85%	20%	80%	65%	71%
	Dhaka	52%	92%	89%	26%	54%	63%	66%
	Khulna	54%	97%	92%	21%	54%	64%	76%
	Mymensingh	46%	99%	96%	14%	45%	60%	85%
	Rajshahi	55%	96%	94%	19%	81%	69%	77%
	Rangpur	51%	97%	96%	14%	76%	67%	83%
	Sylhet	48%	89%	87%	18%	78%	64%	71%
DRC	Bas-uele	37%	68%	66%	91%	73%	67%	54%
	Equateur	42%	80%	71%	95%	24%	62%	71%
	Haut-katanga	45%	73%	63%	73%	46%	60%	28%
	Haut-lomami	53%	57%	49%	29%	56%	49%	28%
	Haut-uele	42%	75%	68%	81%	44%	62%	39%
	Ituri	51%	79%	74%	82%	30%	63%	52%
	Kasai	43%	72%	63%	25%	34%	47%	47%
	Kasai-central	49%	86%	86%	84%	40%	69%	46%
	Kasai-oriental	43%	83%	79%	74%	59%	67%	40%
	Kinshasa	60%	64%	56%	33%	66%	56%	33%

	Kongo central	55%	58%	54%	40%	58%	53%	18%
	Kwango	48%	91%	86%	95%	16%	67%	79%
	Kwilu	48%	71%	64%	90%	20%	58%	70%
	Lomami	47%	91%	91%	86%	37%	71%	54%
	Lualaba	54%	77%	76%	54%	65%	65%	23%
	Mai-ndombe	47%	85%	77%	92%	36%	67%	56%
	Maniema	57%	89%	85%	65%	65%	72%	32%
	Mongala	40%	87%	65%	78%	39%	62%	48%
	Nord-kivu	43%	91%	84%	94%	0%	63%	94%
	Nord-ubangi	55%	83%	82%	64%	34%	64%	49%
	Sankuru	36%	64%	56%	83%	81%	64%	47%
	Sud-kivu	49%	88%	85%	86%	0%	62%	88%
	Sud-ubangi	60%	75%	71%	69%	16%	58%	59%
	Tanganyka	47%	72%	71%	75%	64%	66%	28%
	Tshopo	41%	71%	67%	67%	52%	60%	30%
	Tshuapa	44%	84%	81%	94%	36%	68%	58%
**Haiti**	Artibonite	60%	78%	70%	67%	62%	67%	18%
	Centre	58%	90%	79%	70%	72%	74%	32%
	Grand'anse	66%	84%	76%	83%	78%	77%	18%
	Nippes	67%	64%	52%	29%	58%	54%	38%
	Nord	68%	73%	56%	42%	68%	61%	31%
	Nord-est	61%	66%	60%	45%	64%	59%	21%
	Nord-ouest	63%	58%	56%	4%	84%	53%	80%
	Ouest	59%	72%	63%	13%	76%	57%	63%
	Sud	56%	75%	49%	20%	98%	60%	78%
	Sud-est	63%	83%	75%	22%	68%	62%	61%
**Kenya**	Central	69%	76%	66%	64%	46%	64%	30%
	Coast	69%	71%	65%	53%	70%	66%	18%
	Eastern	71%	70%	60%	46%	64%	62%	25%
	Nairobi	61%	81%	72%	71%	72%	71%	20%
	North eastern	61%	83%	70%	42%	63%	64%	41%
	Nyanza	62%	69%	52%	31%	92%	61%	61%
	Rift valley	72%	67%	60%	14%	66%	56%	58%
	Western	61%	73%	62%	20%	63%	56%	53%

**Malawi**	Central region	68%	73%	64%	67%	67%	68%	9%
	Northern region	65%	75%	67%	78%	82%	73%	17%
	Southern region	69%	74%	65%	64%	72%	69%	10%
**Namibia**	Caprivi	58%	77%	49%	3%	97%	57%	94%
	Erongo	73%	63%	58%	18%	95%	61%	77%
	Hardap	78%	78%	69%	17%	83%	65%	66%
	Karas	72%	73%	68%	15%	92%	64%	77%
	Kavango	61%	71%	53%	21%	97%	60%	76%
	Khomas	74%	56%	50%	0%	75%	51%	75%
	Kunene	63%	69%	56%	14%	93%	59%	79%
	Ohangwena	66%	83%	68%	21%	100%	68%	79%
	Omaheke	75%	84%	82%	25%	100%	73%	75%
	Omusati	72%	79%	68%	42%	100%	72%	58%
	Oshana	68%	78%	66%	43%	100%	71%	57%
	Oshikoto	70%	70%	57%	18%	95%	62%	77%
	Otjozondjupa	74%	52%	47%	20%	97%	58%	77%
**Nepal**	Central	54%	71%	61%	73%	92%	70%	38%
	Eastern	54%	66%	60%	86%	93%	72%	39%
	Far-western	52%	76%	67%	88%	90%	75%	38%
	Mid-western	53%	71%	64%	88%	90%	73%	37%
	Western	54%	74%	69%	87%	94%	76%	40%
**Rowanda**	East	56%	91%	79%	81%	97%	81%	41%
	Kigali city	70%	56%	48%	69%	92%	67%	44%
	North	60%	84%	75%	80%	100%	80%	40%
	South	64%	91%	86%	87%	93%	84%	29%
	West	62%	90%	83%	77%	98%	82%	36%
**Senegal**	Dakar	79%	64%	62%	22%	67%	59%	57%
	Diourbel	83%	81%	80%	64%	60%	74%	23%
	Fatick	66%	91%	89%	37%	57%	68%	54%
	Kaffrine	47%	86%	86%	45%	72%	67%	41%
	Kaolack	51%	68%	67%	16%	66%	54%	52%
	Kedougou	45%	78%	78%	58%	66%	65%	33%
	Kolda	45%	86%	81%	61%	69%	69%	41%

	Louga	61%	58%	57%	53%	65%	59%	12%
	Matam	63%	73%	70%	58%	66%	66%	15%
	Saint-louis	62%	74%	74%	56%	60%	65%	18%
	Sedhiou	68%	100%	98%	51%	61%	75%	49%
	Tambacounda	61%	69%	66%	77%	66%	68%	16%
	This	61%	69%	68%	46%	67%	62%	23%
	Ziguinchor	70%	87%	86%	35%	64%	68%	52%
**Tanzania**	Arusha	62%	77%	68%	19%	14%	48%	63%
	Dar es salaam	49%	68%	67%	47%	11%	48%	57%
	Dodoma	73%	59%	55%	15%	52%	51%	58%
	Geita	54%	57%	54%	57%	17%	48%	40%
	Iringa	57%	58%	57%	58%	23%	51%	35%
	Kagera	55%	47%	41%	63%	15%	44%	48%
	Kaskazini pemba	59%	85%	78%	76%	29%	65%	56%
	Kaskazini unguja	62%	78%	74%	50%	86%	70%	36%
	Katavi	66%	83%	81%	40%	84%	71%	44%
	Kigoma	49%	80%	70%	82%	14%	59%	68%
	Kilimanjaro	52%	79%	66%	26%	25%	50%	54%
	Kusini pemba	61%	75%	66%	34%	41%	55%	41%
	Kusini unguja	61%	68%	64%	59%	86%	68%	27%
	Lindi	64%	78%	77%	15%	81%	63%	66%
	Manyara	52%	86%	77%	38%	26%	56%	60%
	Mara	53%	86%	78%	37%	35%	58%	51%
	Mbeya	54%	74%	70%	47%	22%	53%	52%
	Mjini magharibi	55%	59%	56%	59%	22%	50%	37%
	Morogoro	57%	65%	62%	56%	25%	53%	40%
	Mtwara	66%	45%	43%	16%	48%	44%	50%
	Mwanza	60%	48%	40%	73%	21%	49%	52%
	Njombe	51%	66%	56%	40%	13%	45%	53%
	Pwani	53%	54%	53%	51%	23%	47%	31%
	Rukwa	49%	44%	40%	54%	4%	38%	50%
	Ruvuma	51%	45%	36%	67%	28%	45%	39%
	Shinyanga	49%	95%	92%	70%	31%	67%	64%

	Simiyu	53%	79%	72%	64%	12%	56%	67%
	Singida	54%	90%	89%	75%	32%	68%	58%
	Tabora	51%	75%	74%	66%	15%	56%	60%
	Tanga	50%	90%	90%	38%	18%	57%	72%
**Uganda**	Central 1	52%	91%	78%	83%	100%	81%	48%
	Central 2	44%	91%	70%	77%	97%	76%	53%
	East central	55%	82%	69%	85%	100%	78%	45%
	Eastern	70%	60%	49%	57%	93%	66%	44%
	Kampala	54%	85%	68%	85%	100%	78%	46%
	North	45%	79%	34%	84%	100%	68%	66%
	Southwest	55%	82%	59%	82%	100%	76%	45%
	West-nile	55%	69%	57%	67%	100%	70%	45%
	Western	64%	93%	72%	94%	100%	85%	36%

Supplementary Table 3: Quality of PHC service delivery at the subnational level in the studied countries

Country	Division	Waiting Time	Provider Availability	Provider Competence	Person Centeredness	Safety	Comprehensive-ness	Average Quality score	Range across quality Subdomains *
Afghanistan	Balkh	89%	35%	28%	75%	28%	65%	53%	61%
	Herat	84%	34%	34%	50%	33%	52%	48%	51%
	Kabul	91%	20%	24%	34%	52%	67%	48%	71%
	Kandahar	100%	34%	19%	1%	55%	75%	47%	99%
	Kunduz	87%	42%	38%	89%	6%	69%	55%	83%
	Nangarhar	97%	48%	34%	45%	17%	78%	53%	80%
	Paktya	94%	54%	30%	22%	23%	73%	49%	72%
Bangladesh	Barisal	-	-	-	-	39%	67%	53%	28%
	Chittagong	-	-	-	-	39%	64%	52%	25%
	Dhaka	-	-	-	-	40%	61%	50%	21%
	Khulna	-	-	-	-	39%	64%	52%	25%
	Mymensingh	-	-	-	-	31%	65%	48%	34%
	Rajshahi	-	-	-	-	44%	68%	56%	24%
	Rangpur	-	-	-	-	45%	50%	47%	5%
	Sylhet	-	-	-	-	33%	68%	51%	35%
DRC	Bas-uele	74%	68%	39%	45%	35%	70%	55%	39%
	Equateur	90%	86%	36%	46%	28%	74%	60%	62%
	Haut-katanga	82%	89%	45%	40%	22%	86%	61%	67%
	Haut-lomami	78%	65%	43%	22%	45%	87%	57%	65%
	Haut-uele	63%	64%	34%	20%	26%	70%	46%	50%
	Ituri	89%	75%	39%	24%	39%	56%	54%	65%
	Kasai	95%	82%	30%	8%	19%	53%	48%	87%
	Kasai-central	73%	78%	39%	45%	30%	68%	56%	48%

	Kasai-oriental	69%	72%	38%	39%	28%	79%	54%	51%
	Kinshasa	44%	69%	48%	22%	29%	79%	48%	57%
	Kongo central	83%	88%	44%	33%	34%	88%	62%	55%
	Kwango	87%	87%	40%	11%	28%	71%	54%	76%
	Kwilu	65%	67%	37%	12%	37%	87%	51%	75%
	Lomami	70%	66%	37%	28%	36%	91%	55%	63%
	Lualaba	65%	63%	47%	40%	34%	91%	56%	57%
	Mai-ndombe	75%	86%	38%	23%	27%	78%	55%	63%
	Maniema	78%	71%	37%	46%	42%	94%	61%	57%
	Mongala	85%	89%	36%	50%	21%	71%	59%	68%
	Nord-kivu	65%	78%	42%	47%	30%	58%	54%	48%
	Nord-ubangi	77%	76%	48%	9%	49%	79%	56%	70%
	Sankuru	88%	87%	35%	30%	27%	49%	53%	61%
	Sud-kivu	75%	65%	40%	33%	36%	81%	55%	48%
	Sud-ubangi	71%	82%	44%	49%	43%	68%	60%	39%
	Tanganyka	80%	87%	33%	31%	42%	52%	54%	56%
	Tshopo	81%	88%	37%	20%	27%	71%	54%	68%
	Tshuapa	82%	88%	38%	41%	30%	69%	58%	58%
**Haiti**	Artibonite	50%	62%	36%	15%	51%	79%	49%	64%
	Centre	66%	70%	33%	7%	56%	88%	53%	81%
	Grand'anse	48%	71%	37%	8%	61%	85%	51%	77%
	Nippes	42%	59%	34%	18%	45%	75%	45%	57%
	Nord	52%	67%	36%	13%	55%	83%	51%	70%
	Nord-est	46%	68%	39%	10%	53%	77%	49%	67%
	Nord-ouest	64%	67%	49%	34%	76%	70%	60%	42%
	Ouest	70%	57%	44%	48%	51%	78%	58%	34%
	Sud	93%	54%	37%	44%	45%	64%	56%	56%
	Sud-est	53%	72%	58%	58%	60%	89%	65%	36%

**Kenya**	Central	30%	60%	36%	9%	55%	81%	45%	72%
	Coast	49%	63%	35%	14%	70%	90%	54%	76%
	Eastern	54%	63%	35%	15%	58%	83%	51%	68%
	Nairobi	53%	72%	32%	20%	56%	83%	52%	63%
	North eastern	73%	59%	44%	39%	66%	89%	62%	50%
	Nyanza	84%	34%	46%	71%	78%	72%	64%	50%
	Rift valley	65%	77%	50%	70%	71%	71%	67%	27%
	Western	75%	75%	51%	68%	67%	76%	69%	25%
**Malawi**	Central region	49%	43%	38%	51%	50%	75%	51%	37%
	Northern region	46%	42%	37%	41%	46%	77%	48%	40%
	Southern region	49%	39%	37%	43%	51%	72%	48%	35%
**Namibia**	Caprivi	30%	70%	52%	29%	81%	93%	59%	64%
	Erongo	59%	49%	52%	76%	65%	77%	63%	28%
	Hardap	49%	69%	51%	45%	86%	84%	64%	41%
	Karas	65%	59%	48%	45%	78%	87%	64%	42%
	Kavango	34%	48%	49%	30%	75%	94%	55%	64%
	Khomas	49%	69%	51%	84%	86%	55%	66%	37%
	Kunene	73%	76%	59%	79%	78%	86%	75%	27%
	Ohangwena	8%	55%	59%	63%	70%	93%	58%	85%
	Omaheke	58%	79%	67%	79%	91%	90%	77%	33%
	Omusati	39%	59%	49%	35%	69%	92%	57%	57%
	Oshana	25%	62%	60%	65%	59%	84%	59%	59%
	Oshikoto	34%	74%	64%	53%	54%	95%	62%	61%
	Otjozondjupa	46%	48%	47%	26%	61%	79%	51%	53%
**Nepal**	Central	96%	31%	26%	27%	42%	76%	50%	70%
	Eastern	90%	47%	31%	40%	44%	76%	55%	59%
	Far-western	94%	58%	34%	37%	42%	79%	57%	60%

	Mid-western	91%	45%	32%	27%	49%	74%	53%	64%
	Western	96%	41%	33%	37%	49%	75%	55%	63%
**Rowanda**	East	31%	41%	40%	13%	21%	79%	37%	66%
	Kigali city	52%	51%	45%	51%	31%	57%	48%	26%
	North	17%	34%	41%	27%	24%	76%	37%	59%
	South	43%	33%	35%	19%	32%	83%	41%	64%
	West	42%	37%	44%	18%	24%	78%	40%	60%
**Senegal**	Dakar	71%	63%	42%	24%	67%	70%	56%	47%
	Diourbel	99%	71%	42%	10%	81%	77%	63%	89%
	Fatick	94%	70%	42%	10%	72%	58%	58%	84%
	Kaffrine	71%	61%	47%	4%	53%	52%	48%	67%
	Kaolack	78%	69%	48%	29%	58%	53%	56%	49%
	Kedougou	86%	77%	49%	8%	45%	56%	53%	78%
	Kolda	76%	71%	44%	6%	49%	51%	50%	70%
	Louga	63%	49%	34%	1%	59%	74%	47%	73%
	Matam	72%	24%	28%	1%	75%	74%	46%	74%
	Saint-louis	43%	49%	33%	2%	68%	73%	45%	71%
	Sedhiou	91%	72%	38%	1%	79%	64%	58%	90%
	Tambacounda	62%	59%	32%	0%	69%	81%	50%	81%
	This	74%	60%	43%	20%	61%	64%	54%	54%
	Ziguinchor	97%	74%	37%	12%	74%	66%	60%	85%
**Tanzania**	Arusha	74%	76%	45%	44%	63%	59%	60%	32%
	Dar es salaam	59%	82%	42%	68%	35%	72%	60%	47%
	Dodoma	76%	76%	40%	44%	52%	56%	57%	36%
	Geita	71%	87%	35%	13%	26%	71%	51%	74%
	Iringa	42%	60%	44%	78%	30%	75%	54%	48%
	Kagera	76%	70%	45%	52%	37%	74%	59%	39%

	Kaskazini pemba	28%	72%	46%	77%	35%	76%	56%	49%
	Kaskazini unguja	71%	74%	39%	19%	27%	76%	51%	57%
	Katavi	22%	81%	47%	65%	66%	87%	61%	65%
	Kigoma	46%	74%	35%	67%	31%	83%	56%	52%
	Kilimanjaro	28%	56%	40%	41%	24%	83%	45%	59%
	Kusini pemba	84%	82%	35%	33%	54%	68%	59%	51%
	Kusini unguja	67%	82%	38%	23%	25%	74%	51%	59%
	Lindi	58%	77%	44%	67%	58%	89%	65%	45%
	Manyara	66%	61%	34%	57%	42%	63%	54%	32%
	Mara	54%	79%	48%	27%	47%	57%	52%	52%
	Mbeya	69%	74%	33%	24%	36%	61%	49%	50%
	Mjini magharibi	68%	59%	35%	46%	50%	78%	56%	43%
	Morogoro	61%	50%	36%	16%	61%	70%	49%	54%
	Mtwara	72%	57%	33%	50%	41%	66%	53%	39%
	Mwanza	41%	68%	38%	54%	44%	71%	53%	33%
	Njombe	55%	65%	43%	78%	36%	68%	58%	42%
	Pwani	41%	71%	47%	44%	36%	70%	52%	35%
	Rukwa	81%	76%	45%	27%	40%	60%	55%	54%
	Ruvuma	62%	43%	29%	19%	36%	75%	44%	56%
	Shinyanga	50%	76%	36%	48%	39%	65%	52%	40%
	Simiyu	64%	69%	37%	38%	34%	58%	50%	35%
	Singida	50%	74%	48%	81%	34%	70%	60%	47%
	Tabora	39%	64%	39%	30%	44%	63%	46%	34%
	Tanga	50%	70%	52%	49%	34%	80%	56%	46%
**Uganda**	Central 1	64%	46%	44%	20%	27%	81%	47%	61%
	Central 2	69%	31%	54%	64%	23%	65%	51%	46%

	East central	53%	46%	56%	71%	27%	70%	54%	44%
	Eastern	50%	51%	56%	72%	37%	86%	59%	49%
	Kampala	47%	36%	60%	82%	23%	76%	54%	59%
	North	22%	21%	39%	36%	17%	61%	33%	44%
	Southwest	33%	51%	40%	39%	36%	72%	45%	39%
	West-nile	66%	49%	48%	65%	22%	64%	52%	44%
	Western	34%	42%	43%	39%	24%	73%	43%	49%
