# Supplementary material for: Capacity and quality of maternal and child health services delivery at the subnational primary healthcare level in relation to intermediate health outputs: a cross-sectional study of 12 low-income and middle-income countries
Source: BMJ Open. 2023 Jan 31;13(1):e065223. doi: 10.1136/bmjopen-2022-065223 (PMC9890757; doi:10.1136/bmjopen-2022-065223)
Supplement: Author's manuscript [file bmjopen-2022-065223.draft_revisions.pdf]

BMJ Open

BMJ Open is committed to open peer review. As part of this commitment we make the peer review history of every article we publish publicly available.

When an article is published we post the peer reviewers' comments and the authors' responses online. We also post the versions of the paper that were used during peer review. These are the versions that the peer review comments apply to.

The versions of the paper that follow are the versions that were submitted during the peer review process. They are not the versions of record or the final published versions. They should not be cited or distributed as the published version of this manuscript.

BMJ Open is an open access journal and the full, final, typeset and author-corrected version of record of the manuscript is available on our site with no access controls, subscription charges or pay-per-view fees (http://bmjopen.bmj.com).

If you have any questions on BMJ Open's open peer review process please email info.bmjopen@bmj.com

BMJ Open

Not One-Solution-Fits-All: Subnational Heterogeneity in Primary Health Care Service Delivery in 12 Low-and Middle-Income Countries

Journal:	*BMJ Open*
Manuscript ID	bmjopen-2022-065223
Article Type:	Original research
Date Submitted by the Author:	05-Jun-2022
Complete List of Authors:	Ramadan, Marwa; World Bank Group<br>Gutierrez, Jose Carlos; World Bank Group<br>Feil, Cameron; World Bank Group<br>Bolongaita, Sarah; World Bank Group<br>Bernal, Oscar; World Bank Group<br>Villar-Uribe , Manuela; World Bank Group
Keywords:	PRIMARY CARE, Quality in health care < HEALTH SERVICES ADMINISTRATION & MANAGEMENT, PUBLIC HEALTH, International health services < HEALTH SERVICES ADMINISTRATION & MANAGEMENT

SCHOLARONE™
Manuscripts

I, the Submitting Author has the right to grant and does grant on behalf of all authors of the Work (as defined in the below author licence), an exclusive licence and/or a non-exclusive licence for contributions from authors who are: i) UK Crown employees; ii) where BMJ has agreed a CC-BY licence shall apply, and/or iii) in accordance with the terms applicable for US Federal Government officers or employees acting as part of their official duties; on a worldwide, perpetual, irrevocable, royalty-free basis to BMJ Publishing Group Ltd ("BMJ") its licensees and where the relevant Journal is co-owned by BMJ to the co-owners of the Journal, to publish the Work in this journal and any other BMJ products and to exploit all rights, as set out in our licence.

The Submitting Author accepts and understands that any supply made under these terms is made by BMJ to the Submitting Author unless you are acting as an employee on behalf of your employer or a postgraduate student of an affiliated institution which is paying any applicable article publishing charge ("APC") for Open Access articles. Where the Submitting Author wishes to make the Work available on an Open Access basis (and intends to pay the relevant APC), the terms of reuse of such Open Access shall be governed by a Creative Commons licence – details of these licences and which Creative Commons licence will apply to this Work are set out in our licence referred to above.

Other than as permitted in any relevant BMJ Author's Self Archiving Policies, I confirm this Work has not been accepted for publication elsewhere, is not being considered for publication elsewhere and does not duplicate material already published. I confirm all authors consent to publication of this Work and authorise the granting of this licence.

For peer review only

Not One-Solution-Fits-All: Subnational Heterogeneity in Primary Health Care Service Delivery in 12 Low-and Middle-Income Countries

Authors and affiliations

Marwa Ramadan[1]*, Jose Carlos Gutierrez[1], Cameron Feil[1], Sarah Bolongaita[1], Oscar Bernal[1], Manuela Villar Uribe[1]

[1]Health Nutrition and Population Global Practice, World Bank Group, Washington, DC, USA.

***Corresponding Author**

Marwa Ramadan

Health Nutrition and Population Global Practice, World Bank Group, Washington, DC, USA

mramadan@worldbank.org

Keywords

Primary healthcare; healthcare quality; measurement; performance assessment; subnational; Low- and Middle-income countries

For peer review only - http://bmjopen.bmj.com/site/about/guidelines.xhtml

Abstract

Objectives: To examine the capacity and quality of PHC service delivery at the subnational level in 12 Low-and Middle-Income Countries (LMICs) and its association with intermediate health outputs such as coverage and access to care.

Design: Observational cross-sectional study using matched subnational data from service provision assessment surveys and demographic health surveys

Settings: 12 LMICs (Afghanistan, Bangladesh, Democratic Republic of Congo, Haiti, Kenya, Malawi, Namibia, Nepal, Rwanda, Senegal, Tanzania, Uganda)

Participants: 138 sub-national areas with available survey data from 2007-2019.

Outcomes: Eight intermediate maternal and child health outcomes/outputs were explored: 1) met need for family planning by modern methods; 2) attendance of four or more antenatal care visits; 3) perceived financial barriers to care; 4) perceived geographic barriers to care; 5) DPT3 coverage; 6) DPT dropout-rate; 7) care seeking for pneumonia; and 8) ORS coverage

Results: Overall, moderate to poor PHC performance was observed across the 12 countries, with substantial heterogeneity between the different subnational areas in the same country as well as within the same subnational area across both capacity and quality subdomains. The analysis of the relationship between PHC service delivery and child health outcomes revealed significant associations between supervision and care-seeking for pneumonia, as well as associations of several measures of capacity and quality with DPT3 immunization. The analysis of maternal health outcomes yielded only a few statistically significant results at the $p<0.05$ level, however, none remained significant after adjusting for other covariates.

Conclusion: The results of this descriptive analysis illustrate the heterogeneity in the capacity and quality of PHC service delivery within LMICs. Countries seeking to strengthen their PHC systems could improve PHC monitoring at the subnational level to better understand subnational bottlenecks in service delivery.

For peer review only - http://bmjopen.bmj.com/site/about/guidelines.xhtml

Strengths and limitations of this study:

- Existing literature focused on the relationship between Primary Healthcare (PHC) capacity, quality, and performance at the national levels. However, this study bridges a gap by focusing on sub-national PHC service delivery and its association with improved health outputs
- The methodology utilized in the present study demonstrates how publicly available survey data such as the Demographic Health Survey (DHS) and the Service Provision Assessment (SPA) survey can be valuable tools in highlighting bottlenecks and disparities in PHC service delivery at the subnational level
- As the study is observational and cross-sectional, the association presented should not be interpreted as causal given the potential for an ecological bias
- Although the sample was constructed such that the time between the SPA and DHS surveys in each country was less than 5 years, it is possible that changes in time-varying factors could have influenced the outcomes under study.
- There is a potential for omitted variable bias through other factors that may influence the capacity, quality, and performance variables under study but are unmeasured in the SPA and DHS surveys

For peer review only

Introduction

Despite renewed global commitment to primary healthcare (PHC) in recent years, the measurement of primary health care (PHC) capacity and quality remains a challenge for policymakers seeking to improve the performance of health services. The transition from the Millennium Development Goals (MDGs) to Sustainable Development Goals (SDGs) in 2016 marked a significant shift in the global health agenda. SDG 3, "Achieving Universal Healthcare Coverage," was a call to action to strengthen health systems, shifting the global health agenda away from vertically planned health programs and initiatives. In 2018, forty years after the Alma-Alta Declaration which identified primary health care (PHC) as the pathway to achieving health for all, global actors, governments, and organizations re-affirmed a global commitment to PHC with the issuance of the Astana Declaration (1).

More significant efforts are required to improve primary health care performance in low-and-middle-income countries (2). Performance measurement has the potential to catalyze PHC improvement by identifying bottlenecks (3,4). The Primary Health Care Performance Initiative (PHCPI), a global collaborative dedicated to measuring and improving PHC systems in LMICs, developed a conceptual framework illustrating the relationships between financing, inputs, and core functions of the PHC system (2). In 2018, PHCPI operationalized the Conceptual Framework into the Vital Signs Profile, which uses qualitative and quantitative indicators to provide a comprehensive understanding of PHC performance in a county (3).

This paper explores the relationship between PHC system capacity, quality, and performance at the sub-national level using quantitative indicators from the Vital Signs Profile. The relationship between capacity and health system performance has long been studied. Donabedian captured the causal chain of inputs to outputs, commonly defined as the 'input-process-output-model', in 1966. More recent efforts by Randhawa (4) and Veillard et al.(5) have demonstrated the relationship between inputs and outcomes at the PHC level. The importance of receiving high-quality health care services has also been documented, as Tanahashi (6) distinguished between contact (receiving health services) and effective service coverage (receiving high-quality health services). People living in LMICs are also especially vulnerable to low-quality health service delivery (7–9) With the introduction of SDG 3, integrating quality within coverage measures has been increasingly emphasized (10).

The relationship between capacity, quality, and performance has been explored extensively at the international and national levels. For example, PHC capacity, including the availability of providers, governance, and financing, has been found to impact PHC-related outcomes in LMICs at the national level (11–15). Several studies have also identified relationships between elements of high-quality PHC and performance at the national level (2,16–19). However, more opportunities exist for focusing on sub-national PHC service delivery and its association with improved health outputs. This paper aims to fill some of those measurement gaps by examining the capacity and quality of PHC system at the subnational level in 12 LMICs and their association with intermediate health outputs such as coverage and access to services.

Methods

Data sources

In this cross-sectional descriptive analysis, service provision assessment (SPA) surveys and Demographic health surveys (DHS) were the main sources of data on PHC service delivery and PHC intermediate population health outputs, respectively in 12 low-and Middle-income countries (Afghanistan,

Bangladesh, DRC (democratic republic of Congo), Haiti, Kenya, Malawi, Namibia, Nepal, Rwanda, Senegal, Tanzania, Uganda). SPA surveys are comprehensive facility assessments that aim to collect information on the availability of services at the facility level, the readiness of facilities to provide these services, the compliance with acceptable standards of care, and whether clients and service providers are satisfied with the service delivery process (20). SPA surveys usually involve random selection of 400-700 facilities from a comprehensive sampling list of all public and private health facilities in a country or alternatively a complete census of facilities in the country. Facilities are usually categorized by facility type, managing authority (public and non-public), as well as subnational region; sampling and sample size ensure representativeness across each of these categories. Weights are applied during the analysis of these surveys to ensure that the contribution of selected facilities is proportional to their distribution in the country. On the other hand, DHS are nationally representative household surveys and a vital source of information on population, health, and nutrition indicators in more than 90 countries. The standard DHS survey is conducted every five years using a large sample size (5000-30,000 households per survey), and usually utilizes a two-stage sampling methodology based on the availability of census information(21). The DHS sampling and sample size ensure representativeness at the subnational region level. Supplementary Table 1 provides additional information on the sampling structure and the timeframe for data collection in each of the studied countries [See Supplementary Methods]

Metrics

Guided by the PHCPI framework and survey data availability, the capacity of the PHC system was assessed using the following five indicators: 1) availability of basic facility Infrastructure; 2) the presence of recent supervision within the past 6 months; 3) the provision of feedback by supervisors; 4) the provision of outreach services; and 5) absence of PHC fees defined as free immunization, RMNCH, non-communicable diseases and HIV services. Similarly, whenever data was available, PHCPI's Vital Signs Profile (VSP) guided the assessment of PHC quality of care through the following six indicators: 1) waiting time of less than 60 minutes; 2) availability of providers defined as an average consultation time of more than 10 minutes; 3) competence of providers defined as the average of antenatal care, family planning and sick child quality scores based on WHO guidelines; 4) person-centeredness defined as the percent of clients who were told their diagnosis; 5) safety defined as the presence of adequate infection control practices and waste disposal at the facility; and 6) comprehensiveness of PHC services defined as the average availability of tracer items for RMNCH, infectious diseases and non-communicable diseases. Additional information on PHCPI's framework is provided in Supplementary Figure 1 [See Supplementary Methods]

Additionally, eight intermediate maternal and child health outcomes/outputs were explored in accordance with the PHCPI vital signs profile and consistent reporting of DHS indicators in the studied countries. Specifically, we assessed the following maternal and child health outputs: 1) met need for family planning by modern methods; 2) attendance of four or more antenatal care visits; 3) perceived financial barriers to care; 4) perceived geographic barriers to care; 5) DPT3 coverage; 6) DPT dropout-rate; 7) care seeking for pneumonia; and 8) ORS coverage. Other PHC-related outputs (e.g., non-communicable diseases, tuberculosis and HIV) were not assessed due to the limited availability of sub-national data in the studied countries. Detailed definition of each of the capacity, quality and output indicators are provided in Supplementary Table 2 [See Supplementary Methods]

For peer review only - http://bmjopen.bmj.com/site/about/guidelines.xhtml

Data analysis

Population and health facility data were linked at the first administrative level (e.g., region, province…etc.) whenever data was available and the gap between a population-based survey and a health facility survey was less than five years. In situations where substantial variation existed at the first administrative level between the two types of surveys, geographic coordinates of health facilities and DHS clusters were input into geographic information system (GIS) software (ArcGIS) for matching. Specifically, in the Democratic Republic of Congo, DHS data in 2013 were reported for 11 formerly defined provinces, while 2017 SPA data were reported for 26 newly defined provinces. ArcGIS was used to match subnational areas in both types of surveys. In addition, in Afghanistan, the SPA survey was only conducted in seven urban subnational areas, so the match process with DHS was limited to these areas [See Supplementary Figure 2 in Supplementary Methods]

To compute subnational estimates, we applied the recommended SPA weights at the facility, provider, and visit levels in the 12 studied countries. We then computed national averages based on the assumption that all subnational areas were equally weighted in each of the studied countries. In addition, we examined the subnational association between the computed service delivery sub-domains and DHS intermediate maternal and child health outputs. Given the hierarchical nature of the examined population and health facility data, we utilized a two-level fixed-effects model to account for possible clustering of the observations at the country level. We also adjusted for the following subnational population and facility characteristics: proportion of private facilities, proportion of hospitals, proportion of urban households, proportion of population in the 5th economic quintile (highest), and proportion of women with secondary or more education.

Patient and public involvement

This study relied on secondary analysis of publicly available datasets. Therefore, patients and the public were not involved in the formulation of this research.

Results

I. PHC service delivery at the national and subnational levels

In the present study, the capacity and quality of PHC service delivery were analyzed in 138 subnational areas in 12 LMICs. Substantial heterogeneity was observed across countries in the capacity subdomain. For example, the average availability of facility infrastructure (basic amenities and infection prevention) was as high as 80% in Afghanistan and as low as 48 % in DRC. Most facilities in the analyzed subnational areas reported the presence of recent supervision, with an average ranging from 94% in Bangladesh to 70% in Tanzania, while supervisor's feedback was provided in a relatively fewer number of facilities, with the highest average reported in Bangladesh (91%) and the lowest in Afghanistan (57 %). Meanwhile, the provision of facility outreach services varied largely across the studied countries, with 84 % of facilities in Nepal providing outreach services versus only 14 % of facilities in the analyzed subnational areas in Afghanistan. Similarly, large variation in charging PHC fees was observed across the sample; 99% of facilities in the analyzed subnational areas of Uganda did not charge fees for PHC services versus only 32 % in Tanzania. Table 1 shows the average score of individual capacity sub-domains in the analyzed subnational areas across the 12 studied countries.

When the quality-of-service delivery was assessed, most countries had moderate to poor scores in all quality sub-domains except waiting time and comprehensiveness, where some countries performed well. As with capacity, cross-country variation in quality sub-domains was observed. For example, the

For peer review only - http://bmjopen.bmj.com/site/about/guidelines.xhtml

average waiting time was less than 60 minutes in more than 90 % of the facilities in the analyzed subnational areas of Afghanistan and Nepal versus only 37 % of the analyzed facilities in Rwanda. However, the average consultation time was more than 10 minutes in only 38 % of the analyzed Afghan facilities. Competence of providers was one of the quality sub-domains with the lowest average scores across the studied countries ranging from 30 % on average, for the analyzed facilities in Afghanistan to 54 % in Namibia. Similarly, person centeredness scored poorly in most countries with an average score as low as 9 % for the analyzed facilities in Senegal versus 54 % in Uganda. Meanwhile, at least two thirds of the analyzed facilities offered comprehensive PHC services including RMNCH, non-communicable and infectious disease services in all the studied countries (Table 1).

Table 1: The capacity and quality of PHC service delivery in the studied countries (average scores of subnational areas)

Domain	Subdomain	Country											
		AF18*	BD17	DRC18	HT17	KE10	MW13	NM09	NP15	RW07	SN17	TZ15	UG07
Capacity	Facility Infrastructure	80%	51%	48%	62%	66%	67%	69%	53%	63%	62%	56%	55%
	Recent Supervision	77%	94%	77%	74%	74%	74%	72%	72%	82%	77%	70%	81%
	Supervisor Feedback	57%	91%	72%	64%	63%	65%	61%	64%	74%	76%	65%	62%
	Outreach Services	14%	18%	73%	39%	43%	70%	20%	84%	79%	48%	50%	79%
	Not charging PHC Fees	50%	67%	42%	73%	67%	74%	94%	92%	96%	65%	32%	99%
	**Capacity score**	55%	65%	61%	60%	61%	70%	63%	73%	79%	64%	53%	76%
Quality	Waiting Time < 60 minutes	92%	-	76%	58%	61%	48%	44%	94%	37%	77%	57%	49%
	Provider Availability	38%	-	78%	65%	63%	41%	63%	44%	39%	62%	70%	42%
	Provider Competence	30%	-	39%	40%	41%	37%	54%	31%	41%	40%	40%	49%
	Person Centeredness	45%	-	31%	25%	38%	45%	54%	33%	26%	9%	46%	54%
	Safety	31%	39%	32%	55%	65%	49%	73%	45%	26%	65%	41%	26%
	Comprehensiveness	68%	63%	74%	79%	81%	75%	85%	76%	75%	65%	71%	72%
	**Quality**	50%	51%	55%	56%	61%	49%	62%	54%	41%	53%	54%	47%

AF18: Afghanistan 2018, BD17: Bangladesh 2017, DRC18: democratic republic of Congo 2018, HT17: Haiti 2017, KE10: Kenya 2010, MW13: Malawi 2013, NM09: Namibia 2009, NP15: Nepal 2015, RW07: Rwanda 2007, SN17: Senegal 2017, TZ15: Tanzania 2015, UG07: Uganda 2007
* Estimates represent the average of urban subnational data for which SPA and DHS data were available

Subnational analysis of service delivery sub-domains revealed the presence of geographic disparities at the first administrative level in both PHC capacity and quality sub-domains. For example, large subnational variation was observed in the average availability of facility infrastructure in Senegal, ranging from 83 % in Diourbel region to 45 % in Kedougou region. Similarly, considerable subnational variation was observed in the provision of outreach services in Haiti, ranging from 45 % in North-East to 5 % in North-West department. Charging of PHC fees in DRC was also another example of administrative

For peer review only - http://bmjopen.bmj.com/site/about/guidelines.xhtml

variation in PHC capacity, where 81 % of facilities in Sankuru province provided free PHC services versus none of the facilities in North-Kivu or South-Kivu provinces. Similarly, subnational variation was observed in most of the examined quality sub-domains. For example, the range of variation across the different subnational areas in Tanzania was more than 60 % points for waiting time. In addition, the range of variation was more than 50 % for availability of providers across the different subnational areas in Senegal. Furthermore, person centered care ranged from 89 % in Kunduz province to only 1 % in Kandahar province in Afghanistan. Meanwhile, the lowest subnational variations were observed in provider competence sub-domain, where the majority of subnational areas scored below 50 % in the 12 studied countries.

Not only did we observe variations within the same country across different subnational areas, but also within the same subnational area across both quality and capacity sub-domains. For example, the range of variation across different capacity sub-domains was more than 50 % points in the majority of the analyzed sub-national areas in Afghanistan, Bangladesh and Namibia. Most of these variations were derived by the difference in the capacity of the facilities in providing outreach services and offering free PHC services. Similarly, several subnational areas in Afghanistan, DRC and Senegal showed substantial variation across the different quality sub-domains. For example, the range of variation across quality sub-domains in Kandahar, Afghanistan was 99 %, where all facilities had a waiting time of less than 60 minutes; however only 1 % provided person centered care. Supplementary Table 3 and Supplementary Table 4 provide detailed information on the capacity and quality scores in each of the analyzed 138 subnational areas [See Supplementary Results]

II. The association between PHC service delivery sub-domains and intermediate health outputs

Examining the relationship between subnational PHC capacity and maternal health outputs, no statistically significant association was found between the capacity of facilities and ANC coverage after adjustment for subnational characteristics. Meanwhile, a statistically significant association was observed between the general availability of basic facility infrastructure and perceived economic and geographic barriers to care. Specifically, a 10 % point increase in the average availability of basic infrastructure at the subnational level was associated with 3 % lower perceived financial barriers to care [b = -0.35, p < 0.05] and 4 % lower perceived geographic barriers to care [b = -0.39, p < 0.05]. However, the associations were not statistically significant after adjustment for subnational facility and population characteristics (Table 2). Coverage of family planning services was also negatively associated with the provision of outreach services. However, this paradoxical association was no longer significant in the multivariate specification. Meanwhile, no statistically significant association was found between any of the examined PHC quality sub-domains and maternal health outputs (Table 2).

Table 2: The association between PHC service delivery subdomains and maternal health outputs at the subnational level

Sub-domain			ANC4		Family planning		Cost Barrier		Geographic Barrier	
			Bivariate	Multivariate	Bivariate	Multivariate	Bivariate	Multivariate	Bivariate	Multivariate
Capacity	Facility Infrastructure	β	0.23	0.11	0.29	0.16	-0.35*	-0.23	-0.39*	-0.16
		SE	0.13	0.13	0.16	0.19	0.15	0.14	0.15	0.16
	Supervision in the past 6 months	β	-0.09	0.02	-0.12	0.01	0.05	-0.02	0.00	-0.14
		SE	0.08	0.08	0.09	0.10	0.09	0.08	0.10	0.09
	Supervisor Feedback	β	-0.06	0.01	-0.06	0.03	-0.03	-0.09	-0.02	-0.11
		SE	0.08	0.07	0.09	0.09	0.09	0.07	0.09	0.08

For peer review only - http://bmjopen.bmj.com/site/about/guidelines.xhtml

	Outreach Services	β	-0.07	0.00	-0.13*	-0.09	0.04	-0.01	0.09	0.04
		SE	0.05	0.04	0.06	0.06	0.06	0.05	0.06	0.05
	Not charging PHC fees	β	0.01	0.01	-0.01	0.00	-0.02	0.00	0.02	0.06
		SE	0.05	0.04	0.06	0.06	0.06	0.05	0.06	0.05
**Quality**	Waiting time < 60 minutes	β	-0.01	0.01	-0.11	-0.10	0.09	0.05	0.11	0.07
		SE	0.06	0.05	0.08	0.07	0.07	0.06	0.08	0.06
	Provider Competence	β	0.07	0.00	0.04	0.01	-0.28	-0.10	-0.26	-0.10
		SE	0.15	0.13	0.19	0.18	0.19	0.14	0.19	0.15
	Provider Availability	β	-0.04	-0.08	-0.16	-0.15	-0.11	-0.13	0.02	0.03
		SE	0.09	0.07	0.11	0.10	0.10	0.08	0.11	0.08
	Person centeredness	β	-0.01	-0.04	0.01	-0.02	-0.06	-0.01	-0.02	0.02
		SE	0.05	0.04	0.06	0.06	0.06	0.05	0.07	0.05
	Safety	β	0.09	0.06	0.01	-0.03	0.20	0.13	0.11	0.10
		SE	0.08	0.07	0.11	0.11	0.11	0.08	0.11	0.09
	Comprehensiveness	β	0.09	0.09	-0.05	-0.02	-0.20	-0.09	-0.14	-0.05
		SE	0.10	0.08	0.11	0.11	0.11	0.08	0.12	0.09

* p<0.05

In multivariate models, results were adjusted for proportion of private facilities, hospitals, urban households, population in the 5th economic quintile and women with secondary education or more at the sub-national level

Afghanistan was excluded from the analysis for sub-national sample limitation

On the other hand, PHC service delivery sub-domains were more likely to be statistically significantly associated with child health outputs. For example, a 10 % point increase in the availability of basic facility infrastructure was associated with an increase of 5 % in DPT3 immunization coverage [b =0.45, p<0.05] and a decrease of 3% in DPT-dropout rates [b = - 0.30, p<0.05]. Additionally, recent facility supervision [b=0.34, p<0.01] and provision of feedback by supervisors [b=0.28, p<0.05] were each associated with increased care seeking for pneumonia after adjustment for facility and population characteristics. Surprisingly, significantly lower immunization coverage and higher dropout rates were observed in subnational areas where facilities did not charge PHC fees, even after adjustment for facility and population characteristics (Table 3). Meanwhile, of the examined quality sub-domains, only comprehensiveness showed a statistically significant association with subnational DPT3 immunization coverage and lower drop-out rates. Specifically, a 10 % point increase in the comprehensiveness of PHC services was associated with 2 % improvement in DPT3 coverage and 2 % lower DPT-dropout rates at the subnational level [b= 0.22, p<0.05 and b=-0.21, p<0.01 respectively] (Table 3).

Table 3: The association between PHC service delivery subdomains and child health outputs at the subnational level

**Sub-domain**			**DPT3**		**DPT_ dropout**		**Pneumonia care seeking**		**ORS coverage**	
			Bivariate	Multivariate	Bivariate	Multivariate	Bivariate	Multivariate	Bivariate	Multivariate
**Capacity**	Facility Infrastructure	β	0.54**	0.45*	-0.37**	-0.30*	0.18	0.23	-0.02	0.01
		SE	0.15	0.18	0.12	0.14	0.18	0.22	0.12	0.15
	Supervision in the past 6 months	β	-0.04	0.08	0.03	-0.07	0.23*	0.34**	0.08	0.08
		SE	0.09	0.10	0.07	0.08	0.11	0.12	0.07	0.08
	Supervisor Feedback	β	-0.03	0.05	0.01	-0.05	0.22*	0.28**	0.10	0.09
		SE	0.09	0.09	0.07	0.07	0.10	0.11	0.07	0.07
	Outreach Services	β	-0.07	-0.02	0.06	0.01	-0.04	-0.05	0.05	0.04
		SE	0.06	0.06	0.04	0.05	0.07	0.07	0.05	0.05

For peer review only - http://bmjopen.bmj.com/site/about/guidelines.xhtml

	Not charging PHC fees	β	-0.14*	-0.17**	0.07	0.09	0.09	0.09	0.08	0.07
		SE	0.06	0.06	0.05	0.05	0.07	0.07	0.05	0.05
Quality	Waiting time < 60 minutes	β	-0.12	-0.11	0.08	0.07	0.06	0.06	-0.03	-0.03
		SE	0.07	0.07	0.06	0.05	0.08	0.08	0.06	0.06
	Provider Competence	β	0.09	0.03	-0.06	-0.02	0.13	0.14	-0.22	-0.19
		SE	0.19	0.18	0.14	0.14	0.21	0.21	0.14	0.14
	Provider Availability	β	-0.18	-0.17	0.13	0.12	0.11	0.11	-0.02	-0.06
		SE	0.10	0.10	0.08	0.08	0.12	0.12	0.08	0.08
	Person centeredness	β	-0.08	-0.10	0.06	0.08	0.04	0.03	-0.01	-0.02
		SE	0.06	0.06	0.05	0.05	0.07	0.07	0.05	0.05
	Safety	β	0.01	0.03	-0.03	-0.04	0.07	0.07	-0.09	-0.07
		SE	0.11	0.11	0.08	0.08	0.12	0.13	0.09	0.09
	Comprehensiveness	β	0.23*	0.22*	-0.22**	-0.21**	-0.10	-0.11	0.03	0.03
		SE	0.11	0.10	0.08	0.08	0.13	0.13	0.09	0.08

Standard errors in parentheses
* p<0.05, ** p<0.01
In multivariate models, results were adjusted for proportion of private facilities, hospitals, urban households, population in the 5th economic quintile and women with secondary education or more at the subnational level
Afghanistan was excluded from the analysis for subnational sample limitation

Discussion

Overall, the results of the descriptive analysis illustrate the heterogeneity in the capacity and quality of PHC service delivery within LMICs. The analysis of maternal health outcomes yielded only a few statistically significant results at the p<0.05 level, however, none remained significant after adjusting for other covariates. This suggests that factors outside health facilities' control may play a greater role in determining mothers' care seeking decisions for antenatal care and family planning, and in shaping perceptions of cost and geographic barriers to care.

For example, individual- and household-level factors such as the mother's education and socioeconomic status are associated with the use of family planning and antenatal care services. Nonetheless, supply-side constraints at the health system level such as supply chain challenges may also contribute to stockouts of essential FP commodities (22). Analysis at levels more granular than the subnational area may yield further insight into the relationship of supply-side PHC characteristics and maternal health outcomes.

Meanwhile, the analysis of child health outcomes revealed significant associations between supervision and care-seeking for pneumonia, as well as associations of several measures of capacity and quality with DPT3 immunization. Supervision in the last 6 months and supervisor feedback are considered measures of PHC capacity in the PHCPI framework, and it is hypothesized that measures of capacity operate through improved quality of health services. In the case of care-seeking for pneumonia, it is possible that facilities with greater capacity and implementation of supervision deliver care perceived to be of higher quality, thus increasing mothers' likelihood of seeking healthcare for their child at the facility. One review finds inconclusive but promising evidence that supportive supervision can improve quality of care, noting that supervision alone is insufficient; health workers require training and the availability of medicines, diagnostics and equipment to provide quality care (23). Patient perceptions of a facility's quality of care also influence utilization and care seeking (24–30). There is also ample literature demonstrating the influence of various household and individual-level factors such as cultural beliefs, disease severity, geographic location, wealth, education, and socioeconomic status on care-seeking behavior (31,32)

For peer review only

For peer review only - http://bmjopen.bmj.com/site/about/guidelines.xhtml

Finally, our results found several associations with DPT3 immunization and dropout that generally align with existing literature (facility infrastructure and comprehensiveness) and in the case of user fees, counter to what might be expected. In line with our findings, prior research in Haiti found that both infrastructure quality and the quality-of-service delivery were associated increased utilization of primary care services, including ANC and vaccination (33). Similarly, a study in rural India found that children in districts where more villages had a healthcare subcenter were more likely to receive at least one dose of DPT3, though no association with DPT3 dropout rates was observed (34). The results above also suggest that comprehensiveness, a measure of PHC quality, is also associated with better immunization outcomes. This is in line with prior studies documenting that children of mothers who are counseled during vaccination sessions are more likely to complete all three DPT doses (25).

One possible explanation behind the counterintuitive negative association between the absence of user fees and DPT3 immunization is that the estimated association may be confounded by other factors determining both user fee exemptions and immunization coverage. For example, subnational areas that are generally poorer and have greater barriers to care may be more likely adopt policies exempting user fees, yet those characteristics may also result in lower immunization rates. A second explanation under the interpretation that the observed association is unconfounded may be related to potential unintended consequences of user fee removal. While the benefits of removing user fees are well documented in boosting health service utilization (35–37), some have also expressed concerns that the process of user fee removal must be designed carefully such that facilities are able to adapt. The elimination of fees and reduction of fees without a corresponding increase in resources from government sources could unintentionally lead to increased pressure on human resources and the availability of essential commodities, including vaccines. Further, increased demand for curative services without an increase in the health workforce could result in curative services "crowding out" preventative services such as vaccination. Ultimately, further research into any potentially paradoxical relationship between user fees and DPT3 vaccination.

The study should also be interpreted in light of its limitations. As the study is observational and cross-sectional, the association presented should not be interpreted as causal effects. Using data aggregated at the subnational level also entails the potential for an ecological fallacy and requires caution in interpretation. In addition, while the sample was constructed such that the time between the SPA and DHS surveys in each country was less than 5 years, it is possible that changes in time-varying factors or other events in that interlude could influence the outcomes under study. Afghanistan's more limited sample size restricted to predominantly urban areas presents an additional limitation, therefore it was excluded from regression analysis to mitigate any potential confounding. Finally, though we control for selected characteristics at the subnational and individual levels, there is a potential for omitted variable bias through other factors that may influence the capacity, quality, and performance variables under study but are unmeasured in the SPA and DHS surveys. Ultimately, further research into the relationships between PHC capacity, quality and service delivery outcomes could adopt other methodologies to establish potential causal links. Taking advantage of repeated survey waves may provide one such strategy to track how these indicators may have changed over time and to what extent improvements in capacity and quality lead to better results.

Conclusion

Substantial heterogeneity and disparities were observed across the 12 studied LMICS, as well as between and within subnational areas in both the capacity and quality subdomains. Countries seeking to strengthen their PHC systems could improve PHC monitoring at the subnational level to better understand the diverse challenges across subnational areas and develop tailored approaches to resolve

For peer review only - http://bmjopen.bmj.com/site/about/guidelines.xhtml

the problems identified. The associations observed between capacity, quality and performance also provide potential avenues for further research and contribute to the literature on the determinants of care-seeking behavior and immunization.

Ethics statements

Data included in the present analysis were extracted from anonymized publicly available datasets and no patient consent was needed

Availability of data and material

The original datasets analyzed during the current study are available from the DHS program website, https://dhsprogram.com/data/available-datasets.cfm. The analytical dataset generated during the current study is available from the corresponding author on reasonable request

Authors Contributions: MR and MVU were responsible for the conception of this study and for methods development. MR analyzed data. MR, JCG, CF prepared the first draft. All authors reviewed results or reviewed and contributed to the final manuscript.

Funding: This research was supported, in whole or in part, by the Bill and Melinda Gates Foundation (Grant number INV000932), and it had no role in study design, data collection, data analysis, data interpretation or writing of the manuscript.

Competing interests: None declared.

Reference

1. World Health Organization, Organisation for Economic Co-operation and Development, International Bank for Reconstruction and Development. Delivering quality health services: a global imperative for universal health coverage [Internet]. Geneva: World Health Organization; 2018. Available from: https://apps.who.int/iris/handle/10665/272465

2. Bitton A, Fifield J, Ratcliffe H, Karlage A, Wang H, Veillard JH, et al. Primary healthcare system performance in low-income and middle-income countries: a scoping review of the evidence from 2010 to 2017. BMJ Glob Health. 2019 Aug 16;4(Suppl 8):e001551.

3. Vital Signs Profiles | PHCPI [Internet]. [cited 2022 May 18]. Available from: https://improvingphc.org/vital-signs-profiles

4. Randhawa H. Report of the Expert Consultation on Primary Care Systems Profiles & Performance (PRIMASYS). :35.

5. Veillard J, Cowling K, Bitton A, Ratcliffe H, Kimball M, Barkley S, et al. Better Measurement for Performance Improvement in Low- and Middle-Income Countries: The Primary Health Care Performance Initiative (PHCPI) Experience of Conceptual Framework Development and Indicator Selection. Milbank Q. 2017 Dec;95(4):836–83.

6. Tanahashi T. Health service coverage and its evaluation. Bull World Health Organ. 1978;56(2):295–303.

For peer review only - http://bmjopen.bmj.com/site/about/guidelines.xhtml

7. Kruk ME, Gage AD, Arsenault C, Jordan K, Leslie HH, Roder-DeWan S, et al. High-quality health systems in the Sustainable Development Goals era: time for a revolution. Lancet Glob Health. 2018 Nov;6(11):e1196–252.

8. OECD, Organization WH, Group WB. Delivering Quality Health Services: A Global Imperative [Internet]. 2018. 96 p. Available from: https://www.oecd-ilibrary.org/content/publication/9789264300309-en

9. National Academies of Sciences E and Medicine. Crossing the Global Quality Chasm: Improving Health Care Worldwide [Internet]. Washington, DC: The National Academies Press; 2018 [cited 2022 Mar 14]. 334 p. Available from: https://www.nap.edu/catalog/25152/crossing-the-global-quality-chasm-improving-health-care-worldwide

10. Ng M, Fullman N, Dieleman JL, Flaxman AD, Murray CJL, Lim SS. Effective Coverage: A Metric for Monitoring Universal Health Coverage. PLoS Med. 2014 Sep 22;11(9):e1001730.

11. Nikoloski Z, Albala S, Montero AM, Mossialos E. The impact of primary health care and specialist physician supply on amenable mortality in Mexico (2000-2015): Panel data analysis using system-Generalized Method of Moments. Soc Sci Med. 2021 Jun;278:113937.

12. Schneider MT, Chang AY, Crosby SW, Gloyd S, Harle AC, Lim S, et al. Trends and outcomes in primary health care expenditures in low-income and middle-income countries, 2000–2017. BMJ Glob Health. 2021 Aug;6(8):e005798.

13. Basu S, Berkowitz SA, Phillips RL, Bitton A, Landon BE, Phillips RS. Association of Primary Care Physician Supply With Population Mortality in the United States, 2005-2015. JAMA Intern Med. 2019 Apr 1;179(4):506–14.

14. Hone T, Rasella D, Barreto M, Atun R, Majeed A, Millett C. Large Reductions In Amenable Mortality Associated With Brazil's Primary Care Expansion And Strong Health Governance. Health Aff (Millwood). 2017 Jan 1;36(1):149–58.

15. Russo LX, Scott A, Sivey P, Dias J. Primary care physicians and infant mortality: Evidence from Brazil. PLOS ONE. 2019 May 31;14(5):e0217614.

16. Ranabhat CL, Jakovljevic M, Dhimal M, Kim CB. Structural Factors Responsible for Universal Health Coverage in Low- and Middle-Income Countries: Results From 118 Countries. Frontiers in Public Health [Internet]. 2020 [cited 2022 Mar 14];7. Available from: https://www.frontiersin.org/article/10.3389/fpubh.2019.00414

17. Lozano R, Fullman N, Mumford JE, Knight M, Barthelemy CM, Abbafati C, et al. Measuring universal health coverage based on an index of effective coverage of health services in 204 countries and territories, 1990–2019: a systematic analysis for the Global Burden of Disease Study 2019. The Lancet. 2020 Oct 17;396(10258):1250–84.

18. Leslie HH, Malata A, Ndiaye Y, Kruk ME. Effective coverage of primary care services in eight high-mortality countries. BMJ Glob Health. 2017 Sep 4;2(3):e000424.

19. Nguhiu PK, Barasa EW, Chuma J. Determining the effective coverage of maternal and child health services in Kenya, using demographic and health survey data sets: tracking progress towards universal health coverage. Trop Med Int Health. 2017 Apr;22(4):442–53.

For peer review only - http://bmjopen.bmj.com/site/about/guidelines.xhtml

20. USAID. The DHS Program - Service Provision Assessments (SPA) [Internet]. Demographic Health Surveys. 2019 [cited 2020 Nov 28]. p. 1–2. Available from: https://dhsprogram.com/What-We-Do/Survey-Types/SPA.cfm

21. Croft TN, Marshall AM, Allen CK, Arnold F, Assaf S, Balian S. Guide to DHS statistics. Rockville: ICF. 2018;645.

22. Wulifan JK, Brenner S, Jahn A, De Allegri M. A scoping review on determinants of unmet need for family planning among women of reproductive age in low and middle income countries. BMC Womens Health. 2016 Jan 15;16:2.

23. Bailey C, Blake C, Schriver M, Cubaka VK, Thomas T, Martin Hilber A. A systematic review of supportive supervision as a strategy to improve primary healthcare services in Sub-Saharan Africa. International Journal of Gynecology & Obstetrics. 2016 Jan 1;132(1):117–25.

24. Rao KD, Sheffel A. Quality of clinical care and bypassing of primary health centers in India. Soc Sci Med. 2018 Jun;207:80–8.

25. Gauthier B, Wane W. Bypassing health providers: The quest for better price and quality of health care in Chad. Social Science & Medicine. 2011 Aug 1;73(4):540–9.

26. Kruk ME, Hermosilla S, Larson E, Mbaruku GM. Bypassing primary care clinics for childbirth: a cross-sectional study in the Pwani region, United Republic of Tanzania. Bull World Health Organ. 2014 Apr 1;92(4):246–53.

27. Alqossayir FM, Alkhowailed MS, Alammar AY, Alsaeed AA, Alamri YY, Rasheed Z. Factors associated with patients bypassing primary healthcare centres in Qassim Region, KSA. J Taibah Univ Med Sci. 2021 Dec;16(6):900–5.

28. Audo MO, Ferguson A, Njoroge PK. Quality of health care and its effects in the utilisation of maternal and child health services in Kenya. East Afr Med J. 2005 Nov;82(11):547–53.

29. Colvin CJ, Smith HJ, Swartz A, Ahs JW, de Heer J, Opiyo N, et al. Understanding careseeking for child illness in sub-Saharan Africa: a systematic review and conceptual framework based on qualitative research of household recognition and response to child diarrhoea, pneumonia and malaria. Soc Sci Med. 2013 Jun;86:66–78.

30. Bell SK, Delbanco T, Elmore JG, Fitzgerald PS, Fossa A, Harcourt K, et al. Frequency and Types of Patient-Reported Errors in Electronic Health Record Ambulatory Care Notes. JAMA Netw Open. 2020 Jun 1;3(6):e205867.

31. Geldsetzer P, Williams TC, Kirolos A, Mitchell S, Ratcliffe LA, Kohli-Lynch MK, et al. The Recognition of and Care Seeking Behaviour for Childhood Illness in Developing Countries: A Systematic Review. PLOS ONE. 2014 Apr 9;9(4):e93427.

32. Bruce N, Pope D, Arana B, Shiels C, Romero C, Klein R, et al. Determinants of Care Seeking for Children With Pneumonia and Diarrhea in Guatemala: Implications for Intervention Strategies. Am J Public Health. 2014 Apr;104(4):647–57.

33. Gage AD, Leslie HH, Bitton A, Jerome JG, Joseph JP, Thermidor R, et al. Does quality influence utilization of primary health care? Evidence from Haiti. Globalization and Health. 2018 Jun 20;14(1):59.

For peer review only - http://bmjopen.bmj.com/site/about/guidelines.xhtml

34. Ghosh A, Laxminarayan R. Demand- and supply-side determinants of diphtheria-pertussis-tetanus nonvaccination and dropout in rural India. Vaccine. 2017 Feb 15;35(7):1087–93.

35. Watson SI, Wroe EB, Dunbar EL, Mukherjee J, Squire SB, Nazimera L, et al. The impact of user fees on health services utilization and infectious disease diagnoses in Neno District, Malawi: a longitudinal, quasi-experimental study. BMC Health Services Research. 2016 Oct 20;16(1):595.

36. Nguyen HT, Zombré D, Ridde V, De Allegri M. The impact of reducing and eliminating user fees on facility-based delivery: a controlled interrupted time series in Burkina Faso. Health Policy and Planning. 2018 Oct 1;33(8):948–56.

37. Lagarde M, Barroy H, Palmer N. Assessing the effects of removing user fees in Zambia and Niger. J Health Serv Res Policy. 2012 Jan;17(1):30–6.

For peer review only

Supplementary Methods

Supplementary Table 1: Characteristics of DHS and SPA surveys included in the analysis

Country	Sub-national areas	DHS Field Work	DHS sampling	DHS sample size (ever-married Women)	Year data collection -SPA	SPA sample Size (Facilities)
**Afghanistan**	34 provinces	June 2015 - February 2016	two-stage stratified sample	24,395	November 2018- January 2019	160 facilities**
**Bangladesh**	8 divisions	October 24, 2017- March 2018	two-stage stratified sample	20,100	July 2017-to October 2017	1,600
**DRC**	26 new provinces	August 2013 - February 2014	Three-stage stratified sample*	18,827	October 2017 - April 2018	1380
**Haiti**	10 departments	November 2016 - April 2017	two-stage stratified sample	14,371	December 2017 - May 2018	1007
**Kenya**	8 regions	May 2014 - October 2014	two-stage stratified sample	31,079	January 2010- May 2010	695
**Malawi**	3 regions	October 2015 - February 2016	two-stage stratified sample	24562	June 2013- February 2014	1060
**Namibia**	13 regions	May 2013 - September 2013	two-stage stratified sample	9176	July 2009- October 2009	446
**Nepal**	7 provinces	June 2016 - January 2017	Stratified sample (two stages in rural areas and three stages in urban areas)	12862	April 2015- November 2015	1,000
**Rwanda**	5 provinces	September 2010 - March 2011	two-stage stratified sample	13671	June 2007- August 2007	538
**Senegal**	14 regions	April 2017 - December 2017	two-stage stratified sample	16787	March 2017- December 2017	3764
**Tanzania**	25 regions	August 2015 - February 2016	two-stage stratified sample	13266	October 2014 – March 2015	1,200
**Uganda**	15 regions	June 2011 - December 2011	two-stage stratified sample	8674	July-October 2007	491

*Two-stage stratified sample in statutory towns and cities of established provinces, three-stage stratified sample in rest of established provinces and new provinces, **Sampling limited to urban areas of seven major provinces (Kabul, Nangarhar, Paktya, Kunduz, Balkh, Kandahar, and Herat)

For peer review only

Supplementary Table 2: Definition of capacity, quality and output indicators utilized in the analysis

Domain	Subdomain	Indicator	Definition	Data Source
**Capacity**	Facility Infrastructure	SRI for basic amenities and infection prevention	Service readiness index for basic amenities and infection prevention	SPA
	Recent Supervision	Supervision within the past 6 months	Proportion of providers within a facility with supervision in past 6 months	SPA
	Supervisor Feedback	Supervisor provided verbal or written feedback	Proportion of providers within a facility provided verbal or written supervision feedback	SPA
	Outreach Services	Facility provides outreach services	Facility provides sick child, growth monitoring or immunization outreach service	SPA
	Not charging PHC Fees	Facility does not charge PHC fees	Facility does not charge fees for PHC services (fees for a specific PHC service or a fixed fee that covers all services that a client receives)	SPA
**Quality**	Waiting Time < 60 minutes	Wait time less than one hour	Proportion of observed visits with wait times less than one hour	SPA
	Provider Availability	Proportion of visits over 10 minutes	The proportion of ANC, FP, LD and sick child visits that last for more than 10 minutes.	SPA
	Provider Competence	Antenatal care quality score based on WHO guidelines	Average quality score for observed ANC visits based on WHO ANC guidelines	SPA
		Family planning quality score based on WHO guidelines	Average quality score for observed family visits based on WHO family planning guidelines	
		Sick child quality score based on IMCI guidelines	Average quality score for observed sick child visits based on WHO Integrated Management of Childhood Illness (IMCI) guidelines	
	Person Centeredness	Proportion of caregivers who were told the sick child diagnosis	Proportion of observed sick child visits where the health worker told the child's caretaker what illness(es) the child has	SPA
	Safety	Proportion of rooms with all infection control items	Proportion of rooms (FP, sick child, ANC and NCD) where all infection control tracer items are present	SPA
		Adequate waste disposal	Average score (out of 3) on adherence to standards for disposing of medical and hazardous waste, sharps, and having guidelines for waste disposal in place.	
	Comprehensiveness	Proportion of 3 NCD services offering diagnosis and treatment (diabetes, CRD, CVD)	Proportion of non-combinable disease services provided and for which guidelines are available (diabetes, chronic respiratory disease, and chronic cardiovascular disease) across all facilities	SPA
		Proportion of 3 primary ID services provided (STI, TB, HIV)	Proportion of infectious diseases services provided and for which guidelines are available for sexually transmitted infections, tuberculosis, and HIV across all facilities.	SPA
		Average availability of 5 tracer RMNCH services (SC, Vacc, FP, ANC, PMTCT)	Proportion of maternal and child health services provided and for which guidelines are available (sick child, vaccination, family planning, antenatal care, and prevention of mother-to-child transmission of HIV) across all facilities.	SPA
**Outputs**	Maternal	Antenatal care coverage - at least four visits (%)	Antenatal care coverage (4+) visits is the percent of women with a live birth who received antenatal care (ANC) 4 or more times.	DHS
		Demand for family planning satisfied with modern methods (%)	Proportion of married or in-union women of reproductive age (aged 15-49 years) who are married or in a union and have their need for family planning satisfied with modern methods.	DHS
		Perceived barriers due to treatment costs	Percentage of women who report specific problems in accessing health care when they are sick due to issues related to getting money for treatment	DHS
		Perceived barriers due to distance	Percentage of women who report specific problems in accessing health care when they are sick due to distance that they have to travel for treatment	DHS
	Child	Coverage of DTP3 immunization	Diphtheria-tetanus-pertussis (DTP) coverage measures the percent of one-year-olds who have received three doses of the combined diphtheria, tetanus toxoid and pertussis vaccine in a given year.	DHS
		DPT Dropout rate	Percent of children who do not receive three doses of DTP after receiving an initial dose. Calculated as (DPT1-DPT3/DPT1)	DHS
		Children aged < 5 years with pneumonia symptoms taken to a healthcare provider (%)	Percentage of children under 5 years of age with suspected pneumonia (cough and difficulty breathing NOT due to a problem in the chest and a blocked nose) in the two weeks preceding the survey taken to an appropriate health facility or provider.	DHS
		Children aged < 5 years with diarrhea receiving oral rehydration salts	The percent of children with diarrhea, a leading cause of death in children under five, who received appropriate treatment with oral rehydration solution.	DHS

For peer review only

Supplementary Figure 1: Primary Healthcare Performance Initiative (PHCPI) Framework

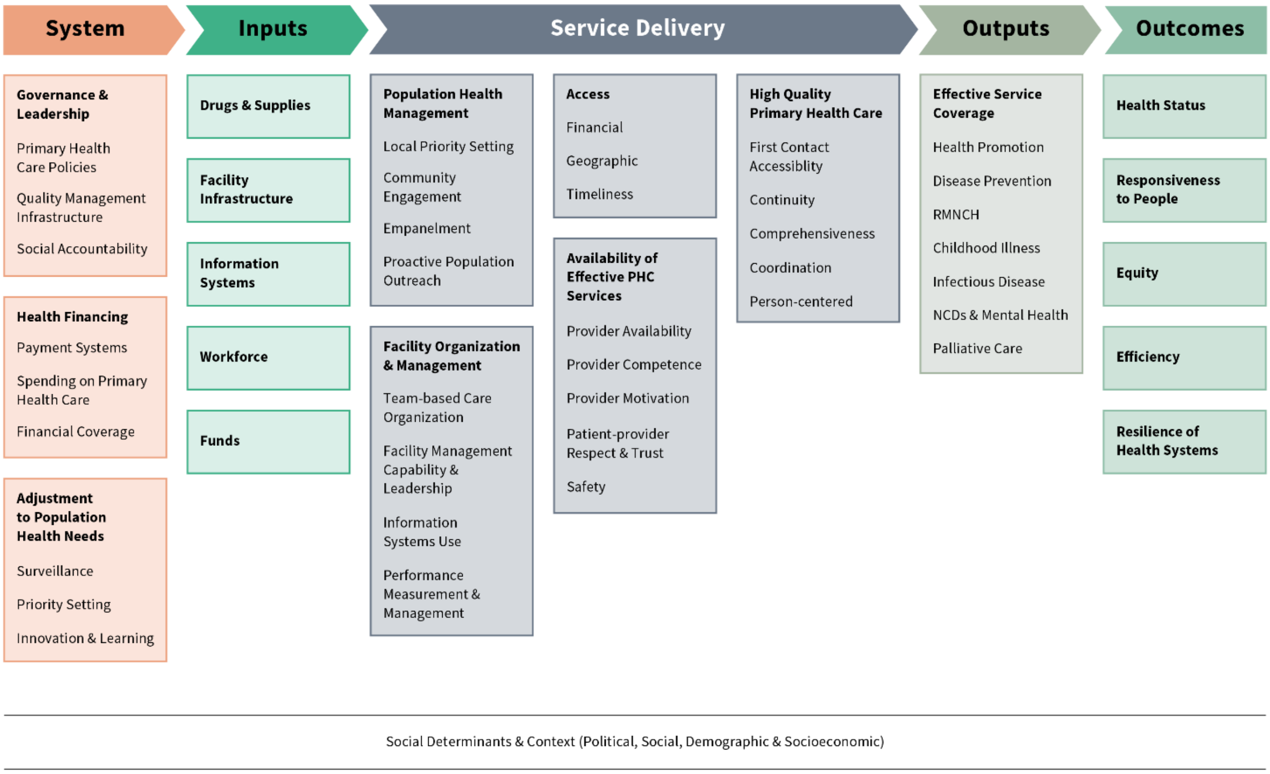

ew only

Supplementary Figure 2: Linkage of SPA and DHS datasets

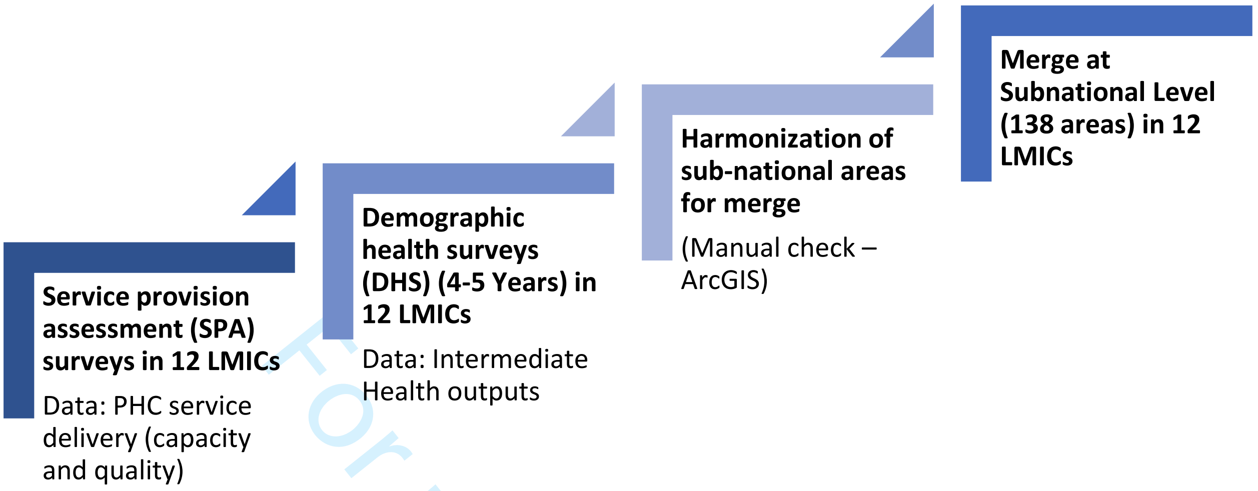

12 Countries: Afghanistan, Bangladesh, DRC, Haiti, Kenya, Malawi, Namibia, Nepal, Rwanda, Senegal, Tanzania, Uganda)

For peer review only

Supplementary Results

Supplementary Table 3: Capacity of PHC service delivery at the subnational level in the studied countries

Country	Division	Facility Infrastructure	Recent Supervision	Supervisor Feedback	Outreach Services	Not Charging PHC Fees	Average Capacity score	Range across capacity sub-domains*
Afghanistan	Balkh	83%	85%	63%	7%	56%	59%	78%
	Herat	85%	59%	29%	4%	71%	50%	81%
	Kabul	86%	63%	54%	31%	36%	54%	55%
	Kandahar	82%	99%	73%	29%	45%	66%	70%
	Kunduz	76%	91%	76%	0%	53%	59%	91%
	Nangarhar	75%	70%	63%	0%	45%	51%	75%
	Paktya	74%	70%	44%	25%	43%	51%	49%
Bangladesh	Barisal	49%	95%	93%	14%	67%	64%	81%
	Chittagong	51%	91%	85%	20%	80%	65%	71%
	Dhaka	52%	92%	89%	26%	54%	63%	66%
	Khulna	54%	97%	92%	21%	54%	64%	76%
	Mymensingh	46%	99%	96%	14%	45%	60%	85%
	Rajshahi	55%	96%	94%	19%	81%	69%	77%
	Rangpur	51%	97%	96%	14%	76%	67%	83%
	Sylhet	48%	89%	87%	18%	78%	64%	71%
DRC	Bas-uele	37%	68%	66%	91%	73%	67%	54%
	Equateur	42%	80%	71%	95%	24%	62%	71%
	Haut-katanga	45%	73%	63%	73%	46%	60%	28%
	Haut-lomami	53%	57%	49%	29%	56%	49%	28%
	Haut-uele	42%	75%	68%	81%	44%	62%	39%
	Ituri	51%	79%	74%	82%	30%	63%	52%
	Kasai	43%	72%	63%	25%	34%	47%	47%
	Kasai-central	49%	86%	86%	84%	40%	69%	46%
	Kasai-oriental	43%	83%	79%	74%	59%	67%	40%
	Kinshasa	60%	64%	56%	33%	66%	56%	33%
	Kongo central	55%	58%	54%	40%	58%	53%	18%
	Kwango	48%	91%	86%	95%	16%	67%	79%
	Kwilu	48%	71%	64%	90%	20%	58%	70%
	Lomami	47%	91%	91%	86%	37%	71%	54%
	Lualaba	54%	77%	76%	54%	65%	65%	23%

	Mai-ndombe	47%	85%	77%	92%	36%	67%	56%
	Maniema	57%	89%	85%	65%	65%	72%	32%
	Mongala	40%	87%	65%	78%	39%	62%	48%
	Nord-kivu	43%	91%	84%	94%	0%	63%	94%
	Nord-ubangi	55%	83%	82%	64%	34%	64%	49%
	Sankuru	36%	64%	56%	83%	81%	64%	47%
	Sud-kivu	49%	88%	85%	86%	0%	62%	88%
	Sud-ubangi	60%	75%	71%	69%	16%	58%	59%
	Tanganyka	47%	72%	71%	75%	64%	66%	28%
	Tshopo	41%	71%	67%	67%	52%	60%	30%
	Tshuapa	44%	84%	81%	94%	36%	68%	58%
**Haiti**	Artibonite	60%	78%	70%	67%	62%	67%	18%
	Centre	58%	90%	79%	70%	72%	74%	32%
	Grand'anse	66%	84%	76%	83%	78%	77%	18%
	Nippes	67%	64%	52%	29%	58%	54%	38%
	Nord	68%	73%	56%	42%	68%	61%	31%
	Nord-est	61%	66%	60%	45%	64%	59%	21%
	Nord-ouest	63%	58%	56%	4%	84%	53%	80%
	Ouest	59%	72%	63%	13%	76%	57%	63%
	Sud	56%	75%	49%	20%	98%	60%	78%
	Sud-est	63%	83%	75%	22%	68%	62%	61%
**Kenya**	Central	69%	76%	66%	64%	46%	64%	30%
	Coast	69%	71%	65%	53%	70%	66%	18%
	Eastern	71%	70%	60%	46%	64%	62%	25%
	Nairobi	61%	81%	72%	71%	72%	71%	20%
	North eastern	61%	83%	70%	42%	63%	64%	41%
	Nyanza	62%	69%	52%	31%	92%	61%	61%
	Rift valley	72%	67%	60%	14%	66%	56%	58%
	Western	61%	73%	62%	20%	63%	56%	53%
**Malawi**	Central region	68%	73%	64%	67%	67%	68%	9%
	Northern region	65%	75%	67%	78%	82%	73%	17%
	Southern region	69%	74%	65%	64%	72%	69%	10%
**Namibia**	Caprivi	58%	77%	49%	3%	97%	57%	94%
	Erongo	73%	63%	58%	18%	95%	61%	77%
	Hardap	78%	78%	69%	17%	83%	65%	66%
	Karas	72%	73%	68%	15%	92%	64%	77%
	Kavango	61%	71%	53%	21%	97%	60%	76%
	Khomas	74%	56%	50%	0%	75%	51%	75%
	Kunene	63%	69%	56%	14%	93%	59%	79%

	Ohangwena	66%	83%	68%	21%	100%	68%	79%
	Omaheke	75%	84%	82%	25%	100%	73%	75%
	Omusati	72%	79%	68%	42%	100%	72%	58%
	Oshana	68%	78%	66%	43%	100%	71%	57%
	Oshikoto	70%	70%	57%	18%	95%	62%	77%
	Otjozondjupa	74%	52%	47%	20%	97%	58%	77%
**Nepal**	Central	54%	71%	61%	73%	92%	70%	38%
	Eastern	54%	66%	60%	86%	93%	72%	39%
	Far-western	52%	76%	67%	88%	90%	75%	38%
	Mid-western	53%	71%	64%	88%	90%	73%	37%
	Western	54%	74%	69%	87%	94%	76%	40%
**Rowanda**	East	56%	91%	79%	81%	97%	81%	41%
	Kigali city	70%	56%	48%	69%	92%	67%	44%
	North	60%	84%	75%	80%	100%	80%	40%
	South	64%	91%	86%	87%	93%	84%	29%
	West	62%	90%	83%	77%	98%	82%	36%
**Senegal**	Dakar	79%	64%	62%	22%	67%	59%	57%
	Diourbel	83%	81%	80%	64%	60%	74%	23%
	Fatick	66%	91%	89%	37%	57%	68%	54%
	Kaffrine	47%	86%	86%	45%	72%	67%	41%
	Kaolack	51%	68%	67%	16%	66%	54%	52%
	Kedougou	45%	78%	78%	58%	66%	65%	33%
	Kolda	45%	86%	81%	61%	69%	69%	41%
	Louga	61%	58%	57%	53%	65%	59%	12%
	Matam	63%	73%	70%	58%	66%	66%	15%
	Saint-louis	62%	74%	74%	56%	60%	65%	18%
	Sedhiou	68%	100%	98%	51%	61%	75%	49%
	Tambacounda	61%	69%	66%	77%	66%	68%	16%
	This	61%	69%	68%	46%	67%	62%	23%
	Ziguinchor	70%	87%	86%	35%	64%	68%	52%
**Tanzania**	Arusha	62%	77%	68%	19%	14%	48%	63%
	Dar es salaam	49%	68%	67%	47%	11%	48%	57%
	Dodoma	73%	59%	55%	15%	52%	51%	58%
	Geita	54%	57%	54%	57%	17%	48%	40%
	Iringa	57%	58%	57%	58%	23%	51%	35%
	Kagera	55%	47%	41%	63%	15%	44%	48%
	Kaskazini pemba	59%	85%	78%	76%	29%	65%	56%
	Kaskazini unguja	62%	78%	74%	50%	86%	70%	36%

	Katavi	66%	83%	81%	40%	84%	71%	44%
	Kigoma	49%	80%	70%	82%	14%	59%	68%
	Kilimanjaro	52%	79%	66%	26%	25%	50%	54%
	Kusini pemba	61%	75%	66%	34%	41%	55%	41%
	Kusini unguja	61%	68%	64%	59%	86%	68%	27%
	Lindi	64%	78%	77%	15%	81%	63%	66%
	Manyara	52%	86%	77%	38%	26%	56%	60%
	Mara	53%	86%	78%	37%	35%	58%	51%
	Mbeya	54%	74%	70%	47%	22%	53%	52%
	Mjini magharibi	55%	59%	56%	59%	22%	50%	37%
	Morogoro	57%	65%	62%	56%	25%	53%	40%
	Mtwara	66%	45%	43%	16%	48%	44%	50%
	Mwanza	60%	48%	40%	73%	21%	49%	52%
	Njombe	51%	66%	56%	40%	13%	45%	53%
	Pwani	53%	54%	53%	51%	23%	47%	31%
	Rukwa	49%	44%	40%	54%	4%	38%	50%
	Ruvuma	51%	45%	36%	67%	28%	45%	39%
	Shinyanga	49%	95%	92%	70%	31%	67%	64%
	Simiyu	53%	79%	72%	64%	12%	56%	67%
	Singida	54%	90%	89%	75%	32%	68%	58%
	Tabora	51%	75%	74%	66%	15%	56%	60%
	Tanga	50%	90%	90%	38%	18%	57%	72%
**Uganda**	Central 1	52%	91%	78%	83%	100%	81%	48%
	Central 2	44%	91%	70%	77%	97%	76%	53%
	East central	55%	82%	69%	85%	100%	78%	45%
	Eastern	70%	60%	49%	57%	93%	66%	44%
	Kampala	54%	85%	68%	85%	100%	78%	46%
	North	45%	79%	34%	84%	100%	68%	66%
	Southwest	55%	82%	59%	82%	100%	76%	45%
	West-nile	55%	69%	57%	67%	100%	70%	45%
	Western	64%	93%	72%	94%	100%	85%	36%

Supplementary Table 4: Quality of PHC service delivery at the subnational level in the studied countries

Country	Division	Waiting Time	Provider Availability	Provider Competence	Person Centeredness	Safety	Comprehensive-ness	Average Quality score	Range across quality Subdomains *
Afghanistan	Balkh	89%	35%	28%	75%	28%	65%	53%	61%
	Herat	84%	34%	34%	50%	33%	52%	48%	51%
	Kabul	91%	20%	24%	34%	52%	67%	48%	71%
	Kandahar	100%	34%	19%	1%	55%	75%	47%	99%
	Kunduz	87%	42%	38%	89%	6%	69%	55%	83%
	Nangarhar	97%	48%	34%	45%	17%	78%	53%	80%
	Paktya	94%	54%	30%	22%	23%	73%	49%	72%
Bangladesh	Barisal	-	-	-	-	39%	67%	53%	28%
	Chittagong	-	-	-	-	39%	64%	52%	25%
	Dhaka	-	-	-	-	40%	61%	50%	21%
	Khulna	-	-	-	-	39%	64%	52%	25%
	Mymensingh	-	-	-	-	31%	65%	48%	34%
	Rajshahi	-	-	-	-	44%	68%	56%	24%
	Rangpur	-	-	-	-	45%	50%	47%	5%
	Sylhet	-	-	-	-	33%	68%	51%	35%
DRC	Bas-uele	74%	68%	39%	45%	35%	70%	55%	39%
	Equateur	90%	86%	36%	46%	28%	74%	60%	62%
	Haut-katanga	82%	89%	45%	40%	22%	86%	61%	67%
	Haut-lomami	78%	65%	43%	22%	45%	87%	57%	65%
	Haut-uele	63%	64%	34%	20%	26%	70%	46%	50%
	Ituri	89%	75%	39%	24%	39%	56%	54%	65%
	Kasai	95%	82%	30%	8%	19%	53%	48%	87%
	Kasai-central	73%	78%	39%	45%	30%	68%	56%	48%
	Kasai-oriental	69%	72%	38%	39%	28%	79%	54%	51%
	Kinshasa	44%	69%	48%	22%	29%	79%	48%	57%
	Kongo central	83%	88%	44%	33%	34%	88%	62%	55%
	Kwango	87%	87%	40%	11%	28%	71%	54%	76%

For peer review only - http://bmjopen.bmj.com/site/about/guidelines.xhtml

	Kwilu	65%	67%	37%	12%	37%	87%	51%	75%
	Lomami	70%	66%	37%	28%	36%	91%	55%	63%
	Lualaba	65%	63%	47%	40%	34%	91%	56%	57%
	Mai-ndombe	75%	86%	38%	23%	27%	78%	55%	63%
	Maniema	78%	71%	37%	46%	42%	94%	61%	57%
	Mongala	85%	89%	36%	50%	21%	71%	59%	68%
	Nord-kivu	65%	78%	42%	47%	30%	58%	54%	48%
	Nord-ubangi	77%	76%	48%	9%	49%	79%	56%	70%
	Sankuru	88%	87%	35%	30%	27%	49%	53%	61%
	Sud-kivu	75%	65%	40%	33%	36%	81%	55%	48%
	Sud-ubangi	71%	82%	44%	49%	43%	68%	60%	39%
	Tanganyka	80%	87%	33%	31%	42%	52%	54%	56%
	Tshopo	81%	88%	37%	20%	27%	71%	54%	68%
	Tshuapa	82%	88%	38%	41%	30%	69%	58%	58%
**Haiti**	Artibonite	50%	62%	36%	15%	51%	79%	49%	64%
	Centre	66%	70%	33%	7%	56%	88%	53%	81%
	Grand'anse	48%	71%	37%	8%	61%	85%	51%	77%
	Nippes	42%	59%	34%	18%	45%	75%	45%	57%
	Nord	52%	67%	36%	13%	55%	83%	51%	70%
	Nord-est	46%	68%	39%	10%	53%	77%	49%	67%
	Nord-ouest	64%	67%	49%	34%	76%	70%	60%	42%
	Ouest	70%	57%	44%	48%	51%	78%	58%	34%
	Sud	93%	54%	37%	44%	45%	64%	56%	56%
	Sud-est	53%	72%	58%	58%	60%	89%	65%	36%
**Kenya**	Central	30%	60%	36%	9%	55%	81%	45%	72%
	Coast	49%	63%	35%	14%	70%	90%	54%	76%
	Eastern	54%	63%	35%	15%	58%	83%	51%	68%
	Nairobi	53%	72%	32%	20%	56%	83%	52%	63%
	North eastern	73%	59%	44%	39%	66%	89%	62%	50%
	Nyanza	84%	34%	46%	71%	78%	72%	64%	50%
	Rift valley	65%	77%	50%	70%	71%	71%	67%	27%
	Western	75%	75%	51%	68%	67%	76%	69%	25%

**Malawi**	Central region	49%	43%	38%	51%	50%	75%	51%	37%
	Northern region	46%	42%	37%	41%	46%	77%	48%	40%
	Southern region	49%	39%	37%	43%	51%	72%	48%	35%
**Namibia**	Caprivi	30%	70%	52%	29%	81%	93%	59%	64%
	Erongo	59%	49%	52%	76%	65%	77%	63%	28%
	Hardap	49%	69%	51%	45%	86%	84%	64%	41%
	Karas	65%	59%	48%	45%	78%	87%	64%	42%
	Kavango	34%	48%	49%	30%	75%	94%	55%	64%
	Khomas	49%	69%	51%	84%	86%	55%	66%	37%
	Kunene	73%	76%	59%	79%	78%	86%	75%	27%
	Ohangwena	8%	55%	59%	63%	70%	93%	58%	85%
	Omaheke	58%	79%	67%	79%	91%	90%	77%	33%
	Omusati	39%	59%	49%	35%	69%	92%	57%	57%
	Oshana	25%	62%	60%	65%	59%	84%	59%	59%
	Oshikoto	34%	74%	64%	53%	54%	95%	62%	61%
	Otjozondjupa	46%	48%	47%	26%	61%	79%	51%	53%
**Nepal**	Central	96%	31%	26%	27%	42%	76%	50%	70%
	Eastern	90%	47%	31%	40%	44%	76%	55%	59%
	Far-western	94%	58%	34%	37%	42%	79%	57%	60%
	Mid-western	91%	45%	32%	27%	49%	74%	53%	64%
	Western	96%	41%	33%	37%	49%	75%	55%	63%
**Rowanda**	East	31%	41%	40%	13%	21%	79%	37%	66%
	Kigali city	52%	51%	45%	51%	31%	57%	48%	26%
	North	17%	34%	41%	27%	24%	76%	37%	59%
	South	43%	33%	35%	19%	32%	83%	41%	64%
	West	42%	37%	44%	18%	24%	78%	40%	60%
**Senegal**	Dakar	71%	63%	42%	24%	67%	70%	56%	47%
	Diourbel	99%	71%	42%	10%	81%	77%	63%	89%
	Fatick	94%	70%	42%	10%	72%	58%	58%	84%
	Kaffrine	71%	61%	47%	4%	53%	52%	48%	67%
	Kaolack	78%	69%	48%	29%	58%	53%	56%	49%

	Kedougou	86%	77%	49%	8%	45%	56%	53%	78%
	Kolda	76%	71%	44%	6%	49%	51%	50%	70%
	Louga	63%	49%	34%	1%	59%	74%	47%	73%
	Matam	72%	24%	28%	1%	75%	74%	46%	74%
	Saint-louis	43%	49%	33%	2%	68%	73%	45%	71%
	Sedhiou	91%	72%	38%	1%	79%	64%	58%	90%
	Tambacounda	62%	59%	32%	0%	69%	81%	50%	81%
	This	74%	60%	43%	20%	61%	64%	54%	54%
	Ziguinchor	97%	74%	37%	12%	74%	66%	60%	85%
**Tanzania**	Arusha	74%	76%	45%	44%	63%	59%	60%	32%
	Dar es salaam	59%	82%	42%	68%	35%	72%	60%	47%
	Dodoma	76%	76%	40%	44%	52%	56%	57%	36%
	Geita	71%	87%	35%	13%	26%	71%	51%	74%
	Iringa	42%	60%	44%	78%	30%	75%	54%	48%
	Kagera	76%	70%	45%	52%	37%	74%	59%	39%
	Kaskazini pemba	28%	72%	46%	77%	35%	76%	56%	49%
	Kaskazini unguja	71%	74%	39%	19%	27%	76%	51%	57%
	Katavi	22%	81%	47%	65%	66%	87%	61%	65%
	Kigoma	46%	74%	35%	67%	31%	83%	56%	52%
	Kilimanjaro	28%	56%	40%	41%	24%	83%	45%	59%
	Kusini pemba	84%	82%	35%	33%	54%	68%	59%	51%
	Kusini unguja	67%	82%	38%	23%	25%	74%	51%	59%
	Lindi	58%	77%	44%	67%	58%	89%	65%	45%
	Manyara	66%	61%	34%	57%	42%	63%	54%	32%
	Mara	54%	79%	48%	27%	47%	57%	52%	52%
	Mbeya	69%	74%	33%	24%	36%	61%	49%	50%
	Mjini magharibi	68%	59%	35%	46%	50%	78%	56%	43%
	Morogoro	61%	50%	36%	16%	61%	70%	49%	54%
	Mtwara	72%	57%	33%	50%	41%	66%	53%	39%
	Mwanza	41%	68%	38%	54%	44%	71%	53%	33%
	Njombe	55%	65%	43%	78%	36%	68%	58%	42%

	Pwani	41%	71%	47%	44%	36%	70%	52%	35%
	Rukwa	81%	76%	45%	27%	40%	60%	55%	54%
	Ruvuma	62%	43%	29%	19%	36%	75%	44%	56%
	Shinyanga	50%	76%	36%	48%	39%	65%	52%	40%
	Simiyu	64%	69%	37%	38%	34%	58%	50%	35%
	Singida	50%	74%	48%	81%	34%	70%	60%	47%
	Tabora	39%	64%	39%	30%	44%	63%	46%	34%
	Tanga	50%	70%	52%	49%	34%	80%	56%	46%
**Uganda**	Central 1	64%	46%	44%	20%	27%	81%	47%	61%
	Central 2	69%	31%	54%	64%	23%	65%	51%	46%
	East central	53%	46%	56%	71%	27%	70%	54%	44%
	Eastern	50%	51%	56%	72%	37%	86%	59%	49%
	Kampala	47%	36%	60%	82%	23%	76%	54%	59%
	North	22%	21%	39%	36%	17%	61%	33%	44%
	Southwest	33%	51%	40%	39%	36%	72%	45%	39%
	West-nile	66%	49%	48%	65%	22%	64%	52%	44%
	Western	34%	42%	43%	39%	24%	73%	43%	49%

For peer review only - http://bmjopen.bmj.com/site/about/guidelines.xhtml

The RECORD statement – checklist of items, extended from the STROBE statement, that should be reported in observational studies using routinely collected health data.

	Item No.	STROBE items	Location in manuscript where items are reported	RECORD items	Location in manuscript where items are reported
**Title and abstract**					
	1	(a) Indicate the study's design with a commonly used term in the title or the abstract (b) Provide in the abstract an informative and balanced summary of what was done and what was found	(a) line 23, page 2<br><br>(b) lines 20-43, page 2	RECORD 1.1: The type of data used should be specified in the title or abstract. When possible, the name of the databases used should be included.<br><br>RECORD 1.2: If applicable, the geographic region and timeframe within which the study took place should be reported in the title or abstract.<br><br>RECORD 1.3: If linkage between databases was conducted for the study, this should be clearly stated in the title or abstract.	1.1: lines 23-24, page 2<br><br>1.2: lines 25-27, page 2<br><br>1.3: lines 23-24, page 2
**Introduction**					
Background rationale	2	Explain the scientific background and rationale for the investigation being reported	Lines 59-91, page 4		
Objectives	3	State specific objectives, including any prespecified hypotheses	Lines 92-95, page 4		
**Methods**					
Study Design	4	Present key elements of study design early in the paper	Lines 98-103, page 4		
Setting	5	Describe the setting, locations, and relevant dates, including periods of recruitment, exposure, follow-up, and data collection	Lines 100-102, page 4-5<br>Table S1- Supplementary file 1		

For peer review only

Participants	6	*(a) Cohort study* - Give the eligibility criteria, and the sources and methods of selection of participants. Describe methods of follow-up<br>*Case-control study* - Give the eligibility criteria, and the sources and methods of case ascertainment and control selection. Give the rationale for the choice of cases and controls<br>*Cross-sectional study* - Give the eligibility criteria, and the sources and methods of selection of participants<br><br>*(b) Cohort study* - For matched studies, give matching criteria and number of exposed and unexposed<br>*Case-control study* - For matched studies, give matching criteria and the number of controls per case	Lines 98-117, page 4-5<br>Table S1 , supplementary file 1	RECORD 6.1: The methods of study population selection (such as codes or algorithms used to identify subjects) should be listed in detail. If this is not possible, an explanation should be provided.<br><br>RECORD 6.2: Any validation studies of the codes or algorithms used to select the population should be referenced. If validation was conducted for this study and not published elsewhere, detailed methods and results should be provided.<br><br>RECORD 6.3: If the study involved linkage of databases, consider use of a flow diagram or other graphical display to demonstrate the data linkage process, including the number of individuals with linked data at each stage.	6.1: NA- secondary analysis of publicly available anonymized data was used<br>6.2: NA- secondary analysis of publicly available anonymized data was used<br><br>6.3: Figure S1 Supplementary file 1
Variables	7	Clearly define all outcomes, exposures, predictors, potential confounders, and effect modifiers. Give diagnostic criteria, if applicable.	Lines 118-141, Page 5<br><br>Table S2, supplementary file 1	RECORD 7.1: A complete list of codes and algorithms used to classify exposures, outcomes, confounders, and effect modifiers should be provided. If these cannot be reported, an explanation should be provided.	Lines 118-141, Page 5<br><br>Table S2, supplementary file 1
Data sources/ measurement	8	For each variable of interest, give sources of data and details of methods of assessment (measurement).<br>Describe comparability of assessment methods if there is more than one group	Lines 118-141, Page 5<br><br>Table S2, supplementary file 1		

For peer review only

Bias	9	Describe any efforts to address potential sources of bias	Lines 156-161, page 6		
Study size	10	Explain how the study size was arrived at	Secondary analysis of public data was conducted.<br>The selected sample is explained in lines 98-117, page 4-5<br>Table S1 , supplementary file 1		
Quantitative variables	11	Explain how quantitative variables were handled in the analyses. If applicable, describe which groupings were chosen, and why	Lines 143-161, page 6		
Statistical methods	12	(a) Describe all statistical methods, including those used to control for confounding<br>(b) Describe any methods used to examine subgroups and interactions<br>(c) Explain how missing data were addressed<br>(d) *Cohort study* - If applicable, explain how loss to follow-up was addressed<br>*Case-control study* - If applicable, explain how matching of cases and controls was addressed<br>*Cross-sectional study* - If applicable, describe analytical methods taking account of sampling strategy<br>(e) Describe any sensitivity analyses	a) lines 142-161, page 6<br><br>b) NA<br><br>c)lines 150, 151 - page 6<br><br>d) NA<br><br>e) NA		

For peer review only

Data access and cleaning methods		..		RECORD 12.1: Authors should describe the extent to which the investigators had access to the database population used to create the study population.<br><br>RECORD 12.2: Authors should provide information on the data cleaning methods used in the study.	12.1: Data is publicly available so section 12.1 does not apply<br><br>12.2: NA as access to already cleaned data was provided through DHS website
Linkage		..		RECORD 12.3: State whether the study included person-level, institutional-level, or other data linkage across two or more databases. The methods of linkage and methods of linkage quality evaluation should be provided.	12.3: lines 143-152, pages 6
**Results**					
Participants	13	(a) Report the numbers of individuals at each stage of the study (*e.g.*, numbers potentially eligible, examined for eligibility, confirmed eligible, included in the study, completing follow-up, and analysed)<br>(b) Give reasons for non-participation at each stage.<br>(c) Consider use of a flow diagram	Lines 168-169, page 6	RECORD 13.1: Describe in detail the selection of the persons included in the study (*i.e.,* study population selection) including filtering based on data quality, data availability and linkage. The selection of included persons can be described in the text and/or by means of the study flow diagram.	Supplementary Table 1 and Supplementary Figure 2 in Supplementary Methods
Descriptive data	14	(a) Give characteristics of study participants (*e.g.*, demographic, clinical, social) and information	Demographic of patients is not applicable; characteristics of		

For peer review only

		on exposures and potential confounders<br>(b) Indicate the number of participants with missing data for each variable of interest<br>(c) *Cohort study* - summarise follow-up time (*e.g.*, average and total amount)	included surveys are described in Supplementary Table 1 - supplementary methods		
Outcome data	15	*Cohort study* - Report numbers of outcome events or summary measures over time<br>*Case-control study* - Report numbers in each exposure category, or summary measures of exposure<br>*Cross-sectional study* - Report numbers of outcome events or summary measures	Table 1 – page 7<br>Table 2- page 8-9<br>Table 3- page 9-10		
Main results	16	(a) Give unadjusted estimates and, if applicable, confounder-adjusted estimates and their precision (e.g., 95% confidence interval). Make clear which confounders were adjusted for and why they were included<br>(b) Report category boundaries when continuous variables were categorized<br>(c) If relevant, consider translating estimates of relative risk into absolute risk for a meaningful time period	Table 2, page 8-9<br>&<br>Table 3, page 9-10<br>standard errors were included and confounders are added as footnotes<br><br>NA<br><br>NA		
Other analyses	17	Report other analyses done—e.g., analyses of subgroups and	NA		

For peer review only

		interactions, and sensitivity analyses			
**Discussion**					
Key results	18	Summarise key results with reference to study objectives	Lines 265-268, page 10		
Limitations	19	Discuss limitations of the study, taking into account sources of potential bias or imprecision. Discuss both direction and magnitude of any potential bias	Lines 316-330, page 11	RECORD 19.1: Discuss the implications of using data that were not created or collected to answer the specific research question(s). Include discussion of misclassification bias, unmeasured confounding, missing data, and changing eligibility over time, as they pertain to the study being reported.	Lines 316-330, page 11
Interpretation	20	Give a cautious overall interpretation of results considering objectives, limitations, multiplicity of analyses, results from similar studies, and other relevant evidence	Lines 268-315, page 10-11		
Generalisability	21	Discuss the generalisability (external validity) of the study results	NA – this is a multi-country study, all countries with available SPA survey were considered		
**Other Information**					
Funding	22	Give the source of funding and the role of the funders for the present study and, if applicable, for the original study on which the present article is based	Lines 349-351, page 12		
Accessibility of protocol, raw data, and programming code		..		RECORD 22.1: Authors should provide information on how to access any supplemental information such as the study protocol, raw data, or programming code.	Lines 343-345, page 12

For peer review only

*Reference: Benchimol EI, Smeeth L, Guttmann A, Harron K, Moher D, Petersen I, Sørensen HT, von Elm E, Langan SM, the RECORD Working Committee. The REporting of studies Conducted using Observational Routinely-collected health Data (RECORD) Statement. *PLoS Medicine* 2015; in press.

*Checklist is protected under Creative Commons Attribution (CC BY) license.

For peer review only

BMJ Open

Can One-Solution-Fit-All? A Subnational Observational Analysis of Primary Health Care Service Delivery in 12 Low- and Middle-Income Countries

Journal:	*BMJ Open*
Manuscript ID	bmjopen-2022-065223.R1
Article Type:	Original research
Date Submitted by the Author:	27-Sep-2022
Complete List of Authors:	Ramadan, Marwa; World Bank Group<br>Gutierrez, Jose Carlos; World Bank Group<br>Feil, Cameron; World Bank Group<br>Bolongaita, Sarah; World Bank Group<br>Bernal, Oscar; World Bank Group<br>Villar-Uribe , Manuela; World Bank Group
**Primary Subject Heading**:	Global health
Secondary Subject Heading:	Global health, Health policy, Public health
Keywords:	PRIMARY CARE, Quality in health care < HEALTH SERVICES ADMINISTRATION & MANAGEMENT, PUBLIC HEALTH, International health services < HEALTH SERVICES ADMINISTRATION & MANAGEMENT

SCHOLARONE™
Manuscripts

I, the Submitting Author has the right to grant and does grant on behalf of all authors of the Work (as defined in the below author licence), an exclusive licence and/or a non-exclusive licence for contributions from authors who are: i) UK Crown employees; ii) where BMJ has agreed a CC-BY licence shall apply, and/or iii) in accordance with the terms applicable for US Federal Government officers or employees acting as part of their official duties; on a worldwide, perpetual, irrevocable, royalty-free basis to BMJ Publishing Group Ltd ("BMJ") its licensees and where the relevant Journal is co-owned by BMJ to the co-owners of the Journal, to publish the Work in this journal and any other BMJ products and to exploit all rights, as set out in our licence.

The Submitting Author accepts and understands that any supply made under these terms is made by BMJ to the Submitting Author unless you are acting as an employee on behalf of your employer or a postgraduate student of an affiliated institution which is paying any applicable article publishing charge ("APC") for Open Access articles. Where the Submitting Author wishes to make the Work available on an Open Access basis (and intends to pay the relevant APC), the terms of reuse of such Open Access shall be governed by a Creative Commons licence – details of these licences and which Creative Commons licence will apply to this Work are set out in our licence referred to above.

Other than as permitted in any relevant BMJ Author's Self Archiving Policies, I confirm this Work has not been accepted for publication elsewhere, is not being considered for publication elsewhere and does not duplicate material already published. I confirm all authors consent to publication of this Work and authorise the granting of this licence.

For peer review only

Can One-Solution-Fit-All? A Subnational Observational Analysis of Primary Health Care Service Delivery in 12 Low-and Middle-Income Countries

Authors and affiliations

Marwa Ramadan[1]*, Jose Carlos Gutierrez[1], Cameron Feil[1], Sarah Bolongaita[1], Oscar Bernal[1], Manuela Villar Uribe[1]

[1]Health Nutrition and Population Global Practice, World Bank Group, Washington, DC, USA.

***Corresponding Author**

Marwa Ramadan

Health Nutrition and Population Global Practice, World Bank Group, Washington, DC, USA

mramadan@worldbank.org

Keywords

Primary healthcare; healthcare quality; measurement; performance assessment; subnational; Low- and Middle-income countries

For peer review only

For peer review only - http://bmjopen.bmj.com/site/about/guidelines.xhtml

Abstract

Objectives: To examine the capacity and quality of Primary Health Care (PHC) service delivery at the subnational level in 12 Low-and Middle-Income Countries (LMICs) and its association with intermediate health outputs such as coverage and access to care.

Design: Observational cross-sectional study using matched subnational data from service provision assessment surveys and demographic health surveys.

Settings: 12 LMICs (Afghanistan, Bangladesh, Democratic Republic of Congo, Haiti, Kenya, Malawi, Namibia, Nepal, Rwanda, Senegal, Tanzania, Uganda),

Participants: 138 sub-national areas with available survey data from 2007-2019.

Outcomes: Eight intermediate maternal and child health outcomes/outputs were explored: 1) met need for family planning by modern methods; 2) attendance of four or more antenatal care visits; 3) perceived financial barriers to care; 4) perceived geographic barriers to care; 5) Diphtheria-Pertussis-Tetanus (DPT) third dose coverage; 6) DPT dropout-rate; 7) care seeking for pneumonia; and 8) oral rehydration solutions coverage.

Results: Overall, moderate to poor PHC performance was observed across the 12 countries, with substantial heterogeneity between the different subnational areas in the same country as well as within the same subnational area across both capacity and quality subdomains. The analysis of the relationship between PHC service delivery and child health outcomes revealed that recent supervision [$b=0.34$, $p<0.01$] and supervisors' feedback [$b=0.28$, $p<0.05$] were each associated with increased care seeking for pneumonia. We also observed the associations of several measures of capacity and quality with DPT immunization. The analysis of maternal health outcomes yielded only a few statistically significant results at $p<0.05$ level, however, none remained significant after adjusting for other covariates.

Conclusion: The results of this observational analysis illustrate the heterogeneity in the capacity and quality of PHC service delivery within LMICs. Countries seeking to strengthen their PHC systems could improve PHC monitoring at the subnational level to better understand subnational bottlenecks in service delivery.

For peer review only - http://bmjopen.bmj.com/site/about/guidelines.xhtml

Strengths and limitations of this study:

- This study bridges a research gap by focusing on Primary Healthcare (PHC) service delivery and its association with improved health outputs at the subnational level in 12 low-and Middle-income countries.
- The methodology utilized in the present study demonstrates how publicly available survey data such as the Demographic Health Survey (DHS) and the Service Provision Assessment (SPA) survey can be valuable tools in highlighting bottlenecks and disparities in PHC service delivery at the subnational level.
- As the study is observational and cross-sectional, the association presented should not be interpreted as causal given the potential for an ecological bias.
- Although the sample was constructed such that the time between the SPA and DHS surveys in each country was less than 5 years, it is possible that changes in time-varying factors could have influenced the outcomes under study.
- There is a potential for omitted variable bias through other factors that may influence the capacity, quality, and performance variables under study but are unmeasured in the SPA and DHS surveys.

For peer review only

Introduction

Despite renewed global commitment to primary healthcare (PHC) in recent years, the measurement of PHC capacity and quality remains a challenge for policymakers seeking to improve the performance of health services. The transition from the Millennium Development Goals (MDGs) to Sustainable Development Goals (SDGs) in 2016 marked a significant shift in the global health agenda. SDG 3, "Achieving Universal Healthcare Coverage," was a call to action to strengthen health systems, shifting the global health agenda away from vertically planned health programs and initiatives. In 2018, forty years after the Alma-Alta Declaration which identified PHC as the pathway to achieving health for all, global actors, governments, and organizations re-affirmed a global commitment to PHC with the issuance of the Astana Declaration (1).

More significant efforts are required to improve primary health care performance in low-and-middle-income countries (2). Performance measurement has the potential to catalyze PHC improvement by identifying bottlenecks (3,4). The Primary Health Care Performance Initiative (PHCPI), a global collaborative dedicated to measuring and improving PHC systems in LMICs, developed a conceptual framework illustrating the relationships between financing, inputs, and core functions of the PHC system (2). In 2018, PHCPI operationalized the Conceptual Framework into the Vital Signs Profile, which uses qualitative and quantitative indicators to provide a comprehensive understanding of PHC performance in a county (3).

The relationship between capacity, quality and health system performance has long been studied. Donabedian captured the causal chain of inputs to outputs, commonly defined as the 'input-process-output-model', in 1966. More recent efforts by Randhawa (4) and Veillard et al.(5) have demonstrated the relationship between inputs and outcomes of PHC system. Previous studies also highlighted that PHC capacity, including the availability of providers, governance, and financing, has been found to impact PHC-related outcomes in LMICs at the national level (6–10). In addition, several studies have identified relationships between elements of high-quality PHC and performance at the national level (2,11–14). However, the majority of associations were explored at the national level and significant gaps still exist in understanding PHC service delivery at the sub-national level and its association with improved health outputs.

This paper aims to fill some of the current measurement gaps by exploring the relationship between PHC system capacity, quality, and performance at the sub-national level using quantitative indicators from the PHCPI Vital Signs Profile. Specifically, we assess PHC service delivery at both the national and subnational levels in 12 LMICs. In addition, we examine the association of PHC capacity and quality with intermediate health outputs such as coverage and access to services at the subnational level.

Methods

Data sources

In this cross-sectional analysis, Service Provision Assessment (SPA) surveys and Demographic Health Surveys (DHS) were the main sources of data on PHC service delivery and PHC intermediate population health outputs, respectively in 12 low-and Middle-income countries (Afghanistan, Bangladesh, DRC (democratic republic of Congo), Haiti, Kenya, Malawi, Namibia, Nepal, Rwanda, Senegal, Tanzania, Uganda). Specifically, we included all LMICS (as defined by the world bank income classification) where both a DHS and a SPA survey were conducted within a 5-year interval in the period between 2007 and 2019. SPA surveys are comprehensive facility assessments that aim to collect information on the

availability of services at the facility level, the readiness of facilities to provide these services, the compliance with acceptable standards of care, and whether clients and service providers are satisfied with the service delivery process (15). SPA surveys usually involve random selection of 400-700 facilities from a comprehensive sampling list of all public and private health facilities in a country or alternatively a complete census of facilities in the country. Facilities are usually categorized by facility type, managing authority (public and non-public), as well as subnational region; sampling and sample size ensure representativeness across each of these categories. Weights are applied during the analysis of these surveys to ensure that the contribution of selected facilities is proportional to their distribution in the country. On the other hand, DHS are nationally representative household surveys and a vital source of information on population, health, and nutrition indicators in more than 90 countries. The standard DHS survey is conducted every five years using a large sample size (5000-30,000 households per survey), and usually utilizes a two-stage sampling methodology based on the availability of census information (16). The DHS sampling and sample size ensure representativeness at the subnational region level. Table 1 provides additional information on the sampling structure and the timeframe for data collection in each of the studied countries. The SPA and DHS datasets utilized in this study are publicly available and can be downloaded from the DHS website (17).

Table 1: Characteristics of DHS and SPA surveys included in the analysis

Country	Sub-national areas	DHS Field Work	DHS sampling	DHS sample size (ever-married Women)	Year data collection -SPA	SPA sample Size (Facilities)
**Afghanistan**	34 provinces	June 2015 - February 2016	two-stage stratified sample	24,395	November 2018- January 2019	160 facilities**
**Bangladesh**	8 divisions	October 24, 2017- March 2018	two-stage stratified sample	20,100	July 2017-to October 2017	1,600
**DRC**	26 new provinces	August 2013 - February 2014	Three-stage stratified sample*	18,827	October 2017 - April 2018	1380
**Haiti**	10 departments	November 2016 - April 2017	two-stage stratified sample	14,371	December 2017 - May 2018	1007
**Kenya**	8 regions	May 2014 - October 2014	two-stage stratified sample	31,079	January 2010- May 2010	695
**Malawi**	3 regions	October 2015 - February 2016	two-stage stratified sample	24562	June 2013- February 2014	1060
**Namibia**	13 regions	May 2013 - September 2013	two-stage stratified sample	9176	July 2009- October 2009	446
**Nepal**	7 provinces	June 2016 - January 2017	Stratified sample (two stages in rural areas and three stages in urban areas)	12862	April 2015- November 2015	1,000

For peer review only - http://bmjopen.bmj.com/site/about/guidelines.xhtml

**Rwanda**	5 provinces	September 2010 - March 2011	two-stage stratified sample	13671	June 2007- August 2007	538
**Senegal**	14 regions	April 2017 - December 2017	two-stage stratified sample	16787	March 2017- December 2017	3764
**Tanzania**	25 regions	August 2015 - February 2016	two-stage stratified sample	13266	October 2014 – March 2015	1,200
**Uganda**	15 regions	June 2011 - December 2011	two-stage stratified sample	8674	July-October 2007	491

*Two-stage stratified sample in statutory towns and cities of established provinces, three-stage stratified sample in rest of established provinces and new provinces, **Sampling limited to urban areas of seven major provinces (Kabul, Nangarhar, Paktya, Kunduz, Balkh, Kandahar, and Herat)

Metrics

Guided by the PHCPI framework and survey data availability, the capacity of the PHC system was assessed using the following five indicators: 1) availability of basic facility Infrastructure; 2) the presence of recent supervision within the past 6 months; 3) the provision of feedback by supervisors; 4) the provision of outreach services; and 5) absence of PHC fees defined as free immunization, RMNCH, non-communicable diseases and HIV services. Similarly, whenever data was available, PHCPI's Vital Signs Profile (VSP) guided the assessment of PHC quality of care through the following six indicators: 1) waiting time of less than 60 minutes; 2) availability of providers defined as an average consultation time of more than 10 minutes; 3) competence of providers defined as the average of antenatal care, family planning and sick child quality scores based on WHO guidelines; 4) person-centeredness defined as the percent of clients who were told their diagnosis; 5) safety defined as the presence of adequate infection control practices and waste disposal at the facility; and 6) comprehensiveness of PHC services defined as the average availability of tracer items for RMNCH, infectious diseases and non-communicable diseases. Additional information on PHCPI's framework and methodology is provided in an earlier publication (5).

Additionally, eight intermediate maternal and child health outcomes/outputs were explored in accordance with the PHCPI vital signs profile and consistent reporting of DHS indicators in the studied countries. Specifically, we assessed the following maternal and child health outputs: 1) met need for family planning by modern methods; 2) attendance of four or more antenatal care visits; 3) perceived financial barriers to care; 4) perceived geographic barriers to care; 5) DPT3 coverage; 6) DPT dropout-rate; 7) care seeking for pneumonia; and 8) Oral Rehydration Solutions (ORS) coverage. Other PHC-related outputs (e.g., non-communicable diseases, tuberculosis and HIV) were not assessed due to the limited availability of sub-national data in the studied countries. Detailed definition of each of the capacity, quality and output indicators are provided in Supplementary Table 1 [See Supplementary Material]

For peer review only - http://bmjopen.bmj.com/site/about/guidelines.xhtml

Data analysis

Population and health facility data were linked at the first administrative level (e.g., region, province…etc.) whenever data was available and the gap between a population-based survey and a health facility survey was less than five years. In situations where substantial variation existed at the first administrative level between the two types of surveys, geographic coordinates of health facilities and DHS clusters were input into geographic information system (GIS) software (ArcGIS) for matching. Specifically, in the Democratic Republic of Congo, DHS data in 2013 were reported for 11 formerly defined provinces, while 2017 SPA data were reported for 26 newly defined provinces. ArcGIS was used to match subnational areas in both types of surveys. In addition, in Afghanistan, the SPA survey was only conducted in seven urban subnational areas, so the match process with DHS was limited to these areas [See Supplementary Figure 1 in Supplementary Material]

To compute subnational estimates, we applied the recommended SPA weights at the facility, provider, and visit levels in the 12 studied countries. We then computed national averages based on the assumption that all subnational areas were equally weighted in each of the studied countries. In addition, we examined the subnational association between the computed service delivery sub-domains and DHS intermediate maternal and child health outputs using bivariate and multivariable models with a significance level at $p<0.05$. Given the hierarchical nature of the examined population and health facility data, we utilized a two-level fixed-effects model to account for possible clustering of the observations at the country level. We also adjusted for the following subnational population and facility characteristics: proportion of private facilities, proportion of hospitals, proportion of urban households, proportion of population in the 5th economic quintile (highest), and proportion of women with secondary or more education. The statistical analyses were all performed in STATA (version 17.0), tables were created using Microsoft Excel.

Patient and public involvement

This study relied on secondary analysis of publicly available datasets. Therefore, patients and the public were not involved in the formulation of this research.

Results

I. PHC service delivery at the national and subnational levels

In the present study, the capacity and quality of PHC service delivery were analyzed in 138 subnational areas in 12 LMICs. Substantial heterogeneity was observed across countries in the capacity subdomain. For example, the average availability of facility infrastructure (basic amenities and infection prevention) was as high as 80% in Afghanistan and as low as 48 % in DRC. Most facilities in the analyzed subnational areas reported the presence of recent supervision, with an average ranging from 94% in Bangladesh to 70% in Tanzania, while supervisor's feedback was provided in a relatively fewer number of facilities, with the highest average reported in Bangladesh (91%) and the lowest in Afghanistan (57 %). Meanwhile, the provision of facility outreach services varied largely across the studied countries, with 84 % of facilities in Nepal providing outreach services versus only 14 % of facilities in the analyzed subnational areas in Afghanistan. Similarly, large variation in charging PHC fees was observed across the sample; 99% of facilities in the analyzed subnational areas of Uganda did not charge fees for PHC services versus only 32 % in Tanzania. Table 2 shows the average score of individual capacity sub-domains in the analyzed subnational areas across the 12 studied countries.

For peer review only - http://bmjopen.bmj.com/site/about/guidelines.xhtml

When the quality-of-service delivery was assessed, most countries had moderate to poor scores in all quality sub-domains except waiting time and comprehensiveness, where some countries performed well. As with capacity, cross-country variation in quality sub-domains was observed. For example, the average waiting time was less than 60 minutes in more than 90 % of the facilities in the analyzed subnational areas of Afghanistan and Nepal versus only 37 % of the analyzed facilities in Rwanda. However, the average consultation time was more than 10 minutes in only 38 % of the analyzed Afghan facilities. Competence of providers was one of the quality sub-domains with the lowest average scores across the studied countries ranging from 30 % on average, for the analyzed facilities in Afghanistan to 54 % in Namibia. Similarly, person centeredness scored poorly in most countries with an average score as low as 9 % for the analyzed facilities in Senegal versus 54 % in Uganda. Meanwhile, at least two thirds of the analyzed facilities offered comprehensive PHC services including RMNCH, non-communicable and infectious disease services in all the studied countries (Table 2).

Table 2: The capacity and quality of PHC service delivery in the studied countries (average scores of subnational areas)

Domain	Subdomain	Country											
		AF18*	BD17	DRC18	HT17	KE10	MW13	NM09	NP15	RW07	SN17	TZ15	UG07
Capacity	Facility Infrastructure	80%	51%	48%	62%	66%	67%	69%	53%	63%	62%	56%	55%
	Recent Supervision	77%	94%	77%	74%	74%	74%	72%	72%	82%	77%	70%	81%
	Supervisor Feedback	57%	91%	72%	64%	63%	65%	61%	64%	74%	76%	65%	62%
	Outreach Services	14%	18%	73%	39%	43%	70%	20%	84%	79%	48%	50%	79%
	Not charging PHC Fees	50%	67%	42%	73%	67%	74%	94%	92%	96%	65%	32%	99%
	**Capacity score**	55%	65%	61%	60%	61%	70%	63%	73%	79%	64%	53%	76%
Quality	Waiting Time < 60 minutes	92%	-	76%	58%	61%	48%	44%	94%	37%	77%	57%	49%
	Provider Availability	38%	-	78%	65%	63%	41%	63%	44%	39%	62%	70%	42%
	Provider Competence	30%	-	39%	40%	41%	37%	54%	31%	41%	40%	40%	49%
	Person Centeredness	45%	-	31%	25%	38%	45%	54%	33%	26%	9%	46%	54%
	Safety	31%	39%	32%	55%	65%	49%	73%	45%	26%	65%	41%	26%
	Comprehensiveness	68%	63%	74%	79%	81%	75%	85%	76%	75%	65%	71%	72%
	**Quality**	50%	51%	55%	56%	61%	49%	62%	54%	41%	53%	54%	47%

AF18: Afghanistan 2018, BD17: Bangladesh 2017, DRC18: democratic republic of Congo 2018, HT17: Haiti 2017, KE10: Kenya 2010, MW13: Malawi 2013, NM09: Namibia 2009, NP15: Nepal 2015, RW07: Rwanda 2007, SN17: Senegal 2017, TZ15: Tanzania 2015, UG07: Uganda 2007
* Estimates represent the average of urban subnational data for which SPA and DHS data were available

Subnational analysis of service delivery sub-domains revealed the presence of geographic disparities at the first administrative level in both PHC capacity and quality sub-domains. For example, large subnational variation was observed in the average availability of facility infrastructure in Senegal,

For peer review only - http://bmjopen.bmj.com/site/about/guidelines.xhtml

ranging from 83 % in Diourbel region to 45 % in Kedougou region. Similarly, considerable subnational variation was observed in the provision of outreach services in Haiti, ranging from 45 % in North-East to 5 % in North-West department. Charging of PHC fees in DRC was also another example of administrative variation in PHC capacity, where 81 % of facilities in Sankuru province provided free PHC services versus none of the facilities in North-Kivu or South-Kivu provinces. Similarly, subnational variation was observed in most of the examined quality sub-domains. For example, the range of variation across the different subnational areas in Tanzania was more than 60 % points for waiting time. In addition, the range of variation was more than 50 % for availability of providers across the different subnational areas in Senegal. Furthermore, person centered care ranged from 89 % in Kunduz province to only 1 % in Kandahar province in Afghanistan. Meanwhile, the lowest subnational variations were observed in provider competence sub-domain, where the majority of subnational areas scored below 50 % in the 12 studied countries.

Not only did we observe variations within the same country across different subnational areas, but also within the same subnational area across both quality and capacity sub-domains. For example, the range of variation across different capacity sub-domains was more than 50 % points in the majority of the analyzed sub-national areas in Afghanistan, Bangladesh and Namibia. Most of these variations were derived by the difference in the capacity of the facilities in providing outreach services and offering free PHC services. Similarly, several subnational areas in Afghanistan, DRC and Senegal showed substantial variation across the different quality sub-domains. For example, the range of variation across quality sub-domains in Kandahar, Afghanistan was 99 %, where all facilities had a waiting time of less than 60 minutes; however only 1 % provided person centered care. Supplementary Table 2 and Supplementary Table 3 provide detailed information on the capacity and quality scores in each of the analyzed 138 subnational areas [See Supplementary Material]

II. The association between PHC service delivery sub-domains and intermediate health outputs

Examining the relationship between subnational PHC capacity and maternal health outputs, no statistically significant association was found between the capacity of facilities and antenatal care coverage after adjustment for subnational characteristics. Meanwhile, a statistically significant association was observed between the general availability of basic facility infrastructure and perceived economic and geographic barriers to care. Specifically, a 10 % point increase in the average availability of basic infrastructure at the subnational level was associated with 3 % lower perceived financial barriers to care [$b = -0.35$, $p < 0.05$] and 4 % lower perceived geographic barriers to care [$b = -0.39$, $p < 0.05$]. However, the associations were not statistically significant after adjustment for subnational facility and population characteristics (Table 3). Coverage of family planning services was also negatively associated with the provision of outreach services. However, this paradoxical association was no longer significant in the multivariable specification. Meanwhile, no statistically significant association was found between any of the examined PHC quality sub-domains and maternal health outputs (Table 3).

Table 3: The association between PHC service delivery subdomains and maternal health outputs at the subnational level

Sub-domain			Antenatal		Family planning		Cost Barrier		Geographic Barrier	
			Bivariate	Multivariable	Bivariate	Multivariable	Bivariate	Multivariable	Bivariate	Multivariable
Capacity	Facility Infrastructure	β	0.23	0.11	0.29	0.16	-0.35*	-0.23	-0.39*	-0.16
		SE	0.13	0.13	0.16	0.19	0.15	0.14	0.15	0.16
	Supervision in the past	β	-0.09	0.02	-0.12	0.01	0.05	-0.02	0.00	-0.14

For peer review only - http://bmjopen.bmj.com/site/about/guidelines.xhtml

For peer review only

	6 months	SE	0.08	0.08	0.09	0.10	0.09	0.08	0.10	0.09
	Supervisor Feedback	β	-0.06	0.01	-0.06	0.03	-0.03	-0.09	-0.02	-0.11
		SE	0.08	0.07	0.09	0.09	0.09	0.07	0.09	0.08
	Outreach Services	β	-0.07	0.00	-0.13*	-0.09	0.04	-0.01	0.09	0.04
		SE	0.05	0.04	0.06	0.06	0.06	0.05	0.06	0.05
	Not charging PHC fees	β	0.01	0.01	-0.01	0.00	-0.02	0.00	0.02	0.06
		SE	0.05	0.04	0.06	0.06	0.06	0.05	0.06	0.05
**Quality**	Waiting time < 60 minutes	β	-0.01	0.01	-0.11	-0.10	0.09	0.05	0.11	0.07
		SE	0.06	0.05	0.08	0.07	0.07	0.06	0.08	0.06
	Provider Competence	β	0.07	0.00	0.04	0.01	-0.28	-0.10	-0.26	-0.10
		SE	0.15	0.13	0.19	0.18	0.19	0.14	0.19	0.15
	Provider Availability	β	-0.04	-0.08	-0.16	-0.15	-0.11	-0.13	0.02	0.03
		SE	0.09	0.07	0.11	0.10	0.10	0.08	0.11	0.08
	Person centeredness	β	-0.01	-0.04	0.01	-0.02	-0.06	-0.01	-0.02	0.02
		SE	0.05	0.04	0.06	0.06	0.06	0.05	0.07	0.05
	Safety	β	0.09	0.06	0.01	-0.03	0.20	0.13	0.11	0.10
		SE	0.08	0.07	0.11	0.11	0.11	0.08	0.11	0.09
	Comprehensiveness	β	0.09	0.09	-0.05	-0.02	-0.20	-0.09	-0.14	-0.05
		SE	0.10	0.08	0.11	0.11	0.11	0.08	0.12	0.09

* p<0.05

In multivariable models, results were adjusted for proportion of private facilities, hospitals, urban households, population in the 5th economic quintile and women with secondary education or more at the sub-national level

Afghanistan was excluded from the analysis for sub-national sample limitation

On the other hand, PHC service delivery sub-domains were more likely to be statistically significantly associated with child health outputs. For example, a 10 % point increase in the availability of basic facility infrastructure was associated with an increase of 5 % in DPT3 immunization coverage [b =0.45, p<0.05] and a decrease of 3% in DPT-dropout rates [b = - 0.30, p<0.05]. Additionally, recent facility supervision [b=0.34, p<0.01] and provision of feedback by supervisors [b=0.28, p<0.05] were each associated with increased care seeking for pneumonia after adjustment for facility and population characteristics. Surprisingly, significantly lower immunization coverage and higher dropout rates were observed in subnational areas where facilities did not charge PHC fees, even after adjustment for facility and population characteristics (Table 4). Meanwhile, of the examined quality sub-domains, only comprehensiveness showed a statistically significant association with subnational DPT3 immunization coverage and lower drop-out rates. Specifically, a 10 % point increase in the comprehensiveness of PHC services was associated with 2 % improvement in DPT3 coverage and 2 % lower DPT-dropout rates at the subnational level [b= 0.22, p<0.05 and b=-0.21, p<0.01 respectively] (Table 4).

<u>Table 4: The association between PHC service delivery subdomains and child health outputs at the subnational level</u>

**Sub-domain**			**DPT3**		**DPT_ dropout**		**Pneumonia care seeking**		**ORS coverage**	
			Bivariate	Multivariable	Bivariate	Multivariable	Bivariate	Multivariable	Bivariate	Multivariable
**Capa**	Facility Infrastructure	β	0.54**	0.45*	-0.37**	-0.30*	0.18	0.23	-0.02	0.01
		SE	0.15	0.18	0.12	0.14	0.18	0.22	0.12	0.15
	Supervision in the past 6 months	β	-0.04	0.08	0.03	-0.07	0.23*	0.34**	0.08	0.08
		SE	0.09	0.10	0.07	0.08	0.11	0.12	0.07	0.08
	Supervisor	β	-0.03	0.05	0.01	-0.05	0.22*	0.28**	0.10	0.09

For peer review only - http://bmjopen.bmj.com/site/about/guidelines.xhtml

	Feedback	SE	0.09	0.09	0.07	0.07	0.10	0.11	0.07	0.07
	Outreach Services	β	-0.07	-0.02	0.06	0.01	-0.04	-0.05	0.05	0.04
		SE	0.06	0.06	0.04	0.05	0.07	0.07	0.05	0.05
	Not charging PHC fees	β	-0.14*	-0.17**	0.07	0.09	0.09	0.09	0.08	0.07
		SE	0.06	0.06	0.05	0.05	0.07	0.07	0.05	0.05
**Quality**	Waiting time < 60 minutes	β	-0.12	-0.11	0.08	0.07	0.06	0.06	-0.03	-0.03
		SE	0.07	0.07	0.06	0.05	0.08	0.08	0.06	0.06
	Provider Competence	β	0.09	0.03	-0.06	-0.02	0.13	0.14	-0.22	-0.19
		SE	0.19	0.18	0.14	0.14	0.21	0.21	0.14	0.14
	Provider Availability	β	-0.18	-0.17	0.13	0.12	0.11	0.11	-0.02	-0.06
		SE	0.10	0.10	0.08	0.08	0.12	0.12	0.08	0.08
	Person centeredness	β	-0.08	-0.10	0.06	0.08	0.04	0.03	-0.01	-0.02
		SE	0.06	0.06	0.05	0.05	0.07	0.07	0.05	0.05
	Safety	β	0.01	0.03	-0.03	-0.04	0.07	0.07	-0.09	-0.07
		SE	0.11	0.11	0.08	0.08	0.12	0.13	0.09	0.09
	Comprehensiveness	β	0.23*	0.22*	-0.22**	-0.21**	-0.10	-0.11	0.03	0.03
		SE	0.11	0.10	0.08	0.08	0.13	0.13	0.09	0.08

Standard errors in parentheses
* p<0.05, ** p<0.01
In multivariable models, results were adjusted for proportion of private facilities, hospitals, urban households, population in the 5th economic quintile and women with secondary education or more at the subnational level
Afghanistan was excluded from the analysis for subnational sample limitation

Discussion

Overall, the results of the descriptive analysis illustrate the heterogeneity in the capacity and quality of PHC service delivery within LMICs. The two-level analysis of maternal health outcomes yielded only a few statistically significant results at the p<0.05 level, however, none remained significant after adjusting for other covariates. This suggests that factors outside health facilities' control may play a greater role in determining mothers' care seeking decisions for antenatal care and family planning, and in shaping perceptions of cost and geographic barriers to care. For example, individual- and household-level factors such as the mother's education and socioeconomic status are associated with the use of family planning and antenatal care services. Nonetheless, supply-side constraints at the health system level such as supply chain challenges may also contribute to stockouts of essential FP commodities (18). Analysis at levels more granular than the subnational area (the first administrative level) may yield further insight into the relationship of supply-side PHC characteristics and maternal health outcomes.

Meanwhile, the two-level analysis of child health outcomes revealed significant associations between supervision and care-seeking for pneumonia, as well as associations of several measures of capacity and quality with DPT3 immunization. Supervision in the last 6 months and supervisor feedback are considered measures of PHC capacity in the PHCPI framework, and it is hypothesized that measures of capacity operate through improved quality of health services. In the case of care-seeking for pneumonia, it is possible that facilities with greater capacity and implementation of supervision deliver care perceived to be of higher quality, thus increasing mothers' likelihood of seeking healthcare for their child at the facility. One review finds inconclusive but promising evidence that supportive supervision can improve quality of care, noting that supervision alone is insufficient; health workers require training and the availability of medicines, diagnostics and equipment to provide quality care (19). Patient perceptions of a facility's quality of care also influence utilization and care seeking (20–26). There is also ample literature demonstrating the influence of various household and individual-level factors such as

For peer review only

For peer review only - http://bmjopen.bmj.com/site/about/guidelines.xhtml

cultural beliefs, disease severity, geographic location, wealth, education, and socioeconomic status on care-seeking behavior (27,28)

Finally, our results found several associations with DPT3 immunization and dropout that generally align with existing literature (facility infrastructure and comprehensiveness) and in the case of user fees, counter to what might be expected. In line with our findings, prior research in Haiti found that both infrastructure quality and the quality-of-service delivery were associated increased utilization of primary care services, including antenatal care and vaccination (29). Similarly, a study in rural India found that children in districts where more villages had a healthcare subcenter were more likely to receive at least one dose of DPT3, though no association with DPT3 dropout rates was observed (30). The results above also suggest that comprehensiveness, a measure of PHC quality, is also associated with better immunization outcomes. This is in line with prior studies documenting that children of mothers who are counseled during vaccination sessions are more likely to complete all three DPT doses (25).

One possible explanation behind the counterintuitive negative association between the absence of user fees and DPT3 immunization is that the estimated association may be confounded by other factors determining both user fee exemptions and immunization coverage. For example, subnational areas that are generally poorer and have greater barriers to care may be more likely adopt policies exempting user fees, yet those characteristics may also result in lower immunization rates. A second explanation under the interpretation that the observed association is unconfounded may be related to potential unintended consequences of user fee removal. While the benefits of removing user fees are well documented in boosting health service utilization (31–33), some have also expressed concerns that the process of user fee removal must be designed carefully such that facilities are able to adapt. The elimination of fees and reduction of fees without a corresponding increase in resources from government sources could unintentionally lead to increased pressure on human resources and the availability of essential commodities, including vaccines. Further, increased demand for curative services without an increase in the health workforce could result in curative services "crowding out" preventative services such as vaccination. Ultimately, further research into any potentially paradoxical relationship between user fees and DPT3 vaccination.

The study should also be interpreted in light of its limitations. As the study is observational and cross-sectional, the association presented should not be interpreted as causal effects. Using data aggregated at the subnational level also entails the potential for an ecological fallacy and requires caution in interpretation. In addition, while the sample was constructed such that the time between the SPA and DHS surveys in each country was less than 5 years, it is possible that changes in time-varying factors or other events in that interlude could influence the outcomes under study. Afghanistan's more limited sample size restricted to predominantly urban areas presents an additional limitation, therefore it was excluded from regression analysis to mitigate any potential confounding. Finally, though we control for selected characteristics at the subnational and individual levels, there is a potential for omitted variable bias through other factors that may influence the capacity, quality, and performance variables under study but are unmeasured in the SPA and DHS surveys. Ultimately, further research into the relationships between PHC capacity, quality and service delivery outcomes could adopt other methodologies to establish potential causal links. Taking advantage of repeated survey waves may provide one such strategy to track how these indicators may have changed over time and to what extent improvements in capacity and quality lead to better results.

Our findings have important policy implications. The heterogenicity observed in PHC service delivery within and between subnational areas highlight that there is not one solution that fits all. Countries and stakeholders seeking to strengthen their PHC systems could improve PHC monitoring at the subnational

For peer review only - http://bmjopen.bmj.com/site/about/guidelines.xhtml

level. Disaggregation of national indicators by geographic regions and adoption of subnational scorecards can potentially unmask real bottlenecks that hinder the overall country progress. In addition, a participatory and mixed-methods approach to monitoring is important to better understand the diverse challenges across subnational areas and develop tailored approaches to resolve the problems identified.

Conclusion

Substantial heterogeneity and disparities were observed across the 12 studied LMICS, as well as between and within subnational areas in both the capacity and quality subdomains. Countries seeking to strengthen their PHC systems could improve PHC monitoring at the subnational level to better understand the diverse challenges across subnational areas and develop tailored approaches to resolve the problems identified. The associations observed between capacity, quality and performance also provide potential avenues for further research and contribute to the literature on the determinants of care-seeking behavior and immunization.

Ethics statements

This study was based on secondary analysis of anonymized publicly available datasets. No ethics approval, or patient consent was need for the present analysis.

Availability of data and material

The original datasets analyzed during the current study are available from the DHS program website, https://dhsprogram.com/data/available-datasets.cfm (17). The analytical dataset generated during the current study is available from the corresponding author on reasonable request

Authors Contributions: MR and MVU were responsible for the conception of this study and for methods development. MR analyzed data. MR, JCG, CF prepared the first draft. All authors (MR, JCG, CF, SB, OB, MVU) reviewed results or reviewed and contributed to the final manuscript.

Funding: This research was supported, in whole or in part, by the Bill and Melinda Gates Foundation (Grant number INV000932), and it had no role in study design, data collection, data analysis, data interpretation or writing of the manuscript.

Competing interests: None declared.

Reference

1. World Health Organization, Organisation for Economic Co-operation and Development, International Bank for Reconstruction and Development. Delivering quality health services: a global imperative for universal health coverage [Internet]. Geneva: World Health Organization; 2018. Available from: https://apps.who.int/iris/handle/10665/272465

2. Bitton A, Fifield J, Ratcliffe H, Karlage A, Wang H, Veillard JH, et al. Primary healthcare system performance in low-income and middle-income countries: a scoping review of the evidence from 2010 to 2017. BMJ Glob Health. 2019 Aug 16;4(Suppl 8):e001551.

For peer review only

3. Vital Signs Profiles | PHCPI [Internet]. [cited 2022 May 18]. Available from: https://improvingphc.org/vital-signs-profiles

4. Randhawa H. Report of the Expert Consultation on Primary Care Systems Profiles & Performance (PRIMASYS). :35.

5. Veillard J, Cowling K, Bitton A, Ratcliffe H, Kimball M, Barkley S, et al. Better Measurement for Performance Improvement in Low- and Middle-Income Countries: The Primary Health Care Performance Initiative (PHCPI) Experience of Conceptual Framework Development and Indicator Selection. Milbank Q. 2017 Dec;95(4):836–83.

6. Nikoloski Z, Albala S, Montero AM, Mossialos E. The impact of primary health care and specialist physician supply on amenable mortality in Mexico (2000-2015): Panel data analysis using system-Generalized Method of Moments. Soc Sci Med. 2021 Jun;278:113937.

7. Schneider MT, Chang AY, Crosby SW, Gloyd S, Harle AC, Lim S, et al. Trends and outcomes in primary health care expenditures in low-income and middle-income countries, 2000–2017. BMJ Glob Health. 2021 Aug;6(8):e005798.

8. Basu S, Berkowitz SA, Phillips RL, Bitton A, Landon BE, Phillips RS. Association of Primary Care Physician Supply With Population Mortality in the United States, 2005-2015. JAMA Intern Med. 2019 Apr 1;179(4):506–14.

9. Hone T, Rasella D, Barreto M, Atun R, Majeed A, Millett C. Large Reductions In Amenable Mortality Associated With Brazil's Primary Care Expansion And Strong Health Governance. Health Aff (Millwood). 2017 Jan 1;36(1):149–58.

10. Russo LX, Scott A, Sivey P, Dias J. Primary care physicians and infant mortality: Evidence from Brazil. PLOS ONE. 2019 May 31;14(5):e0217614.

11. Ranabhat CL, Jakovljevic M, Dhimal M, Kim CB. Structural Factors Responsible for Universal Health Coverage in Low- and Middle-Income Countries: Results From 118 Countries. Frontiers in Public Health [Internet]. 2020 [cited 2022 Mar 14];7. Available from: https://www.frontiersin.org/article/10.3389/fpubh.2019.00414

12. Lozano R, Fullman N, Mumford JE, Knight M, Barthelemy CM, Abbafati C, et al. Measuring universal health coverage based on an index of effective coverage of health services in 204 countries and territories, 1990–2019: a systematic analysis for the Global Burden of Disease Study 2019. The Lancet. 2020 Oct 17;396(10258):1250–84.

13. Leslie HH, Malata A, Ndiaye Y, Kruk ME. Effective coverage of primary care services in eight high-mortality countries. BMJ Glob Health. 2017 Sep 4;2(3):e000424.

14. Nguhiu PK, Barasa EW, Chuma J. Determining the effective coverage of maternal and child health services in Kenya, using demographic and health survey data sets: tracking progress towards universal health coverage. Trop Med Int Health. 2017 Apr;22(4):442–53.

15. USAID. The DHS Program - Service Provision Assessments (SPA) [Internet]. Demographic Health Surveys. 2019 [cited 2020 Nov 28]. p. 1–2. Available from: https://dhsprogram.com/What-We-Do/Survey-Types/SPA.cfm

For peer review only - http://bmjopen.bmj.com/site/about/guidelines.xhtml

16. Croft TN, Marshall AM, Allen CK, Arnold F, Assaf S, Balian S. Guide to DHS statistics. Rockville: ICF. 2018;645.

[Dataset]17. The DHS Program - Available Datasets [Internet]. [cited 2022 Sep 19]. Available from: https://dhsprogram.com/data/available-datasets.cfm

18. Wulifan JK, Brenner S, Jahn A, De Allegri M. A scoping review on determinants of unmet need for family planning among women of reproductive age in low and middle income countries. BMC Womens Health. 2016 Jan 15;16:2.

19. Bailey C, Blake C, Schriver M, Cubaka VK, Thomas T, Martin Hilber A. A systematic review of supportive supervision as a strategy to improve primary healthcare services in Sub-Saharan Africa. International Journal of Gynecology & Obstetrics. 2016 Jan 1;132(1):117–25.

20. Rao KD, Sheffel A. Quality of clinical care and bypassing of primary health centers in India. Soc Sci Med. 2018 Jun;207:80–8.

21. Gauthier B, Wane W. Bypassing health providers: The quest for better price and quality of health care in Chad. Social Science & Medicine. 2011 Aug 1;73(4):540–9.

22. Kruk ME, Hermosilla S, Larson E, Mbaruku GM. Bypassing primary care clinics for childbirth: a cross-sectional study in the Pwani region, United Republic of Tanzania. Bull World Health Organ. 2014 Apr 1;92(4):246–53.

23. Alqossayir FM, Alkhowailed MS, Alammar AY, Alsaeed AA, Alamri YY, Rasheed Z. Factors associated with patients bypassing primary healthcare centres in Qassim Region, KSA. J Taibah Univ Med Sci. 2021 Dec;16(6):900–5.

24. Audo MO, Ferguson A, Njoroge PK. Quality of health care and its effects in the utilisation of maternal and child health services in Kenya. East Afr Med J. 2005 Nov;82(11):547–53.

25. Colvin CJ, Smith HJ, Swartz A, Ahs JW, de Heer J, Opiyo N, et al. Understanding careseeking for child illness in sub-Saharan Africa: a systematic review and conceptual framework based on qualitative research of household recognition and response to child diarrhoea, pneumonia and malaria. Soc Sci Med. 2013 Jun;86:66–78.

26. Bell SK, Delbanco T, Elmore JG, Fitzgerald PS, Fossa A, Harcourt K, et al. Frequency and Types of Patient-Reported Errors in Electronic Health Record Ambulatory Care Notes. JAMA Netw Open. 2020 Jun 1;3(6):e205867.

27. Geldsetzer P, Williams TC, Kirolos A, Mitchell S, Ratcliffe LA, Kohli-Lynch MK, et al. The Recognition of and Care Seeking Behaviour for Childhood Illness in Developing Countries: A Systematic Review. PLOS ONE. 2014 Apr 9;9(4):e93427.

28. Bruce N, Pope D, Arana B, Shiels C, Romero C, Klein R, et al. Determinants of Care Seeking for Children With Pneumonia and Diarrhea in Guatemala: Implications for Intervention Strategies. Am J Public Health. 2014 Apr;104(4):647–57.

For peer review only - http://bmjopen.bmj.com/site/about/guidelines.xhtml

29. Gage AD, Leslie HH, Bitton A, Jerome JG, Joseph JP, Thermidor R, et al. Does quality influence utilization of primary health care? Evidence from Haiti. Globalization and Health. 2018 Jun 20;14(1):59.

30. Ghosh A, Laxminarayan R. Demand- and supply-side determinants of diphtheria-pertussis-tetanus nonvaccination and dropout in rural India. Vaccine. 2017 Feb 15;35(7):1087–93.

31. Watson SI, Wroe EB, Dunbar EL, Mukherjee J, Squire SB, Nazimera L, et al. The impact of user fees on health services utilization and infectious disease diagnoses in Neno District, Malawi: a longitudinal, quasi-experimental study. BMC Health Services Research. 2016 Oct 20;16(1):595.

32. Nguyen HT, Zombré D, Ridde V, De Allegri M. The impact of reducing and eliminating user fees on facility-based delivery: a controlled interrupted time series in Burkina Faso. Health Policy and Planning. 2018 Oct 1;33(8):948–56.

33. Lagarde M, Barroy H, Palmer N. Assessing the effects of removing user fees in Zambia and Niger. J Health Serv Res Policy. 2012 Jan;17(1):30–6.

For peer review only

Supplementary Methods

Supplementary Table 1: Definition of capacity, quality and output indicators utilized in the analysis

Domain	Subdomain	Indicator	Definition	Data Source
**Capacity**	Facility Infrastructure	SRI for basic amenities and infection prevention	Service readiness index for basic amenities and infection prevention	SPA
	Recent Supervision	Supervision within the past 6 months	Proportion of providers within a facility with supervision in past 6 months	SPA
	Supervisor Feedback	Supervisor provided verbal or written feedback	Proportion of providers within a facility provided verbal or written supervision feedback	SPA
	Outreach Services	Facility provides outreach services	Facility provides sick child, growth monitoring or immunization outreach service	SPA
	Not charging PHC Fees	Facility does not charge PHC fees	Facility does not charge fees for PHC services (fees for a specific PHC service or a fixed fee that covers all services that a client receives)	SPA
**Quality**	Waiting Time < 60 minutes	Wait time less than one hour	Proportion of observed visits with wait times less than one hour	SPA
	Provider Availability	Proportion of visits over 10 minutes	The proportion of ANC, FP, LD and sick child visits that last for more than 10 minutes.	SPA
	Provider Competence	Antenatal care quality score based on WHO guidelines	Average quality score for observed ANC visits based on WHO ANC guidelines	SPA
		Family planning quality score based on WHO guidelines	Average quality score for observed family visits based on WHO family planning guidelines	
		Sick child quality score based on IMCI guidelines	Average quality score for observed sick child visits based on WHO Integrated Management of Childhood Illness (IMCI) guidelines	
	Person Centeredness	Proportion of caregivers who were told the sick child diagnosis	Proportion of observed sick child visits where the health worker told the child's caretaker what illness(es) the child has	SPA
	Safety	Proportion of rooms with all infection control items	Proportion of rooms (FP, sick child, ANC and NCD) where all infection control tracer items are present	SPA
		Adequate waste disposal	Average score (out of 3) on adherence to standards for disposing of medical and hazardous waste, sharps, and having guidelines for waste disposal in place.	
	Comprehensiveness	Proportion of 3 NCD services offering diagnosis and treatment (diabetes, CRD, CVD)	Proportion of non-combinable disease services provided and for which guidelines are available (diabetes, chronic respiratory disease, and chronic cardiovascular disease) across all facilities	SPA
		Proportion of 3 primary ID services provided (STI, TB, HIV)	Proportion of infectious diseases services provided and for which guidelines are available for sexually transmitted infections, tuberculosis, and HIV across all facilities.	SPA
		Average availability of 5 tracer RMNCH services (SC, Vacc, FP, ANC, PMTCT)	Proportion of maternal and child health services provided and for which guidelines are available (sick child, vaccination, family planning, antenatal care, and prevention of mother-to-child transmission of HIV) across all facilities.	SPA
**Outputs**	Maternal	Antenatal care coverage - at least four visits (%)	Antenatal care coverage (4+) visits is the percent of women with a live birth who received antenatal care (ANC) 4 or more times.	DHS
		Demand for family planning satisfied with modern methods (%)	Proportion of married or in-union women of reproductive age (aged 15-49 years) who are married or in a union and have their need for family planning satisfied with modern methods.	DHS
		Perceived barriers due to treatment costs	Percentage of women who report specific problems in accessing health care when they are sick due to issues related to getting money for treatment	DHS
		Perceived barriers due to distance	Percentage of women who report specific problems in accessing health care when they are sick due to distance that they have to travel for treatment	DHS
	Child	Coverage of DTP3 immunization	Diphtheria-tetanus-pertussis (DTP) coverage measures the percent of one-year-olds who have received three doses of the combined diphtheria, tetanus toxoid and pertussis vaccine in a given year.	DHS
		DPT Dropout rate	Percent of children who do not receive three doses of DTP after receiving an initial dose. Calculated as (DPT1-DPT3/DPT1)	DHS
		Children aged < 5 years with pneumonia symptoms taken to a healthcare provider (%)	Percentage of children under 5 years of age with suspected pneumonia (cough and difficulty breathing NOT due to a problem in the chest and a blocked nose) in the two weeks preceding the survey taken to an appropriate health facility or provider.	DHS
		Children aged < 5 years with diarrhea receiving oral rehydration salts	The percent of children with diarrhea, a leading cause of death in children under five, who received appropriate treatment with oral rehydration solution.	DHS

For peer review only

Supplementary Figure 1: Linkage of SPA and DHS datasets

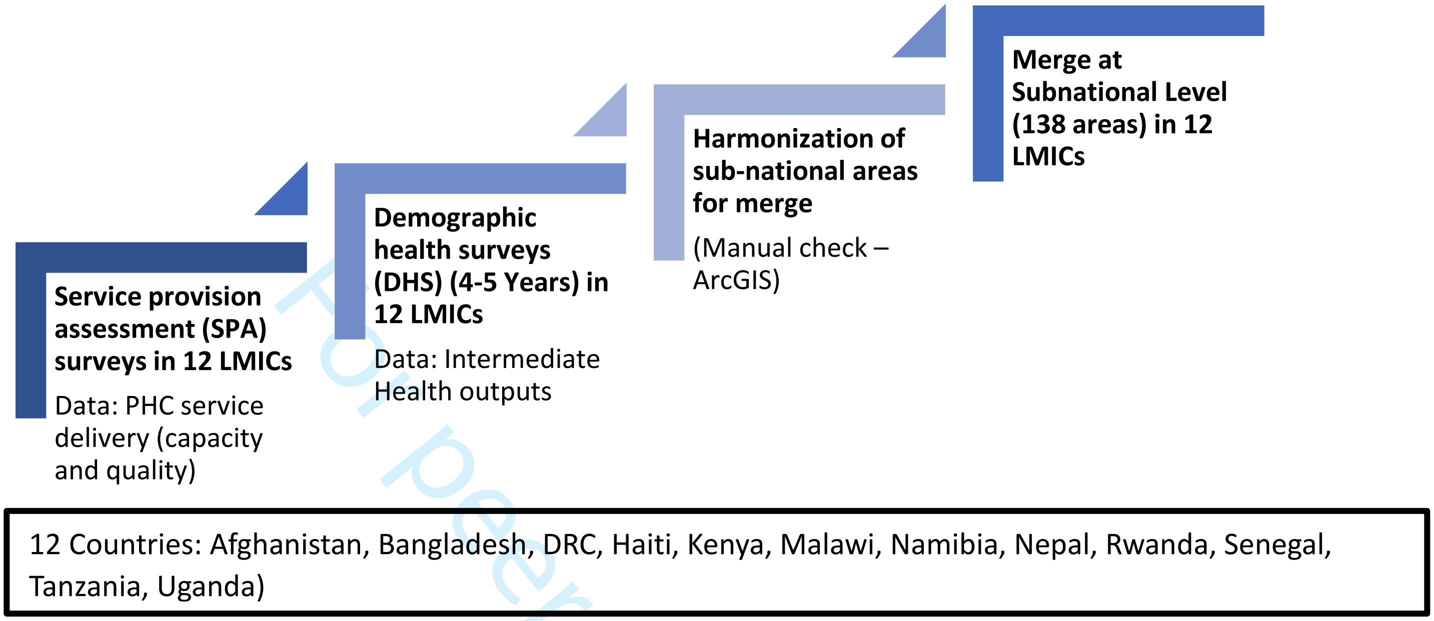

For peer review only

Supplementary Results

Supplementary Table 2: Capacity of PHC service delivery at the subnational level in the studied countries

Country	Division	Facility Infrastructure	Recent Supervision	Supervisor Feedback	Outreach Services	Not Charging PHC Fees	Average Capacity score	Range across capacity sub-domains*
Afghanistan	Balkh	83%	85%	63%	7%	56%	59%	78%
	Herat	85%	59%	29%	4%	71%	50%	81%
	Kabul	86%	63%	54%	31%	36%	54%	55%
	Kandahar	82%	99%	73%	29%	45%	66%	70%
	Kunduz	76%	91%	76%	0%	53%	59%	91%
	Nangarhar	75%	70%	63%	0%	45%	51%	75%
	Paktya	74%	70%	44%	25%	43%	51%	49%
Bangladesh	Barisal	49%	95%	93%	14%	67%	64%	81%
	Chittagong	51%	91%	85%	20%	80%	65%	71%
	Dhaka	52%	92%	89%	26%	54%	63%	66%
	Khulna	54%	97%	92%	21%	54%	64%	76%
	Mymensingh	46%	99%	96%	14%	45%	60%	85%
	Rajshahi	55%	96%	94%	19%	81%	69%	77%
	Rangpur	51%	97%	96%	14%	76%	67%	83%
	Sylhet	48%	89%	87%	18%	78%	64%	71%
DRC	Bas-uele	37%	68%	66%	91%	73%	67%	54%
	Equateur	42%	80%	71%	95%	24%	62%	71%
	Haut-katanga	45%	73%	63%	73%	46%	60%	28%
	Haut-lomami	53%	57%	49%	29%	56%	49%	28%
	Haut-uele	42%	75%	68%	81%	44%	62%	39%
	Ituri	51%	79%	74%	82%	30%	63%	52%
	Kasai	43%	72%	63%	25%	34%	47%	47%
	Kasai-central	49%	86%	86%	84%	40%	69%	46%
	Kasai-oriental	43%	83%	79%	74%	59%	67%	40%
	Kinshasa	60%	64%	56%	33%	66%	56%	33%

	Kongo central	55%	58%	54%	40%	58%	53%	18%
	Kwango	48%	91%	86%	95%	16%	67%	79%
	Kwilu	48%	71%	64%	90%	20%	58%	70%
	Lomami	47%	91%	91%	86%	37%	71%	54%
	Lualaba	54%	77%	76%	54%	65%	65%	23%
	Mai-ndombe	47%	85%	77%	92%	36%	67%	56%
	Maniema	57%	89%	85%	65%	65%	72%	32%
	Mongala	40%	87%	65%	78%	39%	62%	48%
	Nord-kivu	43%	91%	84%	94%	0%	63%	94%
	Nord-ubangi	55%	83%	82%	64%	34%	64%	49%
	Sankuru	36%	64%	56%	83%	81%	64%	47%
	Sud-kivu	49%	88%	85%	86%	0%	62%	88%
	Sud-ubangi	60%	75%	71%	69%	16%	58%	59%
	Tanganyka	47%	72%	71%	75%	64%	66%	28%
	Tshopo	41%	71%	67%	67%	52%	60%	30%
	Tshuapa	44%	84%	81%	94%	36%	68%	58%
**Haiti**	Artibonite	60%	78%	70%	67%	62%	67%	18%
	Centre	58%	90%	79%	70%	72%	74%	32%
	Grand'anse	66%	84%	76%	83%	78%	77%	18%
	Nippes	67%	64%	52%	29%	58%	54%	38%
	Nord	68%	73%	56%	42%	68%	61%	31%
	Nord-est	61%	66%	60%	45%	64%	59%	21%
	Nord-ouest	63%	58%	56%	4%	84%	53%	80%
	Ouest	59%	72%	63%	13%	76%	57%	63%
	Sud	56%	75%	49%	20%	98%	60%	78%
	Sud-est	63%	83%	75%	22%	68%	62%	61%
**Kenya**	Central	69%	76%	66%	64%	46%	64%	30%
	Coast	69%	71%	65%	53%	70%	66%	18%
	Eastern	71%	70%	60%	46%	64%	62%	25%
	Nairobi	61%	81%	72%	71%	72%	71%	20%
	North eastern	61%	83%	70%	42%	63%	64%	41%
	Nyanza	62%	69%	52%	31%	92%	61%	61%
	Rift valley	72%	67%	60%	14%	66%	56%	58%
	Western	61%	73%	62%	20%	63%	56%	53%

**Malawi**	Central region	68%	73%	64%	67%	67%	68%	9%
	Northern region	65%	75%	67%	78%	82%	73%	17%
	Southern region	69%	74%	65%	64%	72%	69%	10%
**Namibia**	Caprivi	58%	77%	49%	3%	97%	57%	94%
	Erongo	73%	63%	58%	18%	95%	61%	77%
	Hardap	78%	78%	69%	17%	83%	65%	66%
	Karas	72%	73%	68%	15%	92%	64%	77%
	Kavango	61%	71%	53%	21%	97%	60%	76%
	Khomas	74%	56%	50%	0%	75%	51%	75%
	Kunene	63%	69%	56%	14%	93%	59%	79%
	Ohangwena	66%	83%	68%	21%	100%	68%	79%
	Omaheke	75%	84%	82%	25%	100%	73%	75%
	Omusati	72%	79%	68%	42%	100%	72%	58%
	Oshana	68%	78%	66%	43%	100%	71%	57%
	Oshikoto	70%	70%	57%	18%	95%	62%	77%
	Otjozondjupa	74%	52%	47%	20%	97%	58%	77%
**Nepal**	Central	54%	71%	61%	73%	92%	70%	38%
	Eastern	54%	66%	60%	86%	93%	72%	39%
	Far-western	52%	76%	67%	88%	90%	75%	38%
	Mid-western	53%	71%	64%	88%	90%	73%	37%
	Western	54%	74%	69%	87%	94%	76%	40%
**Rowanda**	East	56%	91%	79%	81%	97%	81%	41%
	Kigali city	70%	56%	48%	69%	92%	67%	44%
	North	60%	84%	75%	80%	100%	80%	40%
	South	64%	91%	86%	87%	93%	84%	29%
	West	62%	90%	83%	77%	98%	82%	36%
**Senegal**	Dakar	79%	64%	62%	22%	67%	59%	57%
	Diourbel	83%	81%	80%	64%	60%	74%	23%
	Fatick	66%	91%	89%	37%	57%	68%	54%
	Kaffrine	47%	86%	86%	45%	72%	67%	41%
	Kaolack	51%	68%	67%	16%	66%	54%	52%
	Kedougou	45%	78%	78%	58%	66%	65%	33%
	Kolda	45%	86%	81%	61%	69%	69%	41%

	Louga	61%	58%	57%	53%	65%	59%	12%
	Matam	63%	73%	70%	58%	66%	66%	15%
	Saint-louis	62%	74%	74%	56%	60%	65%	18%
	Sedhiou	68%	100%	98%	51%	61%	75%	49%
	Tambacounda	61%	69%	66%	77%	66%	68%	16%
	This	61%	69%	68%	46%	67%	62%	23%
	Ziguinchor	70%	87%	86%	35%	64%	68%	52%
**Tanzania**	Arusha	62%	77%	68%	19%	14%	48%	63%
	Dar es salaam	49%	68%	67%	47%	11%	48%	57%
	Dodoma	73%	59%	55%	15%	52%	51%	58%
	Geita	54%	57%	54%	57%	17%	48%	40%
	Iringa	57%	58%	57%	58%	23%	51%	35%
	Kagera	55%	47%	41%	63%	15%	44%	48%
	Kaskazini pemba	59%	85%	78%	76%	29%	65%	56%
	Kaskazini unguja	62%	78%	74%	50%	86%	70%	36%
	Katavi	66%	83%	81%	40%	84%	71%	44%
	Kigoma	49%	80%	70%	82%	14%	59%	68%
	Kilimanjaro	52%	79%	66%	26%	25%	50%	54%
	Kusini pemba	61%	75%	66%	34%	41%	55%	41%
	Kusini unguja	61%	68%	64%	59%	86%	68%	27%
	Lindi	64%	78%	77%	15%	81%	63%	66%
	Manyara	52%	86%	77%	38%	26%	56%	60%
	Mara	53%	86%	78%	37%	35%	58%	51%
	Mbeya	54%	74%	70%	47%	22%	53%	52%
	Mjini magharibi	55%	59%	56%	59%	22%	50%	37%
	Morogoro	57%	65%	62%	56%	25%	53%	40%
	Mtwara	66%	45%	43%	16%	48%	44%	50%
	Mwanza	60%	48%	40%	73%	21%	49%	52%
	Njombe	51%	66%	56%	40%	13%	45%	53%
	Pwani	53%	54%	53%	51%	23%	47%	31%
	Rukwa	49%	44%	40%	54%	4%	38%	50%
	Ruvuma	51%	45%	36%	67%	28%	45%	39%
	Shinyanga	49%	95%	92%	70%	31%	67%	64%

	Simiyu	53%	79%	72%	64%	12%	56%	67%
	Singida	54%	90%	89%	75%	32%	68%	58%
	Tabora	51%	75%	74%	66%	15%	56%	60%
	Tanga	50%	90%	90%	38%	18%	57%	72%
**Uganda**	Central 1	52%	91%	78%	83%	100%	81%	48%
	Central 2	44%	91%	70%	77%	97%	76%	53%
	East central	55%	82%	69%	85%	100%	78%	45%
	Eastern	70%	60%	49%	57%	93%	66%	44%
	Kampala	54%	85%	68%	85%	100%	78%	46%
	North	45%	79%	34%	84%	100%	68%	66%
	Southwest	55%	82%	59%	82%	100%	76%	45%
	West-nile	55%	69%	57%	67%	100%	70%	45%
	Western	64%	93%	72%	94%	100%	85%	36%

For peer review only

Supplementary Table 3: Quality of PHC service delivery at the subnational level in the studied countries

Country	Division	Waiting Time	Provider Availability	Provider Competence	Person Centeredness	Safety	Comprehensive-ness	Average Quality score	Range across quality Subdomains *
Afghanistan	Balkh	89%	35%	28%	75%	28%	65%	53%	61%
	Herat	84%	34%	34%	50%	33%	52%	48%	51%
	Kabul	91%	20%	24%	34%	52%	67%	48%	71%
	Kandahar	100%	34%	19%	1%	55%	75%	47%	99%
	Kunduz	87%	42%	38%	89%	6%	69%	55%	83%
	Nangarhar	97%	48%	34%	45%	17%	78%	53%	80%
	Paktya	94%	54%	30%	22%	23%	73%	49%	72%
Bangladesh	Barisal	-	-	-	-	39%	67%	53%	28%
	Chittagong	-	-	-	-	39%	64%	52%	25%
	Dhaka	-	-	-	-	40%	61%	50%	21%
	Khulna	-	-	-	-	39%	64%	52%	25%
	Mymensingh	-	-	-	-	31%	65%	48%	34%
	Rajshahi	-	-	-	-	44%	68%	56%	24%
	Rangpur	-	-	-	-	45%	50%	47%	5%
	Sylhet	-	-	-	-	33%	68%	51%	35%
DRC	Bas-uele	74%	68%	39%	45%	35%	70%	55%	39%
	Equateur	90%	86%	36%	46%	28%	74%	60%	62%
	Haut-katanga	82%	89%	45%	40%	22%	86%	61%	67%
	Haut-lomami	78%	65%	43%	22%	45%	87%	57%	65%
	Haut-uele	63%	64%	34%	20%	26%	70%	46%	50%
	Ituri	89%	75%	39%	24%	39%	56%	54%	65%
	Kasai	95%	82%	30%	8%	19%	53%	48%	87%
	Kasai-central	73%	78%	39%	45%	30%	68%	56%	48%

	Kasai-oriental	69%	72%	38%	39%	28%	79%	54%	51%
	Kinshasa	44%	69%	48%	22%	29%	79%	48%	57%
	Kongo central	83%	88%	44%	33%	34%	88%	62%	55%
	Kwango	87%	87%	40%	11%	28%	71%	54%	76%
	Kwilu	65%	67%	37%	12%	37%	87%	51%	75%
	Lomami	70%	66%	37%	28%	36%	91%	55%	63%
	Lualaba	65%	63%	47%	40%	34%	91%	56%	57%
	Mai-ndombe	75%	86%	38%	23%	27%	78%	55%	63%
	Maniema	78%	71%	37%	46%	42%	94%	61%	57%
	Mongala	85%	89%	36%	50%	21%	71%	59%	68%
	Nord-kivu	65%	78%	42%	47%	30%	58%	54%	48%
	Nord-ubangi	77%	76%	48%	9%	49%	79%	56%	70%
	Sankuru	88%	87%	35%	30%	27%	49%	53%	61%
	Sud-kivu	75%	65%	40%	33%	36%	81%	55%	48%
	Sud-ubangi	71%	82%	44%	49%	43%	68%	60%	39%
	Tanganyka	80%	87%	33%	31%	42%	52%	54%	56%
	Tshopo	81%	88%	37%	20%	27%	71%	54%	68%
	Tshuapa	82%	88%	38%	41%	30%	69%	58%	58%
**Haiti**	Artibonite	50%	62%	36%	15%	51%	79%	49%	64%
	Centre	66%	70%	33%	7%	56%	88%	53%	81%
	Grand'anse	48%	71%	37%	8%	61%	85%	51%	77%
	Nippes	42%	59%	34%	18%	45%	75%	45%	57%
	Nord	52%	67%	36%	13%	55%	83%	51%	70%
	Nord-est	46%	68%	39%	10%	53%	77%	49%	67%
	Nord-ouest	64%	67%	49%	34%	76%	70%	60%	42%
	Ouest	70%	57%	44%	48%	51%	78%	58%	34%
	Sud	93%	54%	37%	44%	45%	64%	56%	56%
	Sud-est	53%	72%	58%	58%	60%	89%	65%	36%

Kenya	Central	30%	60%	36%	9%	55%	81%	45%	72%
	Coast	49%	63%	35%	14%	70%	90%	54%	76%
	Eastern	54%	63%	35%	15%	58%	83%	51%	68%
	Nairobi	53%	72%	32%	20%	56%	83%	52%	63%
	North eastern	73%	59%	44%	39%	66%	89%	62%	50%
	Nyanza	84%	34%	46%	71%	78%	72%	64%	50%
	Rift valley	65%	77%	50%	70%	71%	71%	67%	27%
	Western	75%	75%	51%	68%	67%	76%	69%	25%
Malawi	Central region	49%	43%	38%	51%	50%	75%	51%	37%
	Northern region	46%	42%	37%	41%	46%	77%	48%	40%
	Southern region	49%	39%	37%	43%	51%	72%	48%	35%
Namibia	Caprivi	30%	70%	52%	29%	81%	93%	59%	64%
	Erongo	59%	49%	52%	76%	65%	77%	63%	28%
	Hardap	49%	69%	51%	45%	86%	84%	64%	41%
	Karas	65%	59%	48%	45%	78%	87%	64%	42%
	Kavango	34%	48%	49%	30%	75%	94%	55%	64%
	Khomas	49%	69%	51%	84%	86%	55%	66%	37%
	Kunene	73%	76%	59%	79%	78%	86%	75%	27%
	Ohangwena	8%	55%	59%	63%	70%	93%	58%	85%
	Omaheke	58%	79%	67%	79%	91%	90%	77%	33%
	Omusati	39%	59%	49%	35%	69%	92%	57%	57%
	Oshana	25%	62%	60%	65%	59%	84%	59%	59%
	Oshikoto	34%	74%	64%	53%	54%	95%	62%	61%
	Otjozondjupa	46%	48%	47%	26%	61%	79%	51%	53%
Nepal	Central	96%	31%	26%	27%	42%	76%	50%	70%
	Eastern	90%	47%	31%	40%	44%	76%	55%	59%
	Far-western	94%	58%	34%	37%	42%	79%	57%	60%

	Mid-western	91%	45%	32%	27%	49%	74%	53%	64%
	Western	96%	41%	33%	37%	49%	75%	55%	63%
**Rowanda**	East	31%	41%	40%	13%	21%	79%	37%	66%
	Kigali city	52%	51%	45%	51%	31%	57%	48%	26%
	North	17%	34%	41%	27%	24%	76%	37%	59%
	South	43%	33%	35%	19%	32%	83%	41%	64%
	West	42%	37%	44%	18%	24%	78%	40%	60%
**Senegal**	Dakar	71%	63%	42%	24%	67%	70%	56%	47%
	Diourbel	99%	71%	42%	10%	81%	77%	63%	89%
	Fatick	94%	70%	42%	10%	72%	58%	58%	84%
	Kaffrine	71%	61%	47%	4%	53%	52%	48%	67%
	Kaolack	78%	69%	48%	29%	58%	53%	56%	49%
	Kedougou	86%	77%	49%	8%	45%	56%	53%	78%
	Kolda	76%	71%	44%	6%	49%	51%	50%	70%
	Louga	63%	49%	34%	1%	59%	74%	47%	73%
	Matam	72%	24%	28%	1%	75%	74%	46%	74%
	Saint-louis	43%	49%	33%	2%	68%	73%	45%	71%
	Sedhiou	91%	72%	38%	1%	79%	64%	58%	90%
	Tambacounda	62%	59%	32%	0%	69%	81%	50%	81%
	This	74%	60%	43%	20%	61%	64%	54%	54%
	Ziguinchor	97%	74%	37%	12%	74%	66%	60%	85%
**Tanzania**	Arusha	74%	76%	45%	44%	63%	59%	60%	32%
	Dar es salaam	59%	82%	42%	68%	35%	72%	60%	47%
	Dodoma	76%	76%	40%	44%	52%	56%	57%	36%
	Geita	71%	87%	35%	13%	26%	71%	51%	74%
	Iringa	42%	60%	44%	78%	30%	75%	54%	48%
	Kagera	76%	70%	45%	52%	37%	74%	59%	39%

	Kaskazini pemba	28%	72%	46%	77%	35%	76%	56%	49%
	Kaskazini unguja	71%	74%	39%	19%	27%	76%	51%	57%
	Katavi	22%	81%	47%	65%	66%	87%	61%	65%
	Kigoma	46%	74%	35%	67%	31%	83%	56%	52%
	Kilimanjaro	28%	56%	40%	41%	24%	83%	45%	59%
	Kusini pemba	84%	82%	35%	33%	54%	68%	59%	51%
	Kusini unguja	67%	82%	38%	23%	25%	74%	51%	59%
	Lindi	58%	77%	44%	67%	58%	89%	65%	45%
	Manyara	66%	61%	34%	57%	42%	63%	54%	32%
	Mara	54%	79%	48%	27%	47%	57%	52%	52%
	Mbeya	69%	74%	33%	24%	36%	61%	49%	50%
	Mjini magharibi	68%	59%	35%	46%	50%	78%	56%	43%
	Morogoro	61%	50%	36%	16%	61%	70%	49%	54%
	Mtwara	72%	57%	33%	50%	41%	66%	53%	39%
	Mwanza	41%	68%	38%	54%	44%	71%	53%	33%
	Njombe	55%	65%	43%	78%	36%	68%	58%	42%
	Pwani	41%	71%	47%	44%	36%	70%	52%	35%
	Rukwa	81%	76%	45%	27%	40%	60%	55%	54%
	Ruvuma	62%	43%	29%	19%	36%	75%	44%	56%
	Shinyanga	50%	76%	36%	48%	39%	65%	52%	40%
	Simiyu	64%	69%	37%	38%	34%	58%	50%	35%
	Singida	50%	74%	48%	81%	34%	70%	60%	47%
	Tabora	39%	64%	39%	30%	44%	63%	46%	34%
	Tanga	50%	70%	52%	49%	34%	80%	56%	46%
**Uganda**	Central 1	64%	46%	44%	20%	27%	81%	47%	61%
	Central 2	69%	31%	54%	64%	23%	65%	51%	46%

	East central	53%	46%	56%	71%	27%	70%	54%	44%
	Eastern	50%	51%	56%	72%	37%	86%	59%	49%
	Kampala	47%	36%	60%	82%	23%	76%	54%	59%
	North	22%	21%	39%	36%	17%	61%	33%	44%
	Southwest	33%	51%	40%	39%	36%	72%	45%	39%
	West-nile	66%	49%	48%	65%	22%	64%	52%	44%
	Western	34%	42%	43%	39%	24%	73%	43%	49%

For peer review only

For peer review only

For peer review only - http://bmjopen.bmj.com/site/about/guidelines.xhtml

The RECORD statement – checklist of items, extended from the STROBE statement, that should be reported in observational studies using routinely collected health data.

	Item No.	STROBE items	Location in manuscript where items are reported	RECORD items	Location in manuscript where items are reported
**Title and abstract**					
	1	(a) Indicate the study's design with a commonly used term in the title or the abstract (b) Provide in the abstract an informative and balanced summary of what was done and what was found	(a) line 24, page 2<br><br>(b) lines 21-45, page 2	RECORD 1.1: The type of data used should be specified in the title or abstract. When possible, the name of the databases used should be included.<br><br>RECORD 1.2: If applicable, the geographic region and timeframe within which the study took place should be reported in the title or abstract.<br><br>RECORD 1.3: If linkage between databases was conducted for the study, this should be clearly stated in the title or abstract.	1.1: lines 24-26, page 2<br><br>1.2: lines 26-28, page 2<br><br>1.3: lines 24-25, page 2
**Introduction**					
Background rationale	2	Explain the scientific background and rationale for the investigation being reported	Lines 63-89, page 4		
Objectives	3	State specific objectives, including any prespecified hypotheses	Lines 90-94, page 4		
**Methods**					
Study Design	4	Present key elements of study design early in the paper	Lines 97-119, page 4		
Setting	5	Describe the setting, locations, and relevant dates, including periods of recruitment, exposure, follow-up, and data collection	Lines 99-119, page 4-5<br>Table 1-page 5		

For peer review only

Participants	6	*(a) Cohort study* - Give the eligibility criteria, and the sources and methods of selection of participants. Describe methods of follow-up<br>*Case-control study* - Give the eligibility criteria, and the sources and methods of case ascertainment and control selection. Give the rationale for the choice of cases and controls<br>*Cross-sectional study* - Give the eligibility criteria, and the sources and methods of selection of participants<br><br>*(b) Cohort study* - For matched studies, give matching criteria and number of exposed and unexposed<br>*Case-control study* - For matched studies, give matching criteria and the number of controls per case	Lines 99-119, page 4-5<br>Table 1, page 5	RECORD 6.1: The methods of study population selection (such as codes or algorithms used to identify subjects) should be listed in detail. If this is not possible, an explanation should be provided.<br><br>RECORD 6.2: Any validation studies of the codes or algorithms used to select the population should be referenced. If validation was conducted for this study and not published elsewhere, detailed methods and results should be provided.<br><br>RECORD 6.3: If the study involved linkage of databases, consider use of a flow diagram or other graphical display to demonstrate the data linkage process, including the number of individuals with linked data at each stage.	6.1: NA- secondary analysis of publicly available anonymized data was used<br>6.2: NA- secondary analysis of publicly available anonymized data was used<br><br>6.3: Figure S1 Supplementary methods
Variables	7	Clearly define all outcomes, exposures, predictors, potential confounders, and effect modifiers. Give diagnostic criteria, if applicable.	Lines 125-147, Page 6<br><br>Table S1, supplementary methods	RECORD 7.1: A complete list of codes and algorithms used to classify exposures, outcomes, confounders, and effect modifiers should be provided. If these cannot be reported, an explanation should be provided.	Lines 125-145, Page 5<br><br>Table S1, supplementary methods
Data sources/ measurement	8	For each variable of interest, give sources of data and details of methods of assessment (measurement).<br>Describe comparability of assessment methods if there is more than one group	Lines 118-141, Page 5<br><br>Table S2, supplementary file 1		

For peer review only - http://bmjopen.bmj.com/site/about/guidelines.xhtml

Bias	9	Describe any efforts to address potential sources of bias	Lines 159-170, page 7		
Study size	10	Explain how the study size was arrived at	Secondary analysis of public data was conducted. The selected sample was explained<br>Lines 99-103, page 4-5<br>Table 1, page 5		
Quantitative variables	11	Explain how quantitative variables were handled in the analyses. If applicable, describe which groupings were chosen, and why	Lines 125-147, page 6		
Statistical methods	12	(a) Describe all statistical methods, including those used to control for confounding<br>(b) Describe any methods used to examine subgroups and interactions<br>(c) Explain how missing data were addressed<br>(d) *Cohort study* - If applicable, explain how loss to follow-up was addressed<br>*Case-control study* - If applicable, explain how matching of cases and controls was addressed<br>*Cross-sectional study* - If applicable, describe analytical methods taking account of sampling strategy<br>(e) Describe any sensitivity analyses	a) lines 149-170, page 7<br><br>b) NA<br><br>c)lines 153, 158 - page 7<br><br>d) NA<br><br>e) NA		

For peer review only

Data access and cleaning methods		..		RECORD 12.1: Authors should describe the extent to which the investigators had access to the database population used to create the study population.<br><br>RECORD 12.2: Authors should provide information on the data cleaning methods used in the study.	12.1: Data is publicly available so section 12.1 does not apply<br><br>12.2: NA as access to already cleaned data was provided through DHS website
Linkage		..		RECORD 12.3: State whether the study included person-level, institutional-level, or other data linkage across two or more databases. The methods of linkage and methods of linkage quality evaluation should be provided.	12.3: lines 149-158, pages 7
**Results**					
Participants	13	(a) Report the numbers of individuals at each stage of the study (*e.g.*, numbers potentially eligible, examined for eligibility, confirmed eligible, included in the study, completing follow-up, and analysed)<br>(b) Give reasons for non-participation at each stage.<br>(c) Consider use of a flow diagram	Lines 168-169, page 6	RECORD 13.1: Describe in detail the selection of the persons included in the study (*i.e.,* study population selection) including filtering based on data quality, data availability and linkage. The selection of included persons can be described in the text and/or by means of the study flow diagram.	Table 1, page 4-6 and Supplementary Figure 21 in Supplementary Methods
Descriptive data	14	(a) Give characteristics of study participants (*e.g.*, demographic, clinical, social) and information	Demographic of patients is not applicable; characteristics of		

For peer review only - http://bmjopen.bmj.com/site/about/guidelines.xhtml

		on exposures and potential confounders<br>(b) Indicate the number of participants with missing data for each variable of interest<br>(c) *Cohort study* - summarise follow-up time (*e.g.*, average and total amount)	included surveys are described in Table 1 -page 5		
Outcome data	15	*Cohort study* - Report numbers of outcome events or summary measures over time<br>*Case-control study* - Report numbers in each exposure category, or summary measures of exposure<br>*Cross-sectional study* - Report numbers of outcome events or summary measures	Table 2 – page 8<br>Table 3- page 9-10<br>Table 4- page 10-11		
Main results	16	(a) Give unadjusted estimates and, if applicable, confounder-adjusted estimates and their precision (e.g., 95% confidence interval). Make clear which confounders were adjusted for and why they were included<br>(b) Report category boundaries when continuous variables were categorized<br>(c) If relevant, consider translating estimates of relative risk into absolute risk for a meaningful time period	Table 3, page 9-10<br>&<br>Table 4, page 10-11 standard errors were included and confounders are added as footnotes<br>NA<br>NA		
Other analyses	17	Report other analyses done—e.g., analyses of subgroups and	NA		

For peer review only

		interactions, and sensitivity analyses			
**Discussion**					
Key results	18	Summarise key results with reference to study objectives	Lines 273-307, page 11-12		
Limitations	19	Discuss limitations of the study, taking into account sources of potential bias or imprecision. Discuss both direction and magnitude of any potential bias	Lines 323-337, page 12	RECORD 19.1: Discuss the implications of using data that were not created or collected to answer the specific research question(s). Include discussion of misclassification bias, unmeasured confounding, missing data, and changing eligibility over time, as they pertain to the study being reported.	Lines 323-337, page 12
Interpretation	20	Give a cautious overall interpretation of results considering objectives, limitations, multiplicity of analyses, results from similar studies, and other relevant evidence	Lines 273-322, page 11-12		
Generalisability	21	Discuss the generalisability (external validity) of the study results	NA – this is a multi-country study, all countries with available SPA survey were considered		
**Other Information**					
Funding	22	Give the source of funding and the role of the funders for the present study and, if applicable, for the original study on which the present article is based	Lines 364-366, page 13		
Accessibility of protocol, raw data, and programming code		..		RECORD 22.1: Authors should provide information on how to access any supplemental information such as the study protocol, raw data, or programming code.	Lines 358-360, page 13

For peer review only

*Reference: Benchimol EI, Smeeth L, Guttmann A, Harron K, Moher D, Petersen I, Sørensen HT, von Elm E, Langan SM, the RECORD Working Committee. The REporting of studies Conducted using Observational Routinely-collected health Data (RECORD) Statement. *PLoS Medicine* 2015; in press.

*Checklist is protected under Creative Commons Attribution (CC BY) license.

For peer review only

BMJ Open

Capacity and quality of maternal and child health services delivery at the subnational primary health care level in relation to intermediate health outputs: a cross-sectional study of 12 low-income and middle-income countries

Journal:	*BMJ Open*
Manuscript ID	bmjopen-2022-065223.R2
Article Type:	Original research
Date Submitted by the Author:	30-Nov-2022

Complete List of Authors:	Ramadan, Marwa; World Bank Group<br>Gutierrez, Jose Carlos; World Bank Group<br>Feil, Cameron; World Bank Group<br>Bolongaita, Sarah; World Bank Group<br>Bernal, Oscar; World Bank Group<br>Villar-Uribe , Manuela; World Bank Group

**Primary Subject Heading**:	Global health
Secondary Subject Heading:	Global health, Health policy, Public health
Keywords:	PRIMARY CARE, Quality in health care < HEALTH SERVICES ADMINISTRATION & MANAGEMENT, PUBLIC HEALTH, International health services < HEALTH SERVICES ADMINISTRATION & MANAGEMENT

SCHOLARONE™
Manuscripts

I, the Submitting Author has the right to grant and does grant on behalf of all authors of the Work (as defined in the below author licence), an exclusive licence and/or a non-exclusive licence for contributions from authors who are: i) UK Crown employees; ii) where BMJ has agreed a CC-BY licence shall apply, and/or iii) in accordance with the terms applicable for US Federal Government officers or employees acting as part of their official duties; on a worldwide, perpetual, irrevocable, royalty-free basis to BMJ Publishing Group Ltd ("BMJ") its licensees and where the relevant Journal is co-owned by BMJ to the co-owners of the Journal, to publish the Work in this journal and any other BMJ products and to exploit all rights, as set out in our licence.

The Submitting Author accepts and understands that any supply made under these terms is made by BMJ to the Submitting Author unless you are acting as an employee on behalf of your employer or a postgraduate student of an affiliated institution which is paying any applicable article publishing charge ("APC") for Open Access articles. Where the Submitting Author wishes to make the Work available on an Open Access basis (and intends to pay the relevant APC), the terms of reuse of such Open Access shall be governed by a Creative Commons licence – details of these licences and which Creative Commons licence will apply to this Work are set out in our licence referred to above.

Other than as permitted in any relevant BMJ Author's Self Archiving Policies, I confirm this Work has not been accepted for publication elsewhere, is not being considered for publication elsewhere and does not duplicate material already published. I confirm all authors consent to publication of this Work and authorise the granting of this licence.

For peer review only

21Capacity and quality of maternal and child health services delivery at the subnational primary health care level in relation to intermediate health outputs: a cross-sectional study of 12 low-income and middle-income countries

Authors and affiliations

Marwa Ramadan[1]*, Jose Carlos Gutierrez[1], Cameron Feil[1], Sarah Bolongaita[1], Oscar Bernal[1], Manuela Villar Uribe[1]

[1]Health Nutrition and Population Global Practice, World Bank Group, Washington, DC, USA.

***Corresponding Author**

Marwa Ramadan

Health Nutrition and Population Global Practice, World Bank Group, Washington, DC, USA

mramadan@worldbank.org

Keywords

Primary healthcare; healthcare quality; measurement; performance assessment; subnational; low- and middle-income countries

For peer review only

Abstract

Objectives: To examine the capacity and quality of Maternal and Child Health (MCH) services at the subnational Primary Health Care (PHC) level in 12 Low-and Middle-Income Countries (LMICs) and its association with intermediate health outputs such as coverage and access to care.

Design: Observational cross-sectional study using matched subnational data from service provision assessment surveys and demographic health surveys from 2007-2019.

Settings: 138 sub-national areas with available survey data in 12 LMICs (Afghanistan, Bangladesh, Democratic Republic of Congo, Haiti, Kenya, Malawi, Namibia, Nepal, Rwanda, Senegal, Tanzania, Uganda).

Outcomes: Eight intermediate MCH outcomes/outputs were explored: 1) met need for family planning by modern methods; 2) attendance of four or more antenatal care visits; 3) perceived financial barriers to care; 4) perceived geographic barriers to care; 5) Diphtheria-Pertussis- Tetanus (DPT) third dose coverage; 6) DPT dropout-rate; 7) care seeking for pneumonia; and 8) oral rehydration solutions coverage.

Results: Overall, moderate to poor PHC performance was observed across the 12 countries, with substantial heterogeneity between the different subnational areas in the same country as well as within the same subnational area across both capacity and quality subdomains. The analysis of the relationship between PHC service delivery and child health outcomes revealed that recent supervision [b=0.34, p<0.01] and supervisors' feedback [b=0.28, p<0.05] were each associated with increased care seeking for pneumonia. We also observed the associations of several measures of capacity and quality with DPT immunization. The analysis of maternal health outcomes yielded only a few statistically significant results at p<0.05 level, however, none remained significant after adjusting for other covariates.

Conclusion: The results of this analysis illustrate the heterogeneity in the capacity and quality of PHC service delivery within LMICs. Countries seeking to strengthen their PHC systems could improve PHC monitoring at the subnational level to better understand subnational bottlenecks in service delivery.

For peer review only - http://bmjopen.bmj.com/site/about/guidelines.xhtml

Strengths and limitations of this study:

- Rather than focusing on Primary Healthcare (PHC) delivery at the national level, this study bridges a research gap by examining PHC service delivery at the subnational level and its association with improved health outputs in 12 low-and middle-income countries.
- The methodology utilized in the present study demonstrates how publicly available survey data such as the Demographic Health Survey (DHS) and the Service Provision Assessment (SPA) survey can be valuable tools in highlighting bottlenecks and disparities in PHC service delivery at the subnational level.
- As the study is observational and cross-sectional, the association presented should not be interpreted as causal given the potential for an ecological bias.
- Although the sample was constructed such that the time between the SPA and DHS surveys in each country was less than 5 years, it is possible that changes in time-varying factors could have influenced the outcomes under study.
- There is a potential for omitted variable bias through other factors that may influence the capacity, quality, and performance variables under study but are unmeasured in the SPA and DHS surveys.

For peer review only

Introduction

Despite renewed global commitment to Primary Healthcare (PHC) in recent years, the measurement of PHC capacity and quality remains a challenge for policymakers seeking to improve the performance of health services. The transition from the Millennium Development Goals (MDGs) to Sustainable Development Goals (SDGs) in 2016 marked a significant shift in the global health agenda. SDG 3, "Achieving Universal Healthcare Coverage," was a call to action to strengthen health systems, shifting the global health agenda away from vertically planned health programs and initiatives. In 2018, forty years after the Alma-Alta Declaration which identified PHC as the pathway to achieving health for all, global actors, governments, and organizations re-affirmed a global commitment to PHC with the issuance of the Astana Declaration (1).

More significant efforts are required to improve primary health care performance in Low-and-Middle-income countries (LMICS) (2). Performance measurement has the potential to catalyze PHC improvement by identifying bottlenecks (3,4). The Primary Health Care Performance Initiative (PHCPI), a global collaborative dedicated to measuring and improving PHC systems in LMICs, developed a conceptual framework illustrating the relationships between financing, inputs, and core functions of the PHC system (2). In 2018, PHCPI operationalized the Conceptual Framework into the Vital Signs Profile, which uses qualitative and quantitative indicators to provide a comprehensive understanding of PHC performance in a county (3).

The relationship between capacity, quality and health system performance has long been studied. Donabedian captured the causal chain of inputs to outputs, commonly defined as the 'input-process-output-model', in 1966. More recent efforts by Randhawa (4) and Veillard et al.(5) have demonstrated the relationship between inputs and outcomes of PHC system. Previous studies also highlighted that PHC capacity, including the availability of providers, governance, and financing, has been found to impact PHC-related outcomes in LMICs at the national level (6–10). In addition, several studies have identified relationships between elements of high-quality PHC and performance at the national level (2,11–14). However, the majority of associations were explored at the national level and significant gaps still exist in understanding PHC service delivery at the sub-national level and its association with improved health outputs.

This paper aims to fill some of the current measurement gaps by exploring the relationship between PHC system capacity, quality, and performance at the sub-national level using quantitative indicators from the PHCPI Vital Signs Profile. Specifically, we assess PHC service delivery at both the national and subnational levels in 12 LMICs. In addition, we examine the association of PHC capacity and quality with intermediate health outputs such as coverage and access to services at the subnational level.

Methods

<u>Data sources</u>

In this cross-sectional analysis, Service Provision Assessment (SPA) surveys and Demographic Health Surveys (DHS) were the main sources of data on PHC service delivery and PHC intermediate population health outputs, respectively in 12 LMICS (Afghanistan, Bangladesh, DRC (democratic republic of Congo), Haiti, Kenya, Malawi, Namibia, Nepal, Rwanda, Senegal, Tanzania, Uganda). Specifically, we included all LMICS (as defined by the world bank income classification) where both a DHS and a SPA survey were conducted within a 5-year interval in the period between 2007 and 2019. SPA surveys are comprehensive facility assessments that aim to collect information on the availability of services at the

facility level, the readiness of facilities to provide these services, the compliance with acceptable standards of care, and whether clients and service providers are satisfied with the service delivery process (15). SPA surveys usually involve random selection of 400-700 facilities from a comprehensive sampling list of all public and private health facilities in a country or alternatively a complete census of facilities in the country. Facilities are usually categorized by facility type, managing authority (public and non-public), as well as subnational region; sampling and sample size ensure representativeness across each of these categories. Weights are applied during the analysis of these surveys to ensure that the contribution of selected facilities is proportional to their distribution in the country. On the other hand, DHS are nationally representative household surveys and a vital source of information on population, health, and nutrition indicators in more than 90 countries. The standard DHS survey is conducted every five years using a large sample size (5000-30,000 households per survey), and usually utilizes a two-stage sampling methodology based on the availability of census information (16). The DHS sampling and sample size ensure representativeness at the subnational region level. Table 1 provides additional information on the sampling structure and the timeframe for data collection in each of the studied countries. The SPA and DHS datasets utilized in this study are publicly available and can be downloaded from the DHS website (17).

Table 1: Characteristics of DHS and SPA surveys included in the analysis

**Country**	**Sub-national areas**	**DHS Field Work**	**DHS sampling**	**DHS sample size (ever-married Women)**	**Year data collection -SPA**	**SPA sample Size (Facilities)**
**Afghanistan**	34 provinces	June 2015 - February 2016	two-stage stratified sample	24,395	November 2018- January 2019	160 facilities**
**Bangladesh**	8 divisions	October 24, 2017- March 2018	two-stage stratified sample	20,100	July 2017-to October 2017	1,600
**DRC**	26 new provinces	August 2013 - February 2014	Three-stage stratified sample*	18,827	October 2017 - April 2018	1380
**Haiti**	10 departments	November 2016 - April 2017	two-stage stratified sample	14,371	December 2017 - May 2018	1007
**Kenya**	8 regions	May 2014 - October 2014	two-stage stratified sample	31,079	January 2010- May 2010	695
**Malawi**	3 regions	October 2015 - February 2016	two-stage stratified sample	24562	June 2013- February 2014	1060
**Namibia**	13 regions	May 2013 - September 2013	two-stage stratified sample	9176	July 2009- October 2009	446
**Nepal**	7 provinces	June 2016 - January 2017	Stratified sample (two stages in rural areas and three stages in urban areas)	12862	April 2015- November 2015	1,000

For peer review only - http://bmjopen.bmj.com/site/about/guidelines.xhtml

**Rwanda**	5 provinces	September 2010 - March 2011	two-stage stratified sample	13671	June 2007-August 2007	538
**Senegal**	14 regions	April 2017 - December 2017	two-stage stratified sample	16787	March 2017-December 2017	3764
**Tanzania**	25 regions	August 2015 - February 2016	two-stage stratified sample	13266	October 2014 – March 2015	1,200
**Uganda**	15 regions	June 2011 - December 2011	two-stage stratified sample	8674	July-October 2007	491

*Two-stage stratified sample in statutory towns and cities of established provinces, three-stage stratified sample in rest of established provinces and new provinces, **Sampling limited to urban areas of seven major provinces (Kabul, Nangarhar, Paktya, Kunduz, Balkh, Kandahar, and Herat)

For peer review only

Metrics

Guided by the PHCPI framework and survey data availability, the capacity of the PHC system was assessed using the following five indicators: 1) availability of basic facility Infrastructure; 2) the presence of recent supervision within the past 6 months; 3) the provision of feedback by supervisors; 4) the provision of outreach services; and 5) absence of PHC fees defined as free immunization, RMNCH, non-communicable diseases and HIV services. Similarly, whenever data was available, PHCPI's Vital Signs Profile (VSP) guided the assessment of PHC quality of care through the following six indicators: 1) waiting time of less than 60 minutes; 2) availability of providers defined as an average consultation time of more than 10 minutes; 3) competence of providers defined as the average of antenatal care, family planning and sick child quality scores based on WHO guidelines; 4) person-centeredness defined as the percent of clients who were told their diagnosis; 5) safety defined as the presence of adequate infection control practices and waste disposal at the facility; and 6) comprehensiveness of PHC services defined as the average availability of tracer items for RMNCH, infectious diseases and non-communicable diseases. Additional information on PHCPI's framework and methodology is provided in an earlier publication (5).

Additionally, eight intermediate maternal and child health outcomes/outputs were explored in accordance with the PHCPI vital signs profile and consistent reporting of DHS indicators in the studied countries. Specifically, we assessed the following maternal and child health outputs: 1) met need for family planning by modern methods; 2) attendance of four or more antenatal care visits; 3) perceived financial barriers to care; 4) perceived geographic barriers to care; 5) DPT3 coverage; 6) DPT dropout-rate; 7) care seeking for pneumonia; and 8) Oral Rehydration Solutions (ORS) coverage. Other PHC-related outputs (e.g., non-communicable diseases, tuberculosis, and HIV) were not assessed due to the limited availability of sub-national data in the studied countries. Detailed definition of each of the capacity, quality and output indicators are provided in Supplementary Table 1 [See Supplementary Material]

For peer review only - http://bmjopen.bmj.com/site/about/guidelines.xhtml

Data analysis

Population and health facility data were linked at the first administrative level (e.g., region, province…etc.) whenever data was available and the gap between a population-based survey and a health facility survey was less than five years. In situations where substantial variation existed at the first administrative level between the two types of surveys, geographic coordinates of health facilities and DHS clusters were input into geographic information system (GIS) software (ArcGIS) for matching. Specifically, in the Democratic Republic of Congo, DHS data in 2013 were reported for 11 formerly defined provinces, while 2017 SPA data were reported for 26 newly defined provinces. ArcGIS was used to match subnational areas in both types of surveys. In addition, in Afghanistan, the SPA survey was only conducted in seven urban subnational areas, so the match process with DHS was limited to these areas [See Supplementary Figure 1 in Supplementary Material]

To compute subnational estimates, we applied the recommended SPA weights at the facility, provider, and visit levels in the 12 studied countries. We then computed national averages based on the assumption that all subnational areas were equally weighted in each of the studied countries. In addition, we examined the subnational association between the computed service delivery sub-domains and DHS intermediate maternal and child health outputs using bivariate and multivariable models with a significance level at $p<0.05$. Given the hierarchical nature of the examined population and health facility data, we utilized a two-level fixed-effects model to account for possible clustering of the observations at the country level. We also adjusted for the following population and facility characteristics at the subnational level: proportion of urban households, proportion of population in the 5th economic quintile (highest), and proportion of women with secondary or more education, proportion of private facilities (per the total number of health facilities at the subnational level), proportion of hospitals (per the total number of health facilities at the subnational level),. The statistical analyses were all performed in STATA (version 17.0), tables were created using Microsoft Excel.

Patient and public involvement

No patient involvement

Results

I. PHC service delivery at the national and subnational levels

In the present study, the capacity and quality of PHC service delivery were analyzed in 138 subnational areas in 12 LMICs. Substantial heterogeneity was observed across countries in the capacity subdomain. For example, the average availability of facility infrastructure (basic amenities and infection prevention) was as high as 80% in Afghanistan and as low as 48 % in DRC. Most facilities in the analyzed subnational areas reported the presence of recent supervision, with an average ranging from 94% in Bangladesh to 70% in Tanzania, while supervisor’s feedback was provided in a relatively fewer number of facilities, with the highest average reported in Bangladesh (91%) and the lowest in Afghanistan (57 %). Meanwhile, the provision of facility outreach services varied largely across the studied countries, with 84 % of facilities in Nepal providing outreach services versus only 14 % of facilities in the analyzed subnational areas in Afghanistan. Similarly, large variation in charging PHC fees was observed across the sample; 99% of facilities in the analyzed subnational areas of Uganda did not charge fees for PHC services versus only 32 % in Tanzania. Table 2 shows the average score of individual capacity sub-domains in the analyzed subnational areas across the 12 studied countries.

For peer review only

For peer review only - http://bmjopen.bmj.com/site/about/guidelines.xhtml

When the quality-of-service delivery was assessed, most countries had moderate to poor scores in all quality sub-domains except waiting time and comprehensiveness, where some countries performed well. As with capacity, cross-country variation in quality sub-domains was observed. For example, the average waiting time was less than 60 minutes in more than 90 % of the facilities in the analyzed subnational areas of Afghanistan and Nepal versus only 37 % of the analyzed facilities in Rwanda. However, the average consultation time was more than 10 minutes in only 38 % of the analyzed Afghan facilities. Competence of providers was one of the quality sub-domains with the lowest average scores across the studied countries ranging from 30 % on average, for the analyzed facilities in Afghanistan to 54 % in Namibia. Similarly, person centeredness scored poorly in most countries with an average score as low as 9 % for the analyzed facilities in Senegal versus 54 % in Uganda. Meanwhile, at least two thirds of the analyzed facilities offered comprehensive PHC services including RMNCH, non-communicable and infectious disease services in all the studied countries (Table 2).

Table 2: The capacity and quality of PHC service delivery in the studied countries (average scores of subnational areas)

Domain	Subdomain	Country											
		AF18*	BD17	DRC18	HT17	KE10	MW13	NM09	NP15	RW07	SN17	TZ15	UG07
Capacity	Facility Infrastructure	80%	51%	48%	62%	66%	67%	69%	53%	63%	62%	56%	55%
	Recent Supervision	77%	94%	77%	74%	74%	74%	72%	72%	82%	77%	70%	81%
	Supervisor Feedback	57%	91%	72%	64%	63%	65%	61%	64%	74%	76%	65%	62%
	Outreach Services	14%	18%	73%	39%	43%	70%	20%	84%	79%	48%	50%	79%
	Not charging PHC Fees	50%	67%	42%	73%	67%	74%	94%	92%	96%	65%	32%	99%
	**Capacity score**	55%	65%	61%	60%	61%	70%	63%	73%	79%	64%	53%	76%
Quality	Waiting Time < 60 minutes	92%	-	76%	58%	61%	48%	44%	94%	37%	77%	57%	49%
	Provider Availability	38%	-	78%	65%	63%	41%	63%	44%	39%	62%	70%	42%
	Provider Competence	30%	-	39%	40%	41%	37%	54%	31%	41%	40%	40%	49%
	Person Centeredness	45%	-	31%	25%	38%	45%	54%	33%	26%	9%	46%	54%
	Safety	31%	39%	32%	55%	65%	49%	73%	45%	26%	65%	41%	26%
	Comprehensiveness	68%	63%	74%	79%	81%	75%	85%	76%	75%	65%	71%	72%
	**Quality**	50%	51%	55%	56%	61%	49%	62%	54%	41%	53%	54%	47%

AF18: Afghanistan 2018, BD17: Bangladesh 2017, DRC18: democratic republic of Congo 2018, HT17: Haiti 2017, KE10: Kenya 2010, MW13: Malawi 2013, NM09: Namibia 2009, NP15: Nepal 2015, RW07: Rwanda 2007, SN17: Senegal 2017, TZ15: Tanzania 2015, UG07: Uganda 2007
* Estimates represent the average of urban subnational data for which SPA and DHS data were available

Subnational analysis of service delivery sub-domains revealed the presence of geographic disparities at the first administrative level in both PHC capacity and quality sub-domains. For example, large subnational variation was observed in the average availability of facility infrastructure in Senegal,

For peer review only - http://bmjopen.bmj.com/site/about/guidelines.xhtml

ranging from 83 % in Diourbel region to 45 % in Kedougou region. Similarly, considerable subnational variation was observed in the provision of outreach services in Haiti, ranging from 45 % in North-East to 5 % in North-West department. Charging of PHC fees in DRC was also another example of administrative variation in PHC capacity, where 81 % of facilities in Sankuru province provided free PHC services versus none of the facilities in North-Kivu or South-Kivu provinces. Similarly, subnational variation was observed in most of the examined quality sub-domains. For example, the range of variation across the different subnational areas in Tanzania was more than 60 % points for waiting time. In addition, the range of variation was more than 50 % for availability of providers across the different subnational areas in Senegal. Furthermore, person centered care ranged from 89 % in Kunduz province to only 1 % in Kandahar province in Afghanistan. Meanwhile, the lowest subnational variations were observed in provider competence sub-domain, where the majority of subnational areas scored below 50 % in the 12 studied countries.

Not only did we observe variations within the same country across different subnational areas, but also within the same subnational area across both quality and capacity sub-domains. For example, the range of variation across different capacity sub-domains was more than 50 % points in the majority of the analyzed sub-national areas in Afghanistan, Bangladesh, and Namibia. Most of these variations were derived by the difference in the capacity of the facilities in providing outreach services and offering free PHC services. Similarly, several subnational areas in Afghanistan, DRC and Senegal showed substantial variation across the different quality sub-domains. For example, the range of variation across quality sub-domains in Kandahar, Afghanistan was 99 %, where all facilities had a waiting time of less than 60 minutes; however only 1 % provided person centered care. Supplementary Table 2 and Supplementary Table 3 provide detailed information on the capacity and quality scores in each of the analyzed 138 subnational areas [See Supplementary Material]

II. The association between PHC service delivery sub-domains and intermediate health outputs

Examining the relationship between subnational PHC capacity and maternal health outputs, no statistically significant association was found between the capacity of facilities and antenatal care coverage after adjustment for subnational characteristics. Meanwhile, a statistically significant association was observed between the general availability of basic facility infrastructure and perceived economic and geographic barriers to care. Specifically, a 10 %-point increase in the average availability of basic infrastructure at the subnational level was associated with 3 % lower perceived financial barriers to care [b = -0.35, $p < 0.05$] and 4 % lower perceived geographic barriers to care [b = -0.39, $p < 0.05$]. However, the associations were not statistically significant after adjustment for subnational facility and population characteristics (Table 3). Coverage of family planning services was also negatively associated with the provision of outreach services. However, this paradoxical association was no longer significant in the multivariable specification. Meanwhile, no statistically significant association was found between any of the examined PHC quality sub-domains and maternal health outputs (Table 3).

Table 3: The association between PHC service delivery subdomains and maternal health outputs at the subnational level

Sub-domain			Antenatal		Family planning		Cost Barrier		Geographic Barrier	
			Bivariate	Multivariable	Bivariate	Multivariable	Bivariate	Multivariable	Bivariate	Multivariable
Capacity	Facility Infrastructure	β	0.23	0.11	0.29	0.16	-0.35*	-0.23	-0.39*	-0.16
		SE	0.13	0.13	0.16	0.19	0.15	0.14	0.15	0.16
	Supervision in the past	β	-0.09	0.02	-0.12	0.01	0.05	-0.02	0.00	-0.14

For peer review only - http://bmjopen.bmj.com/site/about/guidelines.xhtml

	6 months	SE	0.08	0.08	0.09	0.10	0.09	0.08	0.10	0.09
	Supervisor Feedback	β	-0.06	0.01	-0.06	0.03	-0.03	-0.09	-0.02	-0.11
		SE	0.08	0.07	0.09	0.09	0.09	0.07	0.09	0.08
	Outreach Services	β	-0.07	0.00	-0.13*	-0.09	0.04	-0.01	0.09	0.04
		SE	0.05	0.04	0.06	0.06	0.06	0.05	0.06	0.05
	Not charging PHC fees	β	0.01	0.01	-0.01	0.00	-0.02	0.00	0.02	0.06
		SE	0.05	0.04	0.06	0.06	0.06	0.05	0.06	0.05
**Quality**	Waiting time < 60 minutes	β	-0.01	0.01	-0.11	-0.10	0.09	0.05	0.11	0.07
		SE	0.06	0.05	0.08	0.07	0.07	0.06	0.08	0.06
	Provider Competence	β	0.07	0.00	0.04	0.01	-0.28	-0.10	-0.26	-0.10
		SE	0.15	0.13	0.19	0.18	0.19	0.14	0.19	0.15
	Provider Availability	β	-0.04	-0.08	-0.16	-0.15	-0.11	-0.13	0.02	0.03
		SE	0.09	0.07	0.11	0.10	0.10	0.08	0.11	0.08
	Person centeredness	β	-0.01	-0.04	0.01	-0.02	-0.06	-0.01	-0.02	0.02
		SE	0.05	0.04	0.06	0.06	0.06	0.05	0.07	0.05
	Safety	β	0.09	0.06	0.01	-0.03	0.20	0.13	0.11	0.10
		SE	0.08	0.07	0.11	0.11	0.11	0.08	0.11	0.09
	Comprehensiveness	β	0.09	0.09	-0.05	-0.02	-0.20	-0.09	-0.14	-0.05
		SE	0.10	0.08	0.11	0.11	0.11	0.08	0.12	0.09

* p<0.05

In multivariable models, results were adjusted for proportion of private facilities, hospitals, urban households, population in the 5th economic quintile and women with secondary education or more at the sub-national level

Afghanistan was excluded from the analysis for sub-national sample limitation

On the other hand, PHC service delivery sub-domains were more likely to be statistically significantly associated with child health outputs. For example, a 10 %-point increase in the availability of basic facility infrastructure was associated with an increase of 5 % in DPT3 immunization coverage [b =0.45, p<0.05] and a decrease of 3% in DPT-dropout rates [b = - 0.30, p<0.05]. Additionally, recent facility supervision [b=0.34, p<0.01] and provision of feedback by supervisors [b=0.28, p<0.05] were each associated with increased care seeking for pneumonia after adjustment for facility and population characteristics. Surprisingly, significantly lower immunization coverage and higher dropout rates were observed in subnational areas where facilities did not charge PHC fees, even after adjustment for facility and population characteristics (Table 4). Meanwhile, of the examined quality sub-domains, only comprehensiveness showed a statistically significant association with subnational DPT3 immunization coverage and lower drop-out rates. Specifically, a 10 %-point increase in the comprehensiveness of PHC services was associated with 2 % improvement in DPT3 coverage and 2 % lower DPT-dropout rates at the subnational level [b= 0.22, p<0.05 and b=-0.21, p<0.01 respectively] (Table 4).

<u>Table 4: The association between PHC service delivery subdomains and child health outputs at the subnational level</u>

**Sub-domain**			**DPT3**		**DPT_ dropout**		**Pneumonia care seeking**		**ORS coverage**	
			Bivariate	Multivariable	Bivariate	Multivariable	Bivariate	Multivariable	Bivariate	Multivariable
**Capa**	Facility Infrastructure	β	0.54**	0.45*	-0.37**	-0.30*	0.18	0.23	-0.02	0.01
		SE	0.15	0.18	0.12	0.14	0.18	0.22	0.12	0.15
	Supervision in the past 6 months	β	-0.04	0.08	0.03	-0.07	0.23*	0.34**	0.08	0.08
		SE	0.09	0.10	0.07	0.08	0.11	0.12	0.07	0.08
	Supervisor	β	-0.03	0.05	0.01	-0.05	0.22*	0.28**	0.10	0.09

For peer review only - http://bmjopen.bmj.com/site/about/guidelines.xhtml

	Feedback	SE	0.09	0.09	0.07	0.07	0.10	0.11	0.07	0.07
	Outreach Services	β	-0.07	-0.02	0.06	0.01	-0.04	-0.05	0.05	0.04
		SE	0.06	0.06	0.04	0.05	0.07	0.07	0.05	0.05
	Not charging PHC fees	β	-0.14*	-0.17**	0.07	0.09	0.09	0.09	0.08	0.07
		SE	0.06	0.06	0.05	0.05	0.07	0.07	0.05	0.05
**Quality**	Waiting time < 60 minutes	β	-0.12	-0.11	0.08	0.07	0.06	0.06	-0.03	-0.03
		SE	0.07	0.07	0.06	0.05	0.08	0.08	0.06	0.06
	Provider Competence	β	0.09	0.03	-0.06	-0.02	0.13	0.14	-0.22	-0.19
		SE	0.19	0.18	0.14	0.14	0.21	0.21	0.14	0.14
	Provider Availability	β	-0.18	-0.17	0.13	0.12	0.11	0.11	-0.02	-0.06
		SE	0.10	0.10	0.08	0.08	0.12	0.12	0.08	0.08
	Person centeredness	β	-0.08	-0.10	0.06	0.08	0.04	0.03	-0.01	-0.02
		SE	0.06	0.06	0.05	0.05	0.07	0.07	0.05	0.05
	Safety	β	0.01	0.03	-0.03	-0.04	0.07	0.07	-0.09	-0.07
		SE	0.11	0.11	0.08	0.08	0.12	0.13	0.09	0.09
	Comprehensiveness	β	0.23*	0.22*	-0.22**	-0.21**	-0.10	-0.11	0.03	0.03
		SE	0.11	0.10	0.08	0.08	0.13	0.13	0.09	0.08

Standard errors in parentheses
* p<0.05, ** p<0.01
In multivariable models, results were adjusted for proportion of private facilities, hospitals, urban households, population in the 5th economic quintile and women with secondary education or more at the subnational level
Afghanistan was excluded from the analysis for subnational sample limitation

Discussion

Overall, the results of the descriptive analysis illustrate the heterogeneity in the capacity and quality of PHC service delivery within LMICs. The two-level analysis of maternal health outcomes yielded only a few statistically significant results at the p<0.05 level, however, none remained significant after adjusting for other covariates. This suggests that factors outside health facilities' control may play a greater role in determining mothers' care seeking decisions for antenatal care and family planning, and in shaping perceptions of cost and geographic barriers to care. For example, individual- and household-level factors such as the mother's education and socioeconomic status are associated with the use of family planning and antenatal care services. Nonetheless, supply-side constraints at the health system level such as supply chain challenges may also contribute to stockouts of essential FP commodities (18). Analysis at levels more granular than the subnational area (the first administrative level) may yield further insight into the relationship of supply-side PHC characteristics and maternal health outcomes.

Meanwhile, the two-level analysis of child health outcomes revealed significant associations between supervision and care-seeking for pneumonia, as well as associations of several measures of capacity and quality with DPT3 immunization. Supervision in the last 6 months and supervisor feedback are considered measures of PHC capacity in the PHCPI framework, and it is hypothesized that measures of capacity operate through improved quality of health services. In the case of care-seeking for pneumonia, it is possible that facilities with greater capacity and implementation of supervision deliver care perceived to be of higher quality, thus increasing mothers' likelihood of seeking healthcare for their child at the facility. One review finds inconclusive but promising evidence that supportive supervision can improve quality of care, noting that supervision alone is insufficient; health workers require training and the availability of medicines, diagnostics and equipment to provide quality care (19). Patient perceptions of a facility's quality of care also influence utilization and care seeking (20–26). There is also ample literature demonstrating the influence of various household and individual-level factors such as

For peer review only - http://bmjopen.bmj.com/site/about/guidelines.xhtml

cultural beliefs, disease severity, geographic location, wealth, education, and socioeconomic status on care-seeking behavior (27,28)

Finally, our results found several associations with DPT3 immunization and dropout that generally align with existing literature (facility infrastructure and comprehensiveness) and in the case of user fees, counter to what might be expected. In line with our findings, prior research in Haiti found that both infrastructure quality and the quality-of-service delivery were associated with increased utilization of primary care services, including antenatal care and vaccination (29). Similarly, a study in rural India found that children in districts where more villages had a healthcare subcenter were more likely to receive at least one dose of DPT3, though no association with DPT3 dropout rates was observed (30). The results above also suggest that comprehensiveness, a measure of PHC quality, is also associated with better immunization outcomes. This is in line with prior studies documenting that children of mothers who are counseled during vaccination sessions are more likely to complete all three DPT doses (25).

One possible explanation behind the counterintuitive negative association between the absence of user fees and DPT3 immunization is that the estimated association may be confounded by other factors determining both user fee exemptions and immunization coverage. For example, subnational areas that are generally poorer and have greater barriers to care may be more likely adopt policies exempting user fees, yet those characteristics may also result in lower immunization rates. A second explanation under the interpretation that the observed association is unconfounded may be related to potential unintended consequences of user fee removal. While the benefits of removing user fees are well documented in boosting health service utilization (31–33), some have also expressed concerns that the process of user fee removal must be designed carefully such that facilities are able to adapt. The elimination of fees and reduction of fees without a corresponding increase in resources from government sources could unintentionally lead to increased pressure on human resources and the availability of essential commodities, including vaccines. Further, increased demand for curative services without an increase in the health workforce could result in curative services "crowding out" preventative services such as vaccination. Ultimately, further research into any potentially paradoxical relationship between user fees and DPT3 vaccination.

The study should also be interpreted in light of its limitations. As the study is observational and cross-sectional, the association presented should not be interpreted as causal effects. Using data aggregated at the subnational level also entails the potential for an ecological fallacy and requires caution in interpretation. In addition, while the sample was constructed such that the time between the SPA and DHS surveys in each country was less than 5 years, it is possible that changes in time-varying factors or other events in that interlude could influence the outcomes under study. Afghanistan's more limited sample size restricted to predominantly urban areas presents an additional limitation, therefore it was excluded from regression analysis to mitigate any potential confounding. Finally, though we control for selected characteristics at the subnational and individual levels, there is a potential for omitted variable bias through other factors that may influence the capacity, quality, and performance variables under study but are unmeasured in the SPA and DHS surveys. Ultimately, further research into the relationships between PHC capacity, quality and service delivery outcomes could adopt other methodologies to establish potential causal links. Taking advantage of repeated survey waves may provide one such strategy to track how these indicators may have changed over time and to what extent improvements in capacity and quality lead to better results.

Our findings have important policy implications. The heterogenicity observed in PHC service delivery within and between subnational areas highlight that there is not one solution that fits all. Countries and

For peer review only

For peer review only - http://bmjopen.bmj.com/site/about/guidelines.xhtml

stakeholders seeking to strengthen their PHC systems could improve PHC monitoring at the subnational level. Disaggregation of national indicators by geographic regions and adoption of subnational scorecards can potentially unmask real bottlenecks that hinder the overall country progress. In addition, a participatory and mixed-methods approach to monitoring is important to better understand the diverse challenges across subnational areas and develop tailored approaches to resolve the problems identified.

Conclusion

Substantial heterogeneity and disparities were observed across the 12 studied LMICS, as well as between and within subnational areas in both the capacity and quality subdomains. Countries seeking to strengthen their PHC systems could improve PHC monitoring at the subnational level to better understand the diverse challenges across subnational areas and develop tailored approaches to resolve the problems identified. The associations observed between capacity, quality and performance also provide potential avenues for further research and contribute to the literature on the determinants of care-seeking behavior and immunization.

Ethics statements

This study was based on secondary analysis of anonymized publicly available datasets. No ethics approval, or patient consent was need for the present analysis.

Data availability statement

The original datasets analyzed during the current study are available from the DHS program website, https://dhsprogram.com/data/available-datasets.cfm. The analytical dataset generated during the current study is available from the corresponding author on reasonable request

Contributors : MR and MVU were responsible for the conception of this study and for methods development. MR analyzed data. MR, JCG, CF prepared the first draft. All authors (MR, JCG, CF, SB, OB, MVU) reviewed results or reviewed and contributed to the final manuscript.

Funding: This research was supported, in whole or in part, by the Bill and Melinda Gates Foundation (Grant number INV000932), and it had no role in study design, data collection, data analysis, data interpretation or writing of the manuscript.

Competing interests: None declared.

Reference

1. World Health Organization, Organisation for Economic Co-operation and Development, International Bank for Reconstruction and Development. Delivering quality health services: a global imperative for universal health coverage [Internet]. Geneva: World Health Organization; 2018. Available from: https://apps.who.int/iris/handle/10665/272465

2. Bitton A, Fifield J, Ratcliffe H, Karlage A, Wang H, Veillard JH, et al. Primary healthcare system performance in low-income and middle-income countries: a scoping review of the evidence from 2010 to 2017. BMJ Glob Health. 2019 Aug 16;4(Suppl 8):e001551.

For peer review only - http://bmjopen.bmj.com/site/about/guidelines.xhtml

3. Vital Signs Profiles | PHCPI [Internet]. [cited 2022 May 18]. Available from: https://improvingphc.org/vital-signs-profiles

4. Randhawa H. Report of the Expert Consultation on Primary Care Systems Profiles & Performance (PRIMASYS). :35.

5. Veillard J, Cowling K, Bitton A, Ratcliffe H, Kimball M, Barkley S, et al. Better Measurement for Performance Improvement in Low- and Middle-Income Countries: The Primary Health Care Performance Initiative (PHCPI) Experience of Conceptual Framework Development and Indicator Selection. Milbank Q. 2017 Dec;95(4):836–83.

6. Nikoloski Z, Albala S, Montero AM, Mossialos E. The impact of primary health care and specialist physician supply on amenable mortality in Mexico (2000-2015): Panel data analysis using system-Generalized Method of Moments. Soc Sci Med. 2021 Jun;278:113937.

7. Schneider MT, Chang AY, Crosby SW, Gloyd S, Harle AC, Lim S, et al. Trends and outcomes in primary health care expenditures in low-income and middle-income countries, 2000–2017. BMJ Glob Health. 2021 Aug;6(8):e005798.

8. Basu S, Berkowitz SA, Phillips RL, Bitton A, Landon BE, Phillips RS. Association of Primary Care Physician Supply With Population Mortality in the United States, 2005-2015. JAMA Intern Med. 2019 Apr 1;179(4):506–14.

9. Hone T, Rasella D, Barreto M, Atun R, Majeed A, Millett C. Large Reductions In Amenable Mortality Associated With Brazil's Primary Care Expansion And Strong Health Governance. Health Aff (Millwood). 2017 Jan 1;36(1):149–58.

10. Russo LX, Scott A, Sivey P, Dias J. Primary care physicians and infant mortality: Evidence from Brazil. PLOS ONE. 2019 May 31;14(5):e0217614.

11. Ranabhat CL, Jakovljevic M, Dhimal M, Kim CB. Structural Factors Responsible for Universal Health Coverage in Low- and Middle-Income Countries: Results From 118 Countries. Frontiers in Public Health [Internet]. 2020 [cited 2022 Mar 14];7. Available from: https://www.frontiersin.org/article/10.3389/fpubh.2019.00414

12. Lozano R, Fullman N, Mumford JE, Knight M, Barthelemy CM, Abbafati C, et al. Measuring universal health coverage based on an index of effective coverage of health services in 204 countries and territories, 1990–2019: a systematic analysis for the Global Burden of Disease Study 2019. The Lancet. 2020 Oct 17;396(10258):1250–84.

13. Leslie HH, Malata A, Ndiaye Y, Kruk ME. Effective coverage of primary care services in eight high-mortality countries. BMJ Glob Health. 2017 Sep 4;2(3):e000424.

14. Nguhiu PK, Barasa EW, Chuma J. Determining the effective coverage of maternal and child health services in Kenya, using demographic and health survey data sets: tracking progress towards universal health coverage. Trop Med Int Health. 2017 Apr;22(4):442–53.

15. USAID. The DHS Program - Service Provision Assessments (SPA) [Internet]. Demographic Health Surveys. 2019 [cited 2020 Nov 28]. p. 1–2. Available from: https://dhsprogram.com/What-We-Do/Survey-Types/SPA.cfm

For peer review only

For peer review only - http://bmjopen.bmj.com/site/about/guidelines.xhtml

16. Croft TN, Marshall AM, Allen CK, Arnold F, Assaf S, Balian S. Guide to DHS statistics. Rockville: ICF. 2018;645.

[Dataset]17. The DHS Program - Available Datasets [Internet]. [cited 2022 Sep 19]. Available from: https://dhsprogram.com/data/available-datasets.cfm

18. Wulifan JK, Brenner S, Jahn A, De Allegri M. A scoping review on determinants of unmet need for family planning among women of reproductive age in low and middle income countries. BMC Womens Health. 2016 Jan 15;16:2.

19. Bailey C, Blake C, Schriver M, Cubaka VK, Thomas T, Martin Hilber A. A systematic review of supportive supervision as a strategy to improve primary healthcare services in Sub-Saharan Africa. International Journal of Gynecology & Obstetrics. 2016 Jan 1;132(1):117–25.

20. Rao KD, Sheffel A. Quality of clinical care and bypassing of primary health centers in India. Soc Sci Med. 2018 Jun;207:80–8.

21. Gauthier B, Wane W. Bypassing health providers: The quest for better price and quality of health care in Chad. Social Science & Medicine. 2011 Aug 1;73(4):540–9.

22. Kruk ME, Hermosilla S, Larson E, Mbaruku GM. Bypassing primary care clinics for childbirth: a cross-sectional study in the Pwani region, United Republic of Tanzania. Bull World Health Organ. 2014 Apr 1;92(4):246–53.

23. Alqossayir FM, Alkhowailed MS, Alammar AY, Alsaeed AA, Alamri YY, Rasheed Z. Factors associated with patients bypassing primary healthcare centres in Qassim Region, KSA. J Taibah Univ Med Sci. 2021 Dec;16(6):900–5.

24. Audo MO, Ferguson A, Njoroge PK. Quality of health care and its effects in the utilisation of maternal and child health services in Kenya. East Afr Med J. 2005 Nov;82(11):547–53.

25. Colvin CJ, Smith HJ, Swartz A, Ahs JW, de Heer J, Opiyo N, et al. Understanding careseeking for child illness in sub-Saharan Africa: a systematic review and conceptual framework based on qualitative research of household recognition and response to child diarrhoea, pneumonia and malaria. Soc Sci Med. 2013 Jun;86:66–78.

26. Bell SK, Delbanco T, Elmore JG, Fitzgerald PS, Fossa A, Harcourt K, et al. Frequency and Types of Patient-Reported Errors in Electronic Health Record Ambulatory Care Notes. JAMA Netw Open. 2020 Jun 1;3(6):e205867.

27. Geldsetzer P, Williams TC, Kirolos A, Mitchell S, Ratcliffe LA, Kohli-Lynch MK, et al. The Recognition of and Care Seeking Behaviour for Childhood Illness in Developing Countries: A Systematic Review. PLOS ONE. 2014 Apr 9;9(4):e93427.

28. Bruce N, Pope D, Arana B, Shiels C, Romero C, Klein R, et al. Determinants of Care Seeking for Children With Pneumonia and Diarrhea in Guatemala: Implications for Intervention Strategies. Am J Public Health. 2014 Apr;104(4):647–57.

For peer review only - http://bmjopen.bmj.com/site/about/guidelines.xhtml

29. Gage AD, Leslie HH, Bitton A, Jerome JG, Joseph JP, Thermidor R, et al. Does quality influence utilization of primary health care? Evidence from Haiti. Globalization and Health. 2018 Jun 20;14(1):59.

30. Ghosh A, Laxminarayan R. Demand- and supply-side determinants of diphtheria-pertussis-tetanus nonvaccination and dropout in rural India. Vaccine. 2017 Feb 15;35(7):1087–93.

31. Watson SI, Wroe EB, Dunbar EL, Mukherjee J, Squire SB, Nazimera L, et al. The impact of user fees on health services utilization and infectious disease diagnoses in Neno District, Malawi: a longitudinal, quasi-experimental study. BMC Health Services Research. 2016 Oct 20;16(1):595.

32. Nguyen HT, Zombré D, Ridde V, De Allegri M. The impact of reducing and eliminating user fees on facility-based delivery: a controlled interrupted time series in Burkina Faso. Health Policy and Planning. 2018 Oct 1;33(8):948–56.

33. Lagarde M, Barroy H, Palmer N. Assessing the effects of removing user fees in Zambia and Niger. J Health Serv Res Policy. 2012 Jan;17(1):30–6.

For peer review only

Supplementary Methods

Supplementary Table 1: Definition of capacity, quality and output indicators utilized in the analysis

Domain	Subdomain	Indicator	Definition	Data Source
**Capacity**	Facility Infrastructure	SRI for basic amenities and infection prevention	Service readiness index for basic amenities and infection prevention	SPA
	Recent Supervision	Supervision within the past 6 months	Proportion of providers within a facility with supervision in past 6 months	SPA
	Supervisor Feedback	Supervisor provided verbal or written feedback	Proportion of providers within a facility provided verbal or written supervision feedback	SPA
	Outreach Services	Facility provides outreach services	Facility provides sick child, growth monitoring or immunization outreach service	SPA
	Not charging PHC Fees	Facility does not charge PHC fees	Facility does not charge fees for PHC services (fees for a specific PHC service or a fixed fee that covers all services that a client receives)	SPA
**Quality**	Waiting Time < 60 minutes	Wait time less than one hour	Proportion of observed visits with wait times less than one hour	SPA
	Provider Availability	Proportion of visits over 10 minutes	The proportion of ANC, FP, LD and sick child visits that last for more than 10 minutes.	SPA
	Provider Competence	Antenatal care quality score based on WHO guidelines	Average quality score for observed ANC visits based on WHO ANC guidelines	SPA
		Family planning quality score based on WHO guidelines	Average quality score for observed family visits based on WHO family planning guidelines	
		Sick child quality score based on IMCI guidelines	Average quality score for observed sick child visits based on WHO Integrated Management of Childhood Illness (IMCI) guidelines	
	Person Centeredness	Proportion of caregivers who were told the sick child diagnosis	Proportion of observed sick child visits where the health worker told the child's caretaker what illness(es) the child has	SPA
	Safety	Proportion of rooms with all infection control items	Proportion of rooms (FP, sick child, ANC and NCD) where all infection control tracer items are present	SPA
		Adequate waste disposal	Average score (out of 3) on adherence to standards for disposing of medical and hazardous waste, sharps, and having guidelines for waste disposal in place.	
	Comprehensiveness	Proportion of 3 NCD services offering diagnosis and treatment (diabetes, CRD, CVD)	Proportion of non-combinable disease services provided and for which guidelines are available (diabetes, chronic respiratory disease, and chronic cardiovascular disease) across all facilities	SPA
		Proportion of 3 primary ID services provided (STI, TB, HIV)	Proportion of infectious diseases services provided and for which guidelines are available for sexually transmitted infections, tuberculosis, and HIV across all facilities.	SPA
		Average availability of 5 tracer RMNCH services (SC, Vacc, FP, ANC, PMTCT)	Proportion of maternal and child health services provided and for which guidelines are available (sick child, vaccination, family planning, antenatal care, and prevention of mother-to-child transmission of HIV) across all facilities.	SPA
**Outputs**	Maternal	Antenatal care coverage - at least four visits (%)	Antenatal care coverage (4+) visits is the percent of women with a live birth who received antenatal care (ANC) 4 or more times.	DHS
		Demand for family planning satisfied with modern methods (%)	Proportion of married or in-union women of reproductive age (aged 15-49 years) who are married or in a union and have their need for family planning satisfied with modern methods.	DHS
		Perceived barriers due to treatment costs	Percentage of women who report specific problems in accessing health care when they are sick due to issues related to getting money for treatment	DHS
		Perceived barriers due to distance	Percentage of women who report specific problems in accessing health care when they are sick due to distance that they have to travel for treatment	DHS
	Child	Coverage of DTP3 immunization	Diphtheria-tetanus-pertussis (DTP) coverage measures the percent of one-year-olds who have received three doses of the combined diphtheria, tetanus toxoid and pertussis vaccine in a given year.	DHS
		DPT Dropout rate	Percent of children who do not receive three doses of DTP after receiving an initial dose. Calculated as (DPT1-DPT3/DPT1)	DHS
		Children aged < 5 years with pneumonia symptoms taken to a healthcare provider (%)	Percentage of children under 5 years of age with suspected pneumonia (cough and difficulty breathing NOT due to a problem in the chest and a blocked nose) in the two weeks preceding the survey taken to an appropriate health facility or provider.	DHS
		Children aged < 5 years with diarrhea receiving oral rehydration salts	The percent of children with diarrhea, a leading cause of death in children under five, who received appropriate treatment with oral rehydration solution.	DHS

Supplementary Figure 1: Linkage of SPA and DHS datasets

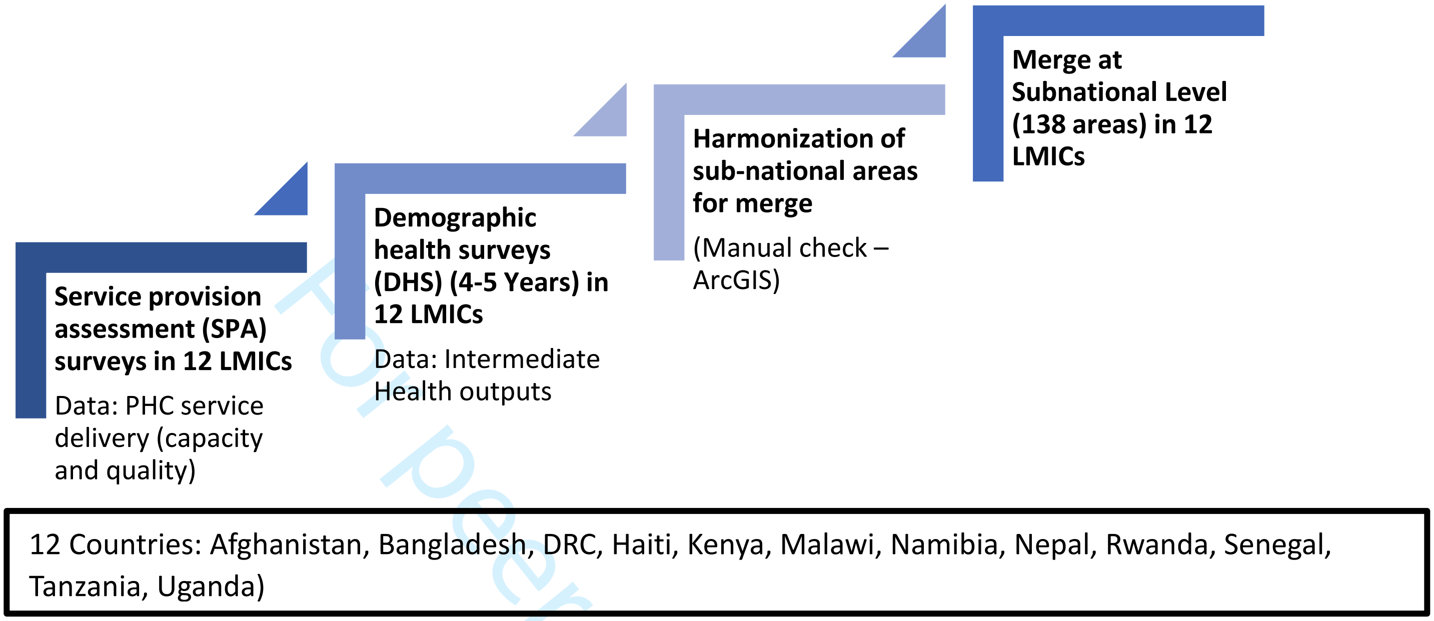

For peer review only

Supplementary Results

Supplementary Table 2: Capacity of PHC service delivery at the subnational level in the studied countries

Country	Division	Facility Infrastructure	Recent Supervision	Supervisor Feedback	Outreach Services	Not Charging PHC Fees	Average Capacity score	Range across capacity sub-domains*
Afghanistan	Balkh	83%	85%	63%	7%	56%	59%	78%
	Herat	85%	59%	29%	4%	71%	50%	81%
	Kabul	86%	63%	54%	31%	36%	54%	55%
	Kandahar	82%	99%	73%	29%	45%	66%	70%
	Kunduz	76%	91%	76%	0%	53%	59%	91%
	Nangarhar	75%	70%	63%	0%	45%	51%	75%
	Paktya	74%	70%	44%	25%	43%	51%	49%
Bangladesh	Barisal	49%	95%	93%	14%	67%	64%	81%
	Chittagong	51%	91%	85%	20%	80%	65%	71%
	Dhaka	52%	92%	89%	26%	54%	63%	66%
	Khulna	54%	97%	92%	21%	54%	64%	76%
	Mymensingh	46%	99%	96%	14%	45%	60%	85%
	Rajshahi	55%	96%	94%	19%	81%	69%	77%
	Rangpur	51%	97%	96%	14%	76%	67%	83%
	Sylhet	48%	89%	87%	18%	78%	64%	71%
DRC	Bas-uele	37%	68%	66%	91%	73%	67%	54%
	Equateur	42%	80%	71%	95%	24%	62%	71%
	Haut-katanga	45%	73%	63%	73%	46%	60%	28%
	Haut-lomami	53%	57%	49%	29%	56%	49%	28%
	Haut-uele	42%	75%	68%	81%	44%	62%	39%
	Ituri	51%	79%	74%	82%	30%	63%	52%
	Kasai	43%	72%	63%	25%	34%	47%	47%
	Kasai-central	49%	86%	86%	84%	40%	69%	46%
	Kasai-oriental	43%	83%	79%	74%	59%	67%	40%
	Kinshasa	60%	64%	56%	33%	66%	56%	33%

	Kongo central	55%	58%	54%	40%	58%	53%	18%
	Kwango	48%	91%	86%	95%	16%	67%	79%
	Kwilu	48%	71%	64%	90%	20%	58%	70%
	Lomami	47%	91%	91%	86%	37%	71%	54%
	Lualaba	54%	77%	76%	54%	65%	65%	23%
	Mai-ndombe	47%	85%	77%	92%	36%	67%	56%
	Maniema	57%	89%	85%	65%	65%	72%	32%
	Mongala	40%	87%	65%	78%	39%	62%	48%
	Nord-kivu	43%	91%	84%	94%	0%	63%	94%
	Nord-ubangi	55%	83%	82%	64%	34%	64%	49%
	Sankuru	36%	64%	56%	83%	81%	64%	47%
	Sud-kivu	49%	88%	85%	86%	0%	62%	88%
	Sud-ubangi	60%	75%	71%	69%	16%	58%	59%
	Tanganyka	47%	72%	71%	75%	64%	66%	28%
	Tshopo	41%	71%	67%	67%	52%	60%	30%
	Tshuapa	44%	84%	81%	94%	36%	68%	58%
**Haiti**	Artibonite	60%	78%	70%	67%	62%	67%	18%
	Centre	58%	90%	79%	70%	72%	74%	32%
	Grand'anse	66%	84%	76%	83%	78%	77%	18%
	Nippes	67%	64%	52%	29%	58%	54%	38%
	Nord	68%	73%	56%	42%	68%	61%	31%
	Nord-est	61%	66%	60%	45%	64%	59%	21%
	Nord-ouest	63%	58%	56%	4%	84%	53%	80%
	Ouest	59%	72%	63%	13%	76%	57%	63%
	Sud	56%	75%	49%	20%	98%	60%	78%
	Sud-est	63%	83%	75%	22%	68%	62%	61%
**Kenya**	Central	69%	76%	66%	64%	46%	64%	30%
	Coast	69%	71%	65%	53%	70%	66%	18%
	Eastern	71%	70%	60%	46%	64%	62%	25%
	Nairobi	61%	81%	72%	71%	72%	71%	20%
	North eastern	61%	83%	70%	42%	63%	64%	41%
	Nyanza	62%	69%	52%	31%	92%	61%	61%
	Rift valley	72%	67%	60%	14%	66%	56%	58%
	Western	61%	73%	62%	20%	63%	56%	53%

**Malawi**	Central region	68%	73%	64%	67%	67%	68%	9%
	Northern region	65%	75%	67%	78%	82%	73%	17%
	Southern region	69%	74%	65%	64%	72%	69%	10%
**Namibia**	Caprivi	58%	77%	49%	3%	97%	57%	94%
	Erongo	73%	63%	58%	18%	95%	61%	77%
	Hardap	78%	78%	69%	17%	83%	65%	66%
	Karas	72%	73%	68%	15%	92%	64%	77%
	Kavango	61%	71%	53%	21%	97%	60%	76%
	Khomas	74%	56%	50%	0%	75%	51%	75%
	Kunene	63%	69%	56%	14%	93%	59%	79%
	Ohangwena	66%	83%	68%	21%	100%	68%	79%
	Omaheke	75%	84%	82%	25%	100%	73%	75%
	Omusati	72%	79%	68%	42%	100%	72%	58%
	Oshana	68%	78%	66%	43%	100%	71%	57%
	Oshikoto	70%	70%	57%	18%	95%	62%	77%
	Otjozondjupa	74%	52%	47%	20%	97%	58%	77%
**Nepal**	Central	54%	71%	61%	73%	92%	70%	38%
	Eastern	54%	66%	60%	86%	93%	72%	39%
	Far-western	52%	76%	67%	88%	90%	75%	38%
	Mid-western	53%	71%	64%	88%	90%	73%	37%
	Western	54%	74%	69%	87%	94%	76%	40%
**Rowanda**	East	56%	91%	79%	81%	97%	81%	41%
	Kigali city	70%	56%	48%	69%	92%	67%	44%
	North	60%	84%	75%	80%	100%	80%	40%
	South	64%	91%	86%	87%	93%	84%	29%
	West	62%	90%	83%	77%	98%	82%	36%
**Senegal**	Dakar	79%	64%	62%	22%	67%	59%	57%
	Diourbel	83%	81%	80%	64%	60%	74%	23%
	Fatick	66%	91%	89%	37%	57%	68%	54%
	Kaffrine	47%	86%	86%	45%	72%	67%	41%
	Kaolack	51%	68%	67%	16%	66%	54%	52%
	Kedougou	45%	78%	78%	58%	66%	65%	33%
	Kolda	45%	86%	81%	61%	69%	69%	41%

	Louga	61%	58%	57%	53%	65%	59%	12%
	Matam	63%	73%	70%	58%	66%	66%	15%
	Saint-louis	62%	74%	74%	56%	60%	65%	18%
	Sedhiou	68%	100%	98%	51%	61%	75%	49%
	Tambacounda	61%	69%	66%	77%	66%	68%	16%
	This	61%	69%	68%	46%	67%	62%	23%
	Ziguinchor	70%	87%	86%	35%	64%	68%	52%
**Tanzania**	Arusha	62%	77%	68%	19%	14%	48%	63%
	Dar es salaam	49%	68%	67%	47%	11%	48%	57%
	Dodoma	73%	59%	55%	15%	52%	51%	58%
	Geita	54%	57%	54%	57%	17%	48%	40%
	Iringa	57%	58%	57%	58%	23%	51%	35%
	Kagera	55%	47%	41%	63%	15%	44%	48%
	Kaskazini pemba	59%	85%	78%	76%	29%	65%	56%
	Kaskazini unguja	62%	78%	74%	50%	86%	70%	36%
	Katavi	66%	83%	81%	40%	84%	71%	44%
	Kigoma	49%	80%	70%	82%	14%	59%	68%
	Kilimanjaro	52%	79%	66%	26%	25%	50%	54%
	Kusini pemba	61%	75%	66%	34%	41%	55%	41%
	Kusini unguja	61%	68%	64%	59%	86%	68%	27%
	Lindi	64%	78%	77%	15%	81%	63%	66%
	Manyara	52%	86%	77%	38%	26%	56%	60%
	Mara	53%	86%	78%	37%	35%	58%	51%
	Mbeya	54%	74%	70%	47%	22%	53%	52%
	Mjini magharibi	55%	59%	56%	59%	22%	50%	37%
	Morogoro	57%	65%	62%	56%	25%	53%	40%
	Mtwara	66%	45%	43%	16%	48%	44%	50%
	Mwanza	60%	48%	40%	73%	21%	49%	52%
	Njombe	51%	66%	56%	40%	13%	45%	53%
	Pwani	53%	54%	53%	51%	23%	47%	31%
	Rukwa	49%	44%	40%	54%	4%	38%	50%
	Ruvuma	51%	45%	36%	67%	28%	45%	39%
	Shinyanga	49%	95%	92%	70%	31%	67%	64%

	Simiyu	53%	79%	72%	64%	12%	56%	67%
	Singida	54%	90%	89%	75%	32%	68%	58%
	Tabora	51%	75%	74%	66%	15%	56%	60%
	Tanga	50%	90%	90%	38%	18%	57%	72%
**Uganda**	Central 1	52%	91%	78%	83%	100%	81%	48%
	Central 2	44%	91%	70%	77%	97%	76%	53%
	East central	55%	82%	69%	85%	100%	78%	45%
	Eastern	70%	60%	49%	57%	93%	66%	44%
	Kampala	54%	85%	68%	85%	100%	78%	46%
	North	45%	79%	34%	84%	100%	68%	66%
	Southwest	55%	82%	59%	82%	100%	76%	45%
	West-nile	55%	69%	57%	67%	100%	70%	45%
	Western	64%	93%	72%	94%	100%	85%	36%

For peer review only

Supplementary Table 3: Quality of PHC service delivery at the subnational level in the studied countries

Country	Division	Waiting Time	Provider Availability	Provider Competence	Person Centeredness	Safety	Comprehensive-ness	Average Quality score	Range across quality Subdomains *
Afghanistan	Balkh	89%	35%	28%	75%	28%	65%	53%	61%
	Herat	84%	34%	34%	50%	33%	52%	48%	51%
	Kabul	91%	20%	24%	34%	52%	67%	48%	71%
	Kandahar	100%	34%	19%	1%	55%	75%	47%	99%
	Kunduz	87%	42%	38%	89%	6%	69%	55%	83%
	Nangarhar	97%	48%	34%	45%	17%	78%	53%	80%
	Paktya	94%	54%	30%	22%	23%	73%	49%	72%
Bangladesh	Barisal	-	-	-	-	39%	67%	53%	28%
	Chittagong	-	-	-	-	39%	64%	52%	25%
	Dhaka	-	-	-	-	40%	61%	50%	21%
	Khulna	-	-	-	-	39%	64%	52%	25%
	Mymensingh	-	-	-	-	31%	65%	48%	34%
	Rajshahi	-	-	-	-	44%	68%	56%	24%
	Rangpur	-	-	-	-	45%	50%	47%	5%
	Sylhet	-	-	-	-	33%	68%	51%	35%
DRC	Bas-uele	74%	68%	39%	45%	35%	70%	55%	39%
	Equateur	90%	86%	36%	46%	28%	74%	60%	62%
	Haut-katanga	82%	89%	45%	40%	22%	86%	61%	67%
	Haut-lomami	78%	65%	43%	22%	45%	87%	57%	65%
	Haut-uele	63%	64%	34%	20%	26%	70%	46%	50%
	Ituri	89%	75%	39%	24%	39%	56%	54%	65%
	Kasai	95%	82%	30%	8%	19%	53%	48%	87%
	Kasai-central	73%	78%	39%	45%	30%	68%	56%	48%

	Kasai-oriental	69%	72%	38%	39%	28%	79%	54%	51%
	Kinshasa	44%	69%	48%	22%	29%	79%	48%	57%
	Kongo central	83%	88%	44%	33%	34%	88%	62%	55%
	Kwango	87%	87%	40%	11%	28%	71%	54%	76%
	Kwilu	65%	67%	37%	12%	37%	87%	51%	75%
	Lomami	70%	66%	37%	28%	36%	91%	55%	63%
	Lualaba	65%	63%	47%	40%	34%	91%	56%	57%
	Mai-ndombe	75%	86%	38%	23%	27%	78%	55%	63%
	Maniema	78%	71%	37%	46%	42%	94%	61%	57%
	Mongala	85%	89%	36%	50%	21%	71%	59%	68%
	Nord-kivu	65%	78%	42%	47%	30%	58%	54%	48%
	Nord-ubangi	77%	76%	48%	9%	49%	79%	56%	70%
	Sankuru	88%	87%	35%	30%	27%	49%	53%	61%
	Sud-kivu	75%	65%	40%	33%	36%	81%	55%	48%
	Sud-ubangi	71%	82%	44%	49%	43%	68%	60%	39%
	Tanganyka	80%	87%	33%	31%	42%	52%	54%	56%
	Tshopo	81%	88%	37%	20%	27%	71%	54%	68%
	Tshuapa	82%	88%	38%	41%	30%	69%	58%	58%
**Haiti**	Artibonite	50%	62%	36%	15%	51%	79%	49%	64%
	Centre	66%	70%	33%	7%	56%	88%	53%	81%
	Grand'anse	48%	71%	37%	8%	61%	85%	51%	77%
	Nippes	42%	59%	34%	18%	45%	75%	45%	57%
	Nord	52%	67%	36%	13%	55%	83%	51%	70%
	Nord-est	46%	68%	39%	10%	53%	77%	49%	67%
	Nord-ouest	64%	67%	49%	34%	76%	70%	60%	42%
	Ouest	70%	57%	44%	48%	51%	78%	58%	34%
	Sud	93%	54%	37%	44%	45%	64%	56%	56%
	Sud-est	53%	72%	58%	58%	60%	89%	65%	36%

Kenya	Central	30%	60%	36%	9%	55%	81%	45%	72%
	Coast	49%	63%	35%	14%	70%	90%	54%	76%
	Eastern	54%	63%	35%	15%	58%	83%	51%	68%
	Nairobi	53%	72%	32%	20%	56%	83%	52%	63%
	North eastern	73%	59%	44%	39%	66%	89%	62%	50%
	Nyanza	84%	34%	46%	71%	78%	72%	64%	50%
	Rift valley	65%	77%	50%	70%	71%	71%	67%	27%
	Western	75%	75%	51%	68%	67%	76%	69%	25%
Malawi	Central region	49%	43%	38%	51%	50%	75%	51%	37%
	Northern region	46%	42%	37%	41%	46%	77%	48%	40%
	Southern region	49%	39%	37%	43%	51%	72%	48%	35%
Namibia	Caprivi	30%	70%	52%	29%	81%	93%	59%	64%
	Erongo	59%	49%	52%	76%	65%	77%	63%	28%
	Hardap	49%	69%	51%	45%	86%	84%	64%	41%
	Karas	65%	59%	48%	45%	78%	87%	64%	42%
	Kavango	34%	48%	49%	30%	75%	94%	55%	64%
	Khomas	49%	69%	51%	84%	86%	55%	66%	37%
	Kunene	73%	76%	59%	79%	78%	86%	75%	27%
	Ohangwena	8%	55%	59%	63%	70%	93%	58%	85%
	Omaheke	58%	79%	67%	79%	91%	90%	77%	33%
	Omusati	39%	59%	49%	35%	69%	92%	57%	57%
	Oshana	25%	62%	60%	65%	59%	84%	59%	59%
	Oshikoto	34%	74%	64%	53%	54%	95%	62%	61%
	Otjozondjupa	46%	48%	47%	26%	61%	79%	51%	53%
Nepal	Central	96%	31%	26%	27%	42%	76%	50%	70%
	Eastern	90%	47%	31%	40%	44%	76%	55%	59%
	Far-western	94%	58%	34%	37%	42%	79%	57%	60%

	Mid-western	91%	45%	32%	27%	49%	74%	53%	64%
	Western	96%	41%	33%	37%	49%	75%	55%	63%
**Rowanda**	East	31%	41%	40%	13%	21%	79%	37%	66%
	Kigali city	52%	51%	45%	51%	31%	57%	48%	26%
	North	17%	34%	41%	27%	24%	76%	37%	59%
	South	43%	33%	35%	19%	32%	83%	41%	64%
	West	42%	37%	44%	18%	24%	78%	40%	60%
**Senegal**	Dakar	71%	63%	42%	24%	67%	70%	56%	47%
	Diourbel	99%	71%	42%	10%	81%	77%	63%	89%
	Fatick	94%	70%	42%	10%	72%	58%	58%	84%
	Kaffrine	71%	61%	47%	4%	53%	52%	48%	67%
	Kaolack	78%	69%	48%	29%	58%	53%	56%	49%
	Kedougou	86%	77%	49%	8%	45%	56%	53%	78%
	Kolda	76%	71%	44%	6%	49%	51%	50%	70%
	Louga	63%	49%	34%	1%	59%	74%	47%	73%
	Matam	72%	24%	28%	1%	75%	74%	46%	74%
	Saint-louis	43%	49%	33%	2%	68%	73%	45%	71%
	Sedhiou	91%	72%	38%	1%	79%	64%	58%	90%
	Tambacounda	62%	59%	32%	0%	69%	81%	50%	81%
	This	74%	60%	43%	20%	61%	64%	54%	54%
	Ziguinchor	97%	74%	37%	12%	74%	66%	60%	85%
**Tanzania**	Arusha	74%	76%	45%	44%	63%	59%	60%	32%
	Dar es salaam	59%	82%	42%	68%	35%	72%	60%	47%
	Dodoma	76%	76%	40%	44%	52%	56%	57%	36%
	Geita	71%	87%	35%	13%	26%	71%	51%	74%
	Iringa	42%	60%	44%	78%	30%	75%	54%	48%
	Kagera	76%	70%	45%	52%	37%	74%	59%	39%

	Kaskazini pemba	28%	72%	46%	77%	35%	76%	56%	49%
	Kaskazini unguja	71%	74%	39%	19%	27%	76%	51%	57%
	Katavi	22%	81%	47%	65%	66%	87%	61%	65%
	Kigoma	46%	74%	35%	67%	31%	83%	56%	52%
	Kilimanjaro	28%	56%	40%	41%	24%	83%	45%	59%
	Kusini pemba	84%	82%	35%	33%	54%	68%	59%	51%
	Kusini unguja	67%	82%	38%	23%	25%	74%	51%	59%
	Lindi	58%	77%	44%	67%	58%	89%	65%	45%
	Manyara	66%	61%	34%	57%	42%	63%	54%	32%
	Mara	54%	79%	48%	27%	47%	57%	52%	52%
	Mbeya	69%	74%	33%	24%	36%	61%	49%	50%
	Mjini magharibi	68%	59%	35%	46%	50%	78%	56%	43%
	Morogoro	61%	50%	36%	16%	61%	70%	49%	54%
	Mtwara	72%	57%	33%	50%	41%	66%	53%	39%
	Mwanza	41%	68%	38%	54%	44%	71%	53%	33%
	Njombe	55%	65%	43%	78%	36%	68%	58%	42%
	Pwani	41%	71%	47%	44%	36%	70%	52%	35%
	Rukwa	81%	76%	45%	27%	40%	60%	55%	54%
	Ruvuma	62%	43%	29%	19%	36%	75%	44%	56%
	Shinyanga	50%	76%	36%	48%	39%	65%	52%	40%
	Simiyu	64%	69%	37%	38%	34%	58%	50%	35%
	Singida	50%	74%	48%	81%	34%	70%	60%	47%
	Tabora	39%	64%	39%	30%	44%	63%	46%	34%
	Tanga	50%	70%	52%	49%	34%	80%	56%	46%
**Uganda**	Central 1	64%	46%	44%	20%	27%	81%	47%	61%
	Central 2	69%	31%	54%	64%	23%	65%	51%	46%

	East central	53%	46%	56%	71%	27%	70%	54%	44%
	Eastern	50%	51%	56%	72%	37%	86%	59%	49%
	Kampala	47%	36%	60%	82%	23%	76%	54%	59%
	North	22%	21%	39%	36%	17%	61%	33%	44%
	Southwest	33%	51%	40%	39%	36%	72%	45%	39%
	West-nile	66%	49%	48%	65%	22%	64%	52%	44%
	Western	34%	42%	43%	39%	24%	73%	43%	49%

For peer review only

For peer review only

The RECORD statement – checklist of items, extended from the STROBE statement, that should be reported in observational studies using routinely collected health data.

	Item No.	STROBE items	Location in manuscript where items are reported	RECORD items	Location in manuscript where items are reported
**Title and abstract**					
	1	(a) Indicate the study's design with a commonly used term in the title or the abstract (b) Provide in the abstract an informative and balanced summary of what was done and what was found	(a) line 24, page 2<br><br>(b) lines 21-45, page 2	RECORD 1.1: The type of data used should be specified in the title or abstract. When possible, the name of the databases used should be included.<br><br>RECORD 1.2: If applicable, the geographic region and timeframe within which the study took place should be reported in the title or abstract.<br><br>RECORD 1.3: If linkage between databases was conducted for the study, this should be clearly stated in the title or abstract.	1.1: lines 24-26, page 2<br><br>1.2: lines 26-28, page 2<br><br>1.3: lines 24-25, page 2
**Introduction**					
Background rationale	2	Explain the scientific background and rationale for the investigation being reported	Lines 63-89, page 4		
Objectives	3	State specific objectives, including any prespecified hypotheses	Lines 90-94, page 4		
**Methods**					
Study Design	4	Present key elements of study design early in the paper	Lines 97-119, page 4		
Setting	5	Describe the setting, locations, and relevant dates, including periods of recruitment, exposure, follow-up, and data collection	Lines 99-119, page 4-5<br>Table 1-page 5		

For peer review only

Participants	6	*(a) Cohort study* - Give the eligibility criteria, and the sources and methods of selection of participants. Describe methods of follow-up<br>*Case-control study* - Give the eligibility criteria, and the sources and methods of case ascertainment and control selection. Give the rationale for the choice of cases and controls<br>*Cross-sectional study* - Give the eligibility criteria, and the sources and methods of selection of participants<br><br>*(b) Cohort study* - For matched studies, give matching criteria and number of exposed and unexposed<br>*Case-control study* - For matched studies, give matching criteria and the number of controls per case	Lines 99-119, page 4-5<br>Table 1, page 5	RECORD 6.1: The methods of study population selection (such as codes or algorithms used to identify subjects) should be listed in detail. If this is not possible, an explanation should be provided.<br><br>RECORD 6.2: Any validation studies of the codes or algorithms used to select the population should be referenced. If validation was conducted for this study and not published elsewhere, detailed methods and results should be provided.<br><br>RECORD 6.3: If the study involved linkage of databases, consider use of a flow diagram or other graphical display to demonstrate the data linkage process, including the number of individuals with linked data at each stage.	6.1: NA- secondary analysis of publicly available anonymized data was used<br>6.2: NA- secondary analysis of publicly available anonymized data was used<br><br>6.3: Figure S1 Supplementary methods
Variables	7	Clearly define all outcomes, exposures, predictors, potential confounders, and effect modifiers. Give diagnostic criteria, if applicable.	Lines 125-147, Page 6<br><br>Table S1, supplementary methods	RECORD 7.1: A complete list of codes and algorithms used to classify exposures, outcomes, confounders, and effect modifiers should be provided. If these cannot be reported, an explanation should be provided.	Lines 125-145, Page 5<br><br>Table S1, supplementary methods
Data sources/ measurement	8	For each variable of interest, give sources of data and details of methods of assessment (measurement).<br>Describe comparability of assessment methods if there is more than one group	Lines 118-141, Page 5<br><br>Table S2, supplementary file 1		

For peer review only

Bias	9	Describe any efforts to address potential sources of bias	Lines 159-170, page 7		
Study size	10	Explain how the study size was arrived at	Secondary analysis of public data was conducted. The selected sample was explained<br>Lines 99-103, page 4-5<br>Table 1, page 5		
Quantitative variables	11	Explain how quantitative variables were handled in the analyses. If applicable, describe which groupings were chosen, and why	Lines 125-147, page 6		
Statistical methods	12	(a) Describe all statistical methods, including those used to control for confounding<br>(b) Describe any methods used to examine subgroups and interactions<br>(c) Explain how missing data were addressed<br>(d) *Cohort study* - If applicable, explain how loss to follow-up was addressed<br>*Case-control study* - If applicable, explain how matching of cases and controls was addressed<br>*Cross-sectional study* - If applicable, describe analytical methods taking account of sampling strategy<br>(e) Describe any sensitivity analyses	a) lines 149-170, page 7<br><br>b) NA<br><br>c)lines 153, 158 - page 7<br><br>d) NA<br><br>e) NA		

For peer review only

Data access and cleaning methods		..		RECORD 12.1: Authors should describe the extent to which the investigators had access to the database population used to create the study population.<br><br>RECORD 12.2: Authors should provide information on the data cleaning methods used in the study.	12.1: Data is publicly available so section 12.1 does not apply<br><br>12.2: NA as access to already cleaned data was provided through DHS website
Linkage		..		RECORD 12.3: State whether the study included person-level, institutional-level, or other data linkage across two or more databases. The methods of linkage and methods of linkage quality evaluation should be provided.	12.3: lines 149-158, pages 7
**Results**					
Participants	13	(a) Report the numbers of individuals at each stage of the study (*e.g.*, numbers potentially eligible, examined for eligibility, confirmed eligible, included in the study, completing follow-up, and analysed)<br>(b) Give reasons for non-participation at each stage.<br>(c) Consider use of a flow diagram	Lines 168-169, page 6	RECORD 13.1: Describe in detail the selection of the persons included in the study (*i.e.,* study population selection) including filtering based on data quality, data availability and linkage. The selection of included persons can be described in the text and/or by means of the study flow diagram.	Table 1, page 4-6 and Supplementary Figure 21 in Supplementary Methods
Descriptive data	14	(a) Give characteristics of study participants (*e.g.*, demographic, clinical, social) and information	Demographic of patients is not applicable; characteristics of		

		on exposures and potential confounders<br>(b) Indicate the number of participants with missing data for each variable of interest<br>(c) *Cohort study* - summarise follow-up time (*e.g.*, average and total amount)	included surveys are described in Table 1 -page 5		
Outcome data	15	*Cohort study* - Report numbers of outcome events or summary measures over time<br>*Case-control study* - Report numbers in each exposure category, or summary measures of exposure<br>*Cross-sectional study* - Report numbers of outcome events or summary measures	Table 2 – page 8<br>Table 3- page 9-10<br>Table 4- page 10-11		
Main results	16	(a) Give unadjusted estimates and, if applicable, confounder-adjusted estimates and their precision (e.g., 95% confidence interval). Make clear which confounders were adjusted for and why they were included<br>(b) Report category boundaries when continuous variables were categorized<br>(c) If relevant, consider translating estimates of relative risk into absolute risk for a meaningful time period	Table 3, page 9-10<br>&<br>Table 4, page 10-11 standard errors were included and confounders are added as footnotes<br><br>NA<br><br>NA		
Other analyses	17	Report other analyses done—e.g., analyses of subgroups and	NA		

For peer review only

		interactions, and sensitivity analyses			
**Discussion**					
Key results	18	Summarise key results with reference to study objectives	Lines 273-307, page 11-12		
Limitations	19	Discuss limitations of the study, taking into account sources of potential bias or imprecision. Discuss both direction and magnitude of any potential bias	Lines 323-337, page 12	RECORD 19.1: Discuss the implications of using data that were not created or collected to answer the specific research question(s). Include discussion of misclassification bias, unmeasured confounding, missing data, and changing eligibility over time, as they pertain to the study being reported.	Lines 323-337, page 12
Interpretation	20	Give a cautious overall interpretation of results considering objectives, limitations, multiplicity of analyses, results from similar studies, and other relevant evidence	Lines 273-322, page 11-12		
Generalisability	21	Discuss the generalisability (external validity) of the study results	NA – this is a multi-country study, all countries with available SPA survey were considered		
**Other Information**					
Funding	22	Give the source of funding and the role of the funders for the present study and, if applicable, for the original study on which the present article is based	Lines 364-366, page 13		
Accessibility of protocol, raw data, and programming code		..		RECORD 22.1: Authors should provide information on how to access any supplemental information such as the study protocol, raw data, or programming code.	Lines 358-360, page 13

For peer review only

*Reference: Benchimol EI, Smeeth L, Guttmann A, Harron K, Moher D, Petersen I, Sørensen HT, von Elm E, Langan SM, the RECORD Working Committee. The REporting of studies Conducted using Observational Routinely-collected health Data (RECORD) Statement. *PLoS Medicine* 2015; in press.

*Checklist is protected under Creative Commons Attribution (CC BY) license.

For peer review only

BMJ Open

Capacity and quality of maternal and child health services delivery at the subnational primary health care level in relation to intermediate health outputs: a cross-sectional study of 12 low-income and middle-income countries

Journal:	*BMJ Open*
Manuscript ID	bmjopen-2022-065223.R3
Article Type:	Original research
Date Submitted by the Author:	09-Jan-2023

Complete List of Authors:	Ramadan, Marwa; World Bank Group<br>Gutierrez, Jose Carlos; World Bank Group<br>Feil, Cameron; World Bank Group<br>Bolongaita, Sarah; World Bank Group<br>Bernal, Oscar; World Bank Group<br>Villar-Uribe , Manuela; World Bank Group

**Primary Subject Heading**:	Global health
Secondary Subject Heading:	Global health, Health policy, Public health
Keywords:	PRIMARY CARE, Quality in health care < HEALTH SERVICES ADMINISTRATION & MANAGEMENT, PUBLIC HEALTH, International health services < HEALTH SERVICES ADMINISTRATION & MANAGEMENT

SCHOLARONE™
Manuscripts

I, the Submitting Author has the right to grant and does grant on behalf of all authors of the Work (as defined in the below author licence), an exclusive licence and/or a non-exclusive licence for contributions from authors who are: i) UK Crown employees; ii) where BMJ has agreed a CC-BY licence shall apply, and/or iii) in accordance with the terms applicable for US Federal Government officers or employees acting as part of their official duties; on a worldwide, perpetual, irrevocable, royalty-free basis to BMJ Publishing Group Ltd ("BMJ") its licensees and where the relevant Journal is co-owned by BMJ to the co-owners of the Journal, to publish the Work in this journal and any other BMJ products and to exploit all rights, as set out in our licence.

The Submitting Author accepts and understands that any supply made under these terms is made by BMJ to the Submitting Author unless you are acting as an employee on behalf of your employer or a postgraduate student of an affiliated institution which is paying any applicable article publishing charge ("APC") for Open Access articles. Where the Submitting Author wishes to make the Work available on an Open Access basis (and intends to pay the relevant APC), the terms of reuse of such Open Access shall be governed by a Creative Commons licence – details of these licences and which Creative Commons licence will apply to this Work are set out in our licence referred to above.

Other than as permitted in any relevant BMJ Author's Self Archiving Policies, I confirm this Work has not been accepted for publication elsewhere, is not being considered for publication elsewhere and does not duplicate material already published. I confirm all authors consent to publication of this Work and authorise the granting of this licence.

For peer review only

Capacity and quality of maternal and child health services delivery at the subnational primary health care level in relation to intermediate health outputs: a cross-sectional study of 12 low-income and middle-income countries

Marwa Ramadan[1]*, Jose Carlos Gutierrez[1], Cameron Feil[1], Sarah Bolongaita[1], Oscar Bernal[1], Manuela Villar Uribe[1]

[1]Health Nutrition and Population Global Practice, World Bank Group, Washington, DC, USA.

***Correspondence to:**

Marwa Ramadan

Health Nutrition and Population Global Practice, World Bank Group, Washington, DC, USA

mramadan@worldbank.org

Keywords

Primary healthcare; healthcare quality; measurement; performance assessment; subnational; low- and middle-income countries

For peer review only

For peer review only - http://bmjopen.bmj.com/site/about/guidelines.xhtml

Abstract

Objectives: To examine the capacity and quality of Maternal and Child Health (MCH) services at the subnational primary health care (PHC) level in 12 Low-and Middle-Income Countries (LMICs) and its association with intermediate health outputs such as coverage and access to care.

Design: Observational cross-sectional study using matched subnational data from service provision assessment surveys and demographic health surveys from 2007-2019.

Settings: 138 sub-national areas with available survey data in 12 LMICs (Afghanistan, Bangladesh, Democratic Republic of Congo, Haiti, Kenya, Malawi, Namibia, Nepal, Rwanda, Senegal, Tanzania, Uganda).

Outcomes: Eight intermediate MCH outcomes/outputs were explored: 1) met need for family planning by modern methods; 2) attendance of four or more antenatal care visits; 3) perceived financial barriers to care; 4) perceived geographic barriers to care; 5) Diphtheria-Pertussis- Tetanus (DPT) third dose coverage; 6) DPT dropout-rate; 7) care-seeking for pneumonia; and 8) oral rehydration solutions coverage.

Results: Overall, moderate to poor PHC performance was observed across the 12 countries, with substantial heterogeneity between the different subnational areas in the same country as well as within the same subnational area across both capacity and quality subdomains. The analysis of the relationship between PHC service delivery and child health outcomes revealed that recent supervision [$b=0.34$, $p<0.01$] and supervisors' feedback [$b=0.28$, $p<0.05$] were each associated with increased care-seeking for pneumonia. We also observed the associations of several measures of capacity and quality with DPT immunization. The analysis of maternal health outcomes yielded only a few statistically significant results at $p<0.05$ level, however, none remained significant after adjusting for other covariates.

Conclusion: The results of this analysis illustrate the heterogeneity in the capacity and quality of PHC service delivery within LMICs. Countries seeking to strengthen their PHC systems could improve PHC monitoring at the subnational level to better understand subnational bottlenecks in service delivery.

For peer review only

For peer review only - http://bmjopen.bmj.com/site/about/guidelines.xhtml

Strengths and limitations of this study

- Rather than focusing on primary health care (PHC) delivery at the national level, this study bridges a research gap by examining PHC service delivery at the subnational level and its association with improved health outputs in 12 low-and middle-income countries.
- The methodology utilized in the present study demonstrates how publicly available survey data such as the Demographic Health Survey (DHS) and the Service Provision Assessment (SPA) survey can be valuable tools in highlighting bottlenecks and disparities in PHC service delivery at the subnational level.
- As the study is observational and cross-sectional, the association presented should not be interpreted as causal given the potential for an ecological bias.
- Although the sample was constructed such that the time between the SPA and DHS surveys in each country was less than 5 years, it is possible that changes in time-varying factors could have influenced the outcomes under study.
- There is a potential for omitted variable bias through other factors that may influence the capacity, quality, and performance variables under study but are unmeasured in the SPA and DHS surveys.

For peer review only

For peer review only - http://bmjopen.bmj.com/site/about/guidelines.xhtml

Introduction

Despite renewed global commitment to primary healthcare (PHC) in recent years, the measurement of PHC capacity and quality remains a challenge for policymakers seeking to improve the performance of health services. The transition from the Millennium Development Goals (MDGs) to Sustainable Development Goals (SDGs) in 2016 marked a significant shift in the global health agenda. SDG 3, "Achieving Universal Healthcare Coverage," was a call to action to strengthen health systems, shifting the global health agenda away from vertically planned health programs and initiatives. In 2018, forty years after the Alma-Alta Declaration which identified PHC as the pathway to achieving health for all, global actors, governments, and organizations re-affirmed a global commitment to PHC with the issuance of the Astana Declaration (1).

More significant efforts are required to improve primary health care performance in Low-and-Middle-income countries (LMICS) (2). Performance measurement has the potential to catalyze PHC improvement by identifying bottlenecks (3,4). The Primary Health Care Performance Initiative (PHCPI), a global collaborative dedicated to measuring and improving PHC systems in LMICs, developed a conceptual framework illustrating the relationships between financing, inputs, and core functions of the PHC system (2). In 2018, PHCPI operationalized the Conceptual Framework into the Vital Signs Profile, which uses qualitative and quantitative indicators to provide a comprehensive understanding of PHC performance in a county (3).

The relationship between capacity, quality and health system performance has long been studied. Donabedian captured the causal chain of inputs to outputs, commonly defined as the 'input-process-output-model', in 1966. More recent efforts by Randhawa (4) and Veillard et al.(5) have demonstrated the relationship between inputs and outcomes of PHC system. Previous studies also highlighted that PHC capacity, including the availability of providers, governance, and financing, has been found to impact PHC-related outcomes in LMICs at the national level (6–10). In addition, several studies have identified relationships between elements of high-quality PHC and performance at the national level (2,11–14). However, the majority of associations were explored at the national level and significant gaps still exist in understanding PHC service delivery at the sub-national level and its association with improved health outputs.

This paper aims to fill some of the current measurement gaps by exploring the relationship between PHC system capacity, quality, and performance at the sub-national level using quantitative indicators from the PHCPI Vital Signs Profile. Specifically, we assess PHC service delivery at both the national and subnational levels in 12 LMICs. In addition, we examine the association of PHC capacity and quality with intermediate health outputs such as coverage and access to services at the subnational level.

Methods

Data sources

In this cross-sectional analysis, Service Provision Assessment (SPA) surveys and Demographic Health Surveys (DHS) were the main sources of data on PHC service delivery and PHC intermediate population health outputs, respectively in 12 LMICS (Afghanistan, Bangladesh, DRC (democratic republic of Congo), Haiti, Kenya, Malawi, Namibia, Nepal, Rwanda, Senegal, Tanzania, Uganda). Specifically, we included all LMICS (as defined by the world bank income classification) where both a DHS and a SPA survey were conducted within a 5-year interval in the period between 2007 and 2019. SPA surveys are comprehensive facility assessments that aim to collect information on the availability of services at the

For peer review only - http://bmjopen.bmj.com/site/about/guidelines.xhtml

facility level, the readiness of facilities to provide these services, the compliance with acceptable standards of care, and whether clients and service providers are satisfied with the service delivery process (15). SPA surveys usually involve random selection of 400-700 facilities from a comprehensive sampling list of all public and private health facilities in a country or alternatively a complete census of facilities in the country. Facilities are usually categorized by facility type, managing authority (public and non-public), as well as subnational region; sampling and sample size ensure representativeness across each of these categories. Weights are applied during the analysis of these surveys to ensure that the contribution of selected facilities is proportional to their distribution in the country. On the other hand, DHS are nationally representative household surveys and a vital source of information on population, health, and nutrition indicators in more than 90 countries. The standard DHS survey is conducted every five years using a large sample size (5000-30,000 households per survey), and usually utilizes a two-stage sampling methodology based on the availability of census information (16). The DHS sampling and sample size ensure representativeness at the subnational region level. Table 1 provides additional information on the sampling structure and the timeframe for data collection in each of the studied countries. The SPA and DHS datasets utilized in this study are publicly available and can be downloaded from the DHS website (17).

Table 1: Characteristics of DHS and SPA surveys included in the analysis

Country	Sub-national areas	DHS Field Work	DHS sampling	DHS sample size (ever-married Women)	Year data collection -SPA	SPA sample Size (Facilities)
**Afghanistan**	34 provinces	June 2015 - February 2016	two-stage stratified sample	24,395	November 2018- January 2019	160 facilities**
**Bangladesh**	8 divisions	October 24, 2017- March 2018	two-stage stratified sample	20,100	July 2017-to October 2017	1,600
**DRC**	26 new provinces	August 2013 - February 2014	Three-stage stratified sample*	18,827	October 2017 - April 2018	1380
**Haiti**	10 departments	November 2016 - April 2017	two-stage stratified sample	14,371	December 2017 - May 2018	1007
**Kenya**	8 regions	May 2014 - October 2014	two-stage stratified sample	31,079	January 2010-May 2010	695
**Malawi**	3 regions	October 2015 - February 2016	two-stage stratified sample	24562	June 2013-February 2014	1060
**Namibia**	13 regions	May 2013 - September 2013	two-stage stratified sample	9176	July 2009-October 2009	446
**Nepal**	7 provinces	June 2016 - January 2017	Stratified sample (two stages in rural areas and three stages in urban areas)	12862	April 2015-November 2015	1,000

For peer review only - http://bmjopen.bmj.com/site/about/guidelines.xhtml

**Rwanda**	5 provinces	September 2010 - March 2011	two-stage stratified sample	13671	June 2007- August 2007	538
**Senegal**	14 regions	April 2017 - December 2017	two-stage stratified sample	16787	March 2017- December 2017	3764
**Tanzania**	25 regions	August 2015 - February 2016	two-stage stratified sample	13266	October 2014 – March 2015	1,200
**Uganda**	15 regions	June 2011 - December 2011	two-stage stratified sample	8674	July-October 2007	491

*Two-stage stratified sample in statutory towns and cities of established provinces, three-stage stratified sample in rest of established provinces and new provinces, **Sampling limited to urban areas of seven major provinces (Kabul, Nangarhar, Paktya, Kunduz, Balkh, Kandahar, and Herat)

Metrics

Guided by the PHCPI framework and survey data availability, the capacity of the PHC system was assessed using the following five indicators: 1) availability of basic facility Infrastructure; 2) the presence of recent supervision within the past 6 months; 3) the provision of feedback by supervisors; 4) the provision of outreach services; and 5) absence of PHC fees defined as free immunization, RMNCH, non-communicable diseases and HIV services. Similarly, whenever data was available, PHCPI's Vital Signs Profile (VSP) guided the assessment of PHC quality of care through the following six indicators: 1) waiting time of less than 60 minutes; 2) availability of providers defined as an average consultation time of more than 10 minutes; 3) competence of providers defined as the average of antenatal care, family planning and sick child quality scores based on WHO guidelines; 4) person-centeredness defined as the percent of clients who were told their diagnosis; 5) safety defined as the presence of adequate infection control practices and waste disposal at the facility; and 6) comprehensiveness of PHC services defined as the average availability of tracer items for RMNCH, infectious diseases and non-communicable diseases. Additional information on PHCPI's framework and methodology is provided in an earlier publication (5).

Additionally, eight intermediate maternal and child health outcomes/outputs were explored in accordance with the PHCPI vital signs profile and consistent reporting of DHS indicators in the studied countries. Specifically, we assessed the following maternal and child health outputs: 1) met need for family planning by modern methods; 2) attendance of four or more antenatal care visits; 3) perceived financial barriers to care; 4) perceived geographic barriers to care; 5) DPT3 coverage; 6) DPT dropout-rate; 7) care-seeking for pneumonia; and 8) Oral Rehydration Solutions (ORS) coverage. Other PHC-related outputs (e.g., non-communicable diseases, tuberculosis, and HIV) were not assessed due to the limited availability of sub-national data in the studied countries. Detailed definition of each of the capacity, quality and output indicators are provided in Supplementary Table 1 [See Supplementary Material]

For peer review only - http://bmjopen.bmj.com/site/about/guidelines.xhtml

Data analysis

Population and health facility data were linked at the first administrative level (e.g., region, province…etc.) whenever data was available and the gap between a population-based survey and a health facility survey was less than five years. In situations where substantial variation existed at the first administrative level between the two types of surveys, geographic coordinates of health facilities and DHS clusters were input into geographic information system (GIS) software (ArcGIS) for matching. Specifically, in the Democratic Republic of Congo, DHS data in 2013 were reported for 11 formerly defined provinces, while 2017 SPA data were reported for 26 newly defined provinces. ArcGIS was used to match subnational areas in both types of surveys. In addition, in Afghanistan, the SPA survey was only conducted in seven urban subnational areas, so the match process with DHS was limited to these areas [See Supplementary Figure 1 in Supplementary Material]

To compute subnational estimates, we applied the recommended SPA weights at the facility, provider, and visit levels in the 12 studied countries. We then computed national averages based on the assumption that all subnational areas were equally weighted in each of the studied countries. In addition, we examined the subnational association between the computed service delivery sub-domains and DHS intermediate maternal and child health outputs using bivariate and multivariable models with a significance level at $p<0.05$. Given the hierarchical nature of the examined population and health facility data, we utilized a two-level fixed-effects model to account for possible clustering of the observations at the country level. We also adjusted for the following population and facility characteristics at the subnational level: proportion of urban households, proportion of population in the 5th economic quintile (highest), and proportion of women with secondary or more education, proportion of private facilities (per the total number of health facilities at the subnational level), proportion of hospitals (per the total number of health facilities at the subnational level),. The statistical analyses were all performed in STATA (version 17.0), tables were created using Microsoft Excel.

Patient and public involvement

None.

Results

PHC service delivery at the national and subnational levels

In the present study, the capacity and quality of PHC service delivery were analyzed in 138 subnational areas in 12 LMICs. Substantial heterogeneity was observed across countries in the capacity subdomain. For example, the average availability of facility infrastructure (basic amenities and infection prevention) was as high as 80% in Afghanistan and as low as 48 % in DRC. Most facilities in the analyzed subnational areas reported the presence of recent supervision, with an average ranging from 94% in Bangladesh to 70% in Tanzania, while supervisor’s feedback was provided in a relatively fewer number of facilities, with the highest average reported in Bangladesh (91%) and the lowest in Afghanistan (57 %). Meanwhile, the provision of facility outreach services varied largely across the studied countries, with 84 % of facilities in Nepal providing outreach services versus only 14 % of facilities in the analyzed subnational areas in Afghanistan. Similarly, large variation in charging PHC fees was observed across the sample; 99% of facilities in the analyzed subnational areas of Uganda did not charge fees for PHC services versus only 32 % in Tanzania. Table 2 shows the average score of individual capacity sub-domains in the analyzed subnational areas across the 12 studied countries.

For peer review only - http://bmjopen.bmj.com/site/about/guidelines.xhtml

When the quality-of-service delivery was assessed, most countries had moderate to poor scores in all quality sub-domains except waiting time and comprehensiveness, where some countries performed well. As with capacity, cross-country variation in quality sub-domains was observed. For example, the average waiting time was less than 60 minutes in more than 90 % of the facilities in the analyzed subnational areas of Afghanistan and Nepal versus only 37 % of the analyzed facilities in Rwanda. However, the average consultation time was more than 10 minutes in only 38 % of the analyzed Afghan facilities. Competence of providers was one of the quality sub-domains with the lowest average scores across the studied countries ranging from 30 % on average, for the analyzed facilities in Afghanistan to 54 % in Namibia. Similarly, person centeredness scored poorly in most countries with an average score as low as 9 % for the analyzed facilities in Senegal versus 54 % in Uganda. Meanwhile, at least two thirds of the analyzed facilities offered comprehensive PHC services including RMNCH, non-communicable and infectious disease services in all the studied countries (Table 2).

Table 2: The capacity and quality of PHC service delivery in the studied countries (average scores of subnational areas)

Domain	Subdomain	Country											
		AF18*	BD17	DRC18	HT17	KE10	MW13	NM09	NP15	RW07	SN17	TZ15	UG07
Capacity	Facility Infrastructure	80%	51%	48%	62%	66%	67%	69%	53%	63%	62%	56%	55%
	Recent Supervision	77%	94%	77%	74%	74%	74%	72%	72%	82%	77%	70%	81%
	Supervisor Feedback	57%	91%	72%	64%	63%	65%	61%	64%	74%	76%	65%	62%
	Outreach Services	14%	18%	73%	39%	43%	70%	20%	84%	79%	48%	50%	79%
	Not charging PHC Fees	50%	67%	42%	73%	67%	74%	94%	92%	96%	65%	32%	99%
	**Capacity score**	55%	65%	61%	60%	61%	70%	63%	73%	79%	64%	53%	76%
Quality	Waiting Time < 60 minutes	92%	-	76%	58%	61%	48%	44%	94%	37%	77%	57%	49%
	Provider Availability	38%	-	78%	65%	63%	41%	63%	44%	39%	62%	70%	42%
	Provider Competence	30%	-	39%	40%	41%	37%	54%	31%	41%	40%	40%	49%
	Person Centeredness	45%	-	31%	25%	38%	45%	54%	33%	26%	9%	46%	54%
	Safety	31%	39%	32%	55%	65%	49%	73%	45%	26%	65%	41%	26%
	Comprehensiveness	68%	63%	74%	79%	81%	75%	85%	76%	75%	65%	71%	72%
	**Quality**	50%	51%	55%	56%	61%	49%	62%	54%	41%	53%	54%	47%

AF18: Afghanistan 2018, BD17: Bangladesh 2017, DRC18: democratic republic of Congo 2018, HT17: Haiti 2017, KE10: Kenya 2010, MW13: Malawi 2013, NM09: Namibia 2009, NP15: Nepal 2015, RW07: Rwanda 2007, SN17: Senegal 2017, TZ15: Tanzania 2015, UG07: Uganda 2007
* Estimates represent the average of urban subnational data for which SPA and DHS data were available

Subnational analysis of service delivery sub-domains revealed the presence of geographic disparities at the first administrative level in both PHC capacity and quality sub-domains. For example, large subnational variation was observed in the average availability of facility infrastructure in Senegal,

For peer review only - http://bmjopen.bmj.com/site/about/guidelines.xhtml

ranging from 83 % in Diourbel region to 45 % in Kedougou region. Similarly, considerable subnational variation was observed in the provision of outreach services in Haiti, ranging from 45 % in North-East to 5 % in North-West department. Charging of PHC fees in DRC was also another example of administrative variation in PHC capacity, where 81 % of facilities in Sankuru province provided free PHC services versus none of the facilities in North-Kivu or South-Kivu provinces. Similarly, subnational variation was observed in most of the examined quality sub-domains. For example, the range of variation across the different subnational areas in Tanzania was more than 60 % points for waiting time. In addition, the range of variation was more than 50 % for availability of providers across the different subnational areas in Senegal. Furthermore, person centered care ranged from 89 % in Kunduz province to only 1 % in Kandahar province in Afghanistan. Meanwhile, the lowest subnational variations were observed in provider competence sub-domain, where the majority of subnational areas scored below 50 % in the 12 studied countries.

Not only did we observe variations within the same country across different subnational areas, but also within the same subnational area across both quality and capacity sub-domains. For example, the range of variation across different capacity sub-domains was more than 50 % points in the majority of the analyzed sub-national areas in Afghanistan, Bangladesh, and Namibia. Most of these variations were derived by the difference in the capacity of the facilities in providing outreach services and offering free PHC services. Similarly, several subnational areas in Afghanistan, DRC and Senegal showed substantial variation across the different quality sub-domains. For example, the range of variation across quality sub-domains in Kandahar, Afghanistan was 99 %, where all facilities had a waiting time of less than 60 minutes; however only 1 % provided person centered care. Supplementary Table 2 and Supplementary Table 3 provide detailed information on the capacity and quality scores in each of the analyzed 138 subnational areas [See Supplementary Material]

The association between PHC service delivery sub-domains and intermediate health outputs

Examining the relationship between subnational PHC capacity and maternal health outputs, no statistically significant association was found between the capacity of facilities and antenatal care coverage after adjustment for subnational characteristics. Meanwhile, a statistically significant association was observed between the general availability of basic facility infrastructure and perceived economic and geographic barriers to care. Specifically, a 10 %-point increase in the average availability of basic infrastructure at the subnational level was associated with 3 % lower perceived financial barriers to care [b = -0.35, p < 0.05] and 4 % lower perceived geographic barriers to care [b = -0.39, p < 0.05]. However, the associations were not statistically significant after adjustment for subnational facility and population characteristics (Table 3). Coverage of family planning services was also negatively associated with the provision of outreach services. However, this paradoxical association was no longer significant in the multivariable specification. Meanwhile, no statistically significant association was found between any of the examined PHC quality sub-domains and maternal health outputs (Table 3).

Table 3: The association between PHC service delivery subdomains and maternal health outputs at the subnational level

Sub-domain			Antenatal		Family planning		Cost Barrier		Geographic Barrier	
			Bivariate	Multivariable	Bivariate	Multivariable	Bivariate	Multivariable	Bivariate	Multivariable
Capacity	Facility Infrastructure	β	0.23	0.11	0.29	0.16	-0.35*	-0.23	-0.39*	-0.16
		SE	0.13	0.13	0.16	0.19	0.15	0.14	0.15	0.16
	Supervision in the past 6 months	β	-0.09	0.02	-0.12	0.01	0.05	-0.02	0.00	-0.14
		SE	0.08	0.08	0.09	0.10	0.09	0.08	0.10	0.09

For peer review only - http://bmjopen.bmj.com/site/about/guidelines.xhtml

	Supervisor Feedback	β	-0.06	0.01	-0.06	0.03	-0.03	-0.09	-0.02	-0.11
		SE	0.08	0.07	0.09	0.09	0.09	0.07	0.09	0.08
	Outreach Services	β	-0.07	0.00	-0.13*	-0.09	0.04	-0.01	0.09	0.04
		SE	0.05	0.04	0.06	0.06	0.06	0.05	0.06	0.05
	Not charging PHC fees	β	0.01	0.01	-0.01	0.00	-0.02	0.00	0.02	0.06
		SE	0.05	0.04	0.06	0.06	0.06	0.05	0.06	0.05
**Quality**	Waiting time < 60 minutes	β	-0.01	0.01	-0.11	-0.10	0.09	0.05	0.11	0.07
		SE	0.06	0.05	0.08	0.07	0.07	0.06	0.08	0.06
	Provider Competence	β	0.07	0.00	0.04	0.01	-0.28	-0.10	-0.26	-0.10
		SE	0.15	0.13	0.19	0.18	0.19	0.14	0.19	0.15
	Provider Availability	β	-0.04	-0.08	-0.16	-0.15	-0.11	-0.13	0.02	0.03
		SE	0.09	0.07	0.11	0.10	0.10	0.08	0.11	0.08
	Person centeredness	β	-0.01	-0.04	0.01	-0.02	-0.06	-0.01	-0.02	0.02
		SE	0.05	0.04	0.06	0.06	0.06	0.05	0.07	0.05
	Safety	β	0.09	0.06	0.01	-0.03	0.20	0.13	0.11	0.10
		SE	0.08	0.07	0.11	0.11	0.11	0.08	0.11	0.09
	Comprehensiveness	β	0.09	0.09	-0.05	-0.02	-0.20	-0.09	-0.14	-0.05
		SE	0.10	0.08	0.11	0.11	0.11	0.08	0.12	0.09

* p<0.05

In multivariable models, results were adjusted for proportion of private facilities, hospitals, urban households, population in the 5th economic quintile and women with secondary education or more at the sub-national level

Afghanistan was excluded from the analysis for sub-national sample limitation

On the other hand, PHC service delivery sub-domains were more likely to be statistically significantly associated with child health outputs. For example, a 10 %-point increase in the availability of basic facility infrastructure was associated with an increase of 5 % in DPT3 immunization coverage [b =0.45, p<0.05] and a decrease of 3% in DPT-dropout rates [b = - 0.30, p<0.05]. Additionally, recent facility supervision [b=0.34, p<0.01] and provision of feedback by supervisors [b=0.28, p<0.05] were each associated with increased care-seeking for pneumonia after adjustment for facility and population characteristics. Surprisingly, significantly lower immunization coverage and higher dropout rates were observed in subnational areas where facilities did not charge PHC fees, even after adjustment for facility and population characteristics (Table 4). Meanwhile, of the examined quality sub-domains, only comprehensiveness showed a statistically significant association with subnational DPT3 immunization coverage and lower drop-out rates. Specifically, a 10 %-point increase in the comprehensiveness of PHC services was associated with 2 % improvement in DPT3 coverage and 2 % lower DPT-dropout rates at the subnational level [b= 0.22, p<0.05 and b=-0.21, p<0.01 respectively] (Table 4).

Table 4: The association between PHC service delivery subdomains and child health outputs at the subnational level

**Sub-domain**			**DPT3**		**DPT_ dropout**		**Pneumonia care seeking**		**ORS coverage**	
			Bivariate	Multivariable	Bivariate	Multivariable	Bivariate	Multivariable	Bivariate	Multivariable
**Capacit**	Facility Infrastructure	β	0.54**	0.45*	-0.37**	-0.30*	0.18	0.23	-0.02	0.01
		SE	0.15	0.18	0.12	0.14	0.18	0.22	0.12	0.15
	Supervision in the past 6 months	β	-0.04	0.08	0.03	-0.07	0.23*	0.34**	0.08	0.08
		SE	0.09	0.10	0.07	0.08	0.11	0.12	0.07	0.08
	Supervisor Feedback	β	-0.03	0.05	0.01	-0.05	0.22*	0.28**	0.10	0.09
		SE	0.09	0.09	0.07	0.07	0.10	0.11	0.07	0.07

For peer review only - http://bmjopen.bmj.com/site/about/guidelines.xhtml

	Outreach Services	β	-0.07	-0.02	0.06	0.01	-0.04	-0.05	0.05	0.04
		SE	0.06	0.06	0.04	0.05	0.07	0.07	0.05	0.05
	Not charging PHC fees	β	-0.14*	-0.17**	0.07	0.09	0.09	0.09	0.08	0.07
		SE	0.06	0.06	0.05	0.05	0.07	0.07	0.05	0.05
**Quality**	Waiting time < 60 minutes	β	-0.12	-0.11	0.08	0.07	0.06	0.06	-0.03	-0.03
		SE	0.07	0.07	0.06	0.05	0.08	0.08	0.06	0.06
	Provider Competence	β	0.09	0.03	-0.06	-0.02	0.13	0.14	-0.22	-0.19
		SE	0.19	0.18	0.14	0.14	0.21	0.21	0.14	0.14
	Provider Availability	β	-0.18	-0.17	0.13	0.12	0.11	0.11	-0.02	-0.06
		SE	0.10	0.10	0.08	0.08	0.12	0.12	0.08	0.08
	Person centeredness	β	-0.08	-0.10	0.06	0.08	0.04	0.03	-0.01	-0.02
		SE	0.06	0.06	0.05	0.05	0.07	0.07	0.05	0.05
	Safety	β	0.01	0.03	-0.03	-0.04	0.07	0.07	-0.09	-0.07
		SE	0.11	0.11	0.08	0.08	0.12	0.13	0.09	0.09
	Comprehensiveness	β	0.23*	0.22*	-0.22**	-0.21**	-0.10	-0.11	0.03	0.03
		SE	0.11	0.10	0.08	0.08	0.13	0.13	0.09	0.08

Standard errors in parentheses
* p<0.05, ** p<0.01
In multivariable models, results were adjusted for proportion of private facilities, hospitals, urban households, population in the 5th economic quintile and women with secondary education or more at the subnational level
Afghanistan was excluded from the analysis for subnational sample limitation

Discussion

Overall, the results of the descriptive analysis illustrate the heterogeneity in the capacity and quality of PHC service delivery within LMICs. The two-level analysis of maternal health outcomes yielded only a few statistically significant results at the p<0.05 level, however, none remained significant after adjusting for other covariates. This suggests that factors outside health facilities' control may play a greater role in determining mothers' care-seeking decisions for antenatal care and family planning, and in shaping perceptions of cost and geographic barriers to care. For example, individual- and household-level factors such as the mother's education and socioeconomic status are associated with the use of family planning and antenatal care services. Nonetheless, supply-side constraints at the health system level such as supply chain challenges may also contribute to stockouts of essential FP commodities (18). Analysis at levels more granular than the subnational area (the first administrative level) may yield further insight into the relationship of supply-side PHC characteristics and maternal health outcomes.

Meanwhile, the two-level analysis of child health outcomes revealed significant associations between supervision and care-seeking for pneumonia, as well as associations of several measures of capacity and quality with DPT3 immunization. Supervision in the last 6 months and supervisor feedback are considered measures of PHC capacity in the PHCPI framework, and it is hypothesized that measures of capacity operate through improved quality of health services. In the case of care-seeking for pneumonia, it is possible that facilities with greater capacity and implementation of supervision deliver care perceived to be of higher quality, thus increasing mothers' likelihood of seeking healthcare for their child at the facility. One review finds inconclusive but promising evidence that supportive supervision can improve quality of care, noting that supervision alone is insufficient; health workers require training and the availability of medicines, diagnostics and equipment to provide quality care (19). Patient perceptions of a facility's quality of care also influence utilization and care-seeking (20–26). There is also ample literature demonstrating the influence of various household and individual-level factors such as cultural beliefs, disease severity, geographic location, wealth, education, and socioeconomic status on care-seeking behavior (27,28)

For peer review only - http://bmjopen.bmj.com/site/about/guidelines.xhtml

Finally, our results found several associations with DPT3 immunization and dropout that generally align with existing literature (facility infrastructure and comprehensiveness) and in the case of user fees, counter to what might be expected. In line with our findings, prior research in Haiti found that both infrastructure quality and the quality-of-service delivery were associated with increased utilization of primary care services, including antenatal care and vaccination (29). Similarly, a study in rural India found that children in districts where more villages had a healthcare subcenter were more likely to receive at least one dose of DPT3, though no association with DPT3 dropout rates was observed (30). The results above also suggest that comprehensiveness, a measure of PHC quality, is also associated with better immunization outcomes. This is in line with prior studies documenting that children whose mothers were counseled during vaccination sessions were more likely to complete all three DPT doses (25).

One possible explanation behind the counterintuitive negative association between the absence of user fees and DPT3 immunization is that the estimated association may be confounded by other factors determining both user fee exemptions and immunization coverage. For example, subnational areas that are generally poorer and have greater barriers to care may be more likely to adopt policies exempting user fees, yet those characteristics may also result in lower immunization rates. A second explanation under the interpretation that the observed association is unconfounded may be related to potential unintended consequences of user fee removal. While the benefits of removing user fees are well documented in boosting health service utilization (31–33), some have also expressed concerns that the process of user fee removal must be designed carefully such that facilities are able to adapt. The elimination of fees and reduction of fees without a corresponding increase in resources from government sources could unintentionally lead to increased pressure on human resources and the availability of essential commodities, including vaccines. Further, increased demand for curative services without an increase in the health workforce could result in curative services "crowding out" preventative services such as vaccination. Ultimately, further research into any potentially paradoxical relationship between user fees and DPT3 vaccination is needed.

The study should also be interpreted in light of its limitations. As the study is observational and cross-sectional, the association presented should not be interpreted as causal effects. Using data aggregated at the subnational level also entails the potential for an ecological fallacy and requires caution in interpretation. In addition, while the sample was constructed such that the time between the SPA and DHS surveys in each country was less than 5 years, it is possible that changes in time-varying factors or other events in that interlude could influence the outcomes under study. Afghanistan's more limited sample size restricted to predominantly urban areas presents an additional limitation, therefore it was excluded from regression analysis to mitigate any potential confounding. Finally, though we control for selected characteristics at the subnational and individual levels, there is a potential for omitted variable bias through other factors that may influence the capacity, quality, and performance variables under study but are unmeasured in the SPA and DHS surveys. Ultimately, further research into the relationships between PHC capacity, quality and service delivery outcomes could adopt other methodologies to establish potential causal links. Taking advantage of repeated survey waves may provide one such strategy to track how these indicators may have changed over time and to what extent improvements in capacity and quality lead to better results.

Our findings have important policy implications. The heterogenicity observed in PHC service delivery within and between subnational areas highlights that there is not one solution that fits all. Countries and stakeholders seeking to strengthen their PHC systems could improve PHC monitoring at the subnational level. Disaggregation of national indicators by geographic regions and adoption of subnational scorecards can potentially unmask real bottlenecks that hinder the overall country's progress.

For peer review only - http://bmjopen.bmj.com/site/about/guidelines.xhtml

Furthermore, the low average scores of provider competence and availability across the studied countries point to the need for investing in ongoing training and professional development of PHC providers. In addition, a participatory and mixed-methods approach to monitoring is important to better understand the diverse challenges and access barriers across subnational areas and develop tailored approaches to resolve the problems identified.

Conclusion

Substantial heterogeneity and disparities were observed across the 12 studied LMICS, as well as between and within subnational areas in both the capacity and quality subdomains. Countries seeking to strengthen their PHC systems could improve PHC monitoring at the subnational level to better understand the diverse challenges across subnational areas and develop tailored approaches to resolve the problems identified. The associations observed between capacity, quality and performance also provide potential avenues for further research and contribute to the literature on the determinants of care-seeking behavior and immunization.

Ethics statement

This study was based on a secondary analysis of anonymized publicly available datasets. No ethics approval or patient consent was needed for the present analysis.

Data availability statement

The original datasets analyzed during the current study are available from the DHS program website, https://dhsprogram.com/data/available-datasets.cfm. The analytical dataset generated during the current study is available from the corresponding author on reasonable request

Contributors

MR and MVU were responsible for the conception of this study and for methods development. MR analyzed data. MR, JCG, CF prepared the first draft. All authors (MR, JCG, CF, SB, OB, MVU) reviewed results or reviewed and contributed to the final manuscript.

Funding

This research was supported, in whole or in part, by the Bill and Melinda Gates Foundation (Grant number INV000932), and it had no role in study design, data collection, data analysis, data interpretation or writing of the manuscript.

Competing interests

None declared.

References

1. World Health Organization, Organisation for Economic Co-operation and Development, International Bank for Reconstruction and Development. Delivering quality health services: a global imperative for

For peer review only - http://bmjopen.bmj.com/site/about/guidelines.xhtml

universal health coverage [Internet]. Geneva: World Health Organization; 2018. Available from: https://apps.who.int/iris/handle/10665/272465

2. Bitton A, Fifield J, Ratcliffe H, Karlage A, Wang H, Veillard JH, et al. Primary healthcare system performance in low-income and middle-income countries: a scoping review of the evidence from 2010 to 2017. BMJ Glob Health. 2019 Aug 16;4(Suppl 8):e001551.

3. Vital Signs Profiles | PHCPI [Internet]. [cited 2022 May 18]. Available from: https://improvingphc.org/vital-signs-profiles

4. Randhawa H. Report of the Expert Consultation on Primary Care Systems Profiles & Performance (PRIMASYS). :35.

5. Veillard J, Cowling K, Bitton A, Ratcliffe H, Kimball M, Barkley S, et al. Better Measurement for Performance Improvement in Low- and Middle-Income Countries: The Primary Health Care Performance Initiative (PHCPI) Experience of Conceptual Framework Development and Indicator Selection. Milbank Q. 2017 Dec;95(4):836–83.

6. Nikoloski Z, Albala S, Montero AM, Mossialos E. The impact of primary health care and specialist physician supply on amenable mortality in Mexico (2000-2015): Panel data analysis using system-Generalized Method of Moments. Soc Sci Med. 2021 Jun;278:113937.

7. Schneider MT, Chang AY, Crosby SW, Gloyd S, Harle AC, Lim S, et al. Trends and outcomes in primary health care expenditures in low-income and middle-income countries, 2000–2017. BMJ Glob Health. 2021 Aug;6(8):e005798.

8. Basu S, Berkowitz SA, Phillips RL, Bitton A, Landon BE, Phillips RS. Association of Primary Care Physician Supply With Population Mortality in the United States, 2005-2015. JAMA Intern Med. 2019 Apr 1;179(4):506–14.

9. Hone T, Rasella D, Barreto M, Atun R, Majeed A, Millett C. Large Reductions In Amenable Mortality Associated With Brazil’s Primary Care Expansion And Strong Health Governance. Health Aff (Millwood). 2017 Jan 1;36(1):149–58.

10. Russo LX, Scott A, Sivey P, Dias J. Primary care physicians and infant mortality: Evidence from Brazil. PLOS ONE. 2019 May 31;14(5):e0217614.

11. Ranabhat CL, Jakovljevic M, Dhimal M, Kim CB. Structural Factors Responsible for Universal Health Coverage in Low- and Middle-Income Countries: Results From 118 Countries. Frontiers in Public Health [Internet]. 2020 [cited 2022 Mar 14];7. Available from: https://www.frontiersin.org/article/10.3389/fpubh.2019.00414

12. Lozano R, Fullman N, Mumford JE, Knight M, Barthelemy CM, Abbafati C, et al. Measuring universal health coverage based on an index of effective coverage of health services in 204 countries and territories, 1990–2019: a systematic analysis for the Global Burden of Disease Study 2019. The Lancet. 2020 Oct 17;396(10258):1250–84.

13. Leslie HH, Malata A, Ndiaye Y, Kruk ME. Effective coverage of primary care services in eight high-mortality countries. BMJ Glob Health. 2017 Sep 4;2(3):e000424.

For peer review only

For peer review only - http://bmjopen.bmj.com/site/about/guidelines.xhtml

14. Nguhiu PK, Barasa EW, Chuma J. Determining the effective coverage of maternal and child health services in Kenya, using demographic and health survey data sets: tracking progress towards universal health coverage. Trop Med Int Health. 2017 Apr;22(4):442–53.

15. USAID. The DHS Program - Service Provision Assessments (SPA) [Internet]. Demographic Health Surveys. 2019 [cited 2020 Nov 28]. p. 1–2. Available from: https://dhsprogram.com/What-We-Do/Survey-Types/SPA.cfm

16. Croft TN, Marshall AM, Allen CK, Arnold F, Assaf S, Balian S. Guide to DHS statistics. Rockville: ICF. 2018;645.

[Dataset]17. The DHS Program - Available Datasets [Internet]. [cited 2022 Sep 19]. Available from: https://dhsprogram.com/data/available-datasets.cfm

18. Wulifan JK, Brenner S, Jahn A, De Allegri M. A scoping review on determinants of unmet need for family planning among women of reproductive age in low and middle income countries. BMC Womens Health. 2016 Jan 15;16:2.

19. Bailey C, Blake C, Schriver M, Cubaka VK, Thomas T, Martin Hilber A. A systematic review of supportive supervision as a strategy to improve primary healthcare services in Sub-Saharan Africa. International Journal of Gynecology & Obstetrics. 2016 Jan 1;132(1):117–25.

20. Rao KD, Sheffel A. Quality of clinical care and bypassing of primary health centers in India. Soc Sci Med. 2018 Jun;207:80–8.

21. Gauthier B, Wane W. Bypassing health providers: The quest for better price and quality of health care in Chad. Social Science & Medicine. 2011 Aug 1;73(4):540–9.

22. Kruk ME, Hermosilla S, Larson E, Mbaruku GM. Bypassing primary care clinics for childbirth: a cross-sectional study in the Pwani region, United Republic of Tanzania. Bull World Health Organ. 2014 Apr 1;92(4):246–53.

23. Alqossayir FM, Alkhowailed MS, Alammar AY, Alsaeed AA, Alamri YY, Rasheed Z. Factors associated with patients bypassing primary healthcare centres in Qassim Region, KSA. J Taibah Univ Med Sci. 2021 Dec;16(6):900–5.

24. Audo MO, Ferguson A, Njoroge PK. Quality of health care and its effects in the utilisation of maternal and child health services in Kenya. East Afr Med J. 2005 Nov;82(11):547–53.

25. Colvin CJ, Smith HJ, Swartz A, Ahs JW, de Heer J, Opiyo N, et al. Understanding careseeking for child illness in sub-Saharan Africa: a systematic review and conceptual framework based on qualitative research of household recognition and response to child diarrhoea, pneumonia and malaria. Soc Sci Med. 2013 Jun;86:66–78.

26. Bell SK, Delbanco T, Elmore JG, Fitzgerald PS, Fossa A, Harcourt K, et al. Frequency and Types of Patient-Reported Errors in Electronic Health Record Ambulatory Care Notes. JAMA Netw Open. 2020 Jun 1;3(6):e205867.

For peer review only - http://bmjopen.bmj.com/site/about/guidelines.xhtml

27. Geldsetzer P, Williams TC, Kirolos A, Mitchell S, Ratcliffe LA, Kohli-Lynch MK, et al. The Recognition of and Care Seeking Behaviour for Childhood Illness in Developing Countries: A Systematic Review. PLOS ONE. 2014 Apr 9;9(4):e93427.

28. Bruce N, Pope D, Arana B, Shiels C, Romero C, Klein R, et al. Determinants of Care Seeking for Children With Pneumonia and Diarrhea in Guatemala: Implications for Intervention Strategies. Am J Public Health. 2014 Apr;104(4):647–57.

29. Gage AD, Leslie HH, Bitton A, Jerome JG, Joseph JP, Thermidor R, et al. Does quality influence utilization of primary health care? Evidence from Haiti. Globalization and Health. 2018 Jun 20;14(1):59.

30. Ghosh A, Laxminarayan R. Demand- and supply-side determinants of diphtheria-pertussis-tetanus nonvaccination and dropout in rural India. Vaccine. 2017 Feb 15;35(7):1087–93.

31. Watson SI, Wroe EB, Dunbar EL, Mukherjee J, Squire SB, Nazimera L, et al. The impact of user fees on health services utilization and infectious disease diagnoses in Neno District, Malawi: a longitudinal, quasi-experimental study. BMC Health Services Research. 2016 Oct 20;16(1):595.

32. Nguyen HT, Zombré D, Ridde V, De Allegri M. The impact of reducing and eliminating user fees on facility-based delivery: a controlled interrupted time series in Burkina Faso. Health Policy and Planning. 2018 Oct 1;33(8):948–56.

33. Lagarde M, Barroy H, Palmer N. Assessing the effects of removing user fees in Zambia and Niger. J Health Serv Res Policy. 2012 Jan;17(1):30–6.

For peer review only

For peer review only - http://bmjopen.bmj.com/site/about/guidelines.xhtml

Supplementary Methods

Supplementary Table 1: Definition of capacity, quality and output indicators utilized in the analysis

Domain	Subdomain	Indicator	Definition	Data Source
**Capacity**	Facility Infrastructure	SRI for basic amenities and infection prevention	Service readiness index for basic amenities and infection prevention	SPA
	Recent Supervision	Supervision within the past 6 months	Proportion of providers within a facility with supervision in past 6 months	SPA
	Supervisor Feedback	Supervisor provided verbal or written feedback	Proportion of providers within a facility provided verbal or written supervision feedback	SPA
	Outreach Services	Facility provides outreach services	Facility provides sick child, growth monitoring or immunization outreach service	SPA
	Not charging PHC Fees	Facility does not charge PHC fees	Facility does not charge fees for PHC services (fees for a specific PHC service or a fixed fee that covers all services that a client receives)	SPA
**Quality**	Waiting Time < 60 minutes	Wait time less than one hour	Proportion of observed visits with wait times less than one hour	SPA
	Provider Availability	Proportion of visits over 10 minutes	The proportion of ANC, FP, LD and sick child visits that last for more than 10 minutes.	SPA
	Provider Competence	Antenatal care quality score based on WHO guidelines	Average quality score for observed ANC visits based on WHO ANC guidelines	SPA
		Family planning quality score based on WHO guidelines	Average quality score for observed family visits based on WHO family planning guidelines	
		Sick child quality score based on IMCI guidelines	Average quality score for observed sick child visits based on WHO Integrated Management of Childhood Illness (IMCI) guidelines	
	Person Centeredness	Proportion of caregivers who were told the sick child diagnosis	Proportion of observed sick child visits where the health worker told the child's caretaker what illness(es) the child has	SPA
	Safety	Proportion of rooms with all infection control items	Proportion of rooms (FP, sick child, ANC and NCD) where all infection control tracer items are present	SPA
		Adequate waste disposal	Average score (out of 3) on adherence to standards for disposing of medical and hazardous waste, sharps, and having guidelines for waste disposal in place.	
	Comprehensiveness	Proportion of 3 NCD services offering diagnosis and treatment (diabetes, CRD, CVD)	Proportion of non-combinable disease services provided and for which guidelines are available (diabetes, chronic respiratory disease, and chronic cardiovascular disease) across all facilities	SPA
		Proportion of 3 primary ID services provided (STI, TB, HIV)	Proportion of infectious diseases services provided and for which guidelines are available for sexually transmitted infections, tuberculosis, and HIV across all facilities.	SPA
		Average availability of 5 tracer RMNCH services (SC, Vacc, FP, ANC, PMTCT)	Proportion of maternal and child health services provided and for which guidelines are available (sick child, vaccination, family planning, antenatal care, and prevention of mother-to-child transmission of HIV) across all facilities.	SPA
**Outputs**	Maternal	Antenatal care coverage - at least four visits (%)	Antenatal care coverage (4+) visits is the percent of women with a live birth who received antenatal care (ANC) 4 or more times.	DHS
		Demand for family planning satisfied with modern methods (%)	Proportion of married or in-union women of reproductive age (aged 15-49 years) who are married or in a union and have their need for family planning satisfied with modern methods.	DHS
		Perceived barriers due to treatment costs	Percentage of women who report specific problems in accessing health care when they are sick due to issues related to getting money for treatment	DHS
		Perceived barriers due to distance	Percentage of women who report specific problems in accessing health care when they are sick due to distance that they have to travel for treatment	DHS
	Child	Coverage of DTP3 immunization	Diphtheria-tetanus-pertussis (DTP) coverage measures the percent of one-year-olds who have received three doses of the combined diphtheria, tetanus toxoid and pertussis vaccine in a given year.	DHS
		DPT Dropout rate	Percent of children who do not receive three doses of DTP after receiving an initial dose. Calculated as (DPT1-DPT3/DPT1)	DHS
		Children aged < 5 years with pneumonia symptoms taken to a healthcare provider (%)	Percentage of children under 5 years of age with suspected pneumonia (cough and difficulty breathing NOT due to a problem in the chest and a blocked nose) in the two weeks preceding the survey taken to an appropriate health facility or provider.	DHS
		Children aged < 5 years with diarrhea receiving oral rehydration salts	The percent of children with diarrhea, a leading cause of death in children under five, who received appropriate treatment with oral rehydration solution.	DHS

Supplementary Figure 1: Linkage of SPA and DHS datasets

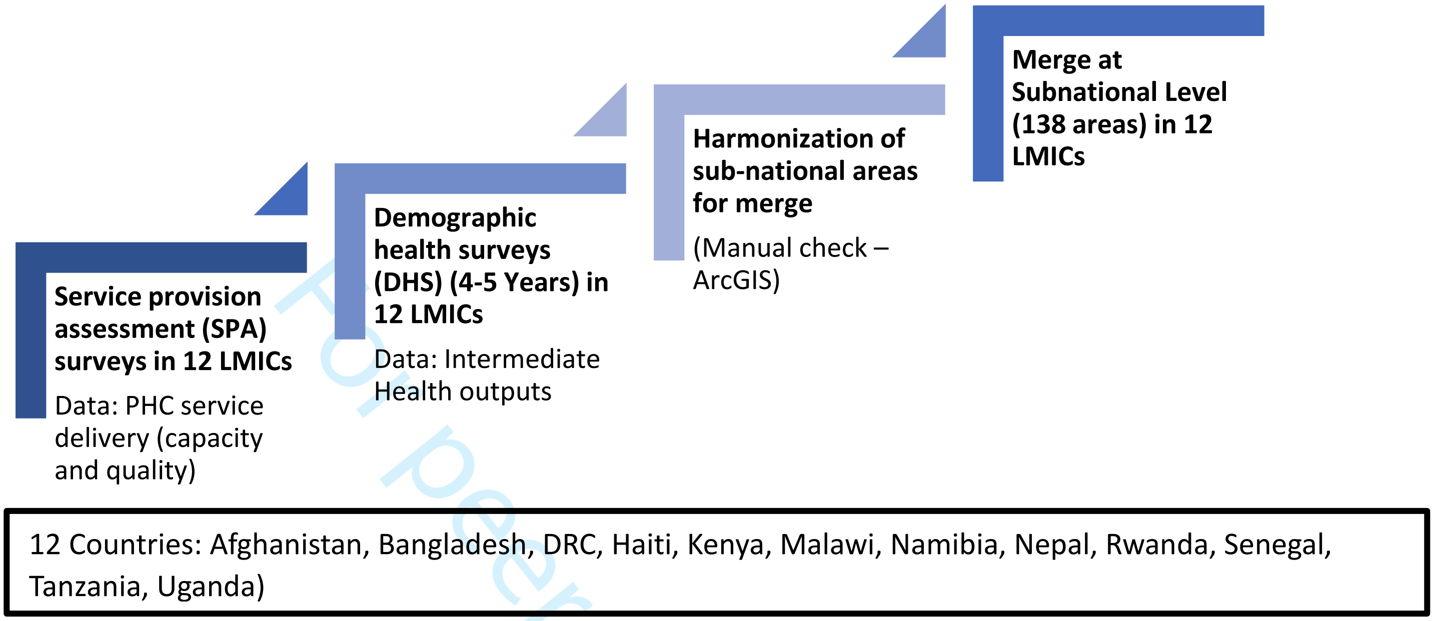

For peer review only

Supplementary Results

Supplementary Table 2: Capacity of PHC service delivery at the subnational level in the studied countries

Country	Division	Facility Infrastructure	Recent Supervision	Supervisor Feedback	Outreach Services	Not Charging PHC Fees	Average Capacity score	Range across capacity sub-domains*
Afghanistan	Balkh	83%	85%	63%	7%	56%	59%	78%
	Herat	85%	59%	29%	4%	71%	50%	81%
	Kabul	86%	63%	54%	31%	36%	54%	55%
	Kandahar	82%	99%	73%	29%	45%	66%	70%
	Kunduz	76%	91%	76%	0%	53%	59%	91%
	Nangarhar	75%	70%	63%	0%	45%	51%	75%
	Paktya	74%	70%	44%	25%	43%	51%	49%
Bangladesh	Barisal	49%	95%	93%	14%	67%	64%	81%
	Chittagong	51%	91%	85%	20%	80%	65%	71%
	Dhaka	52%	92%	89%	26%	54%	63%	66%
	Khulna	54%	97%	92%	21%	54%	64%	76%
	Mymensingh	46%	99%	96%	14%	45%	60%	85%
	Rajshahi	55%	96%	94%	19%	81%	69%	77%
	Rangpur	51%	97%	96%	14%	76%	67%	83%
	Sylhet	48%	89%	87%	18%	78%	64%	71%
DRC	Bas-uele	37%	68%	66%	91%	73%	67%	54%
	Equateur	42%	80%	71%	95%	24%	62%	71%
	Haut-katanga	45%	73%	63%	73%	46%	60%	28%
	Haut-lomami	53%	57%	49%	29%	56%	49%	28%
	Haut-uele	42%	75%	68%	81%	44%	62%	39%
	Ituri	51%	79%	74%	82%	30%	63%	52%
	Kasai	43%	72%	63%	25%	34%	47%	47%
	Kasai-central	49%	86%	86%	84%	40%	69%	46%
	Kasai-oriental	43%	83%	79%	74%	59%	67%	40%
	Kinshasa	60%	64%	56%	33%	66%	56%	33%

	Kongo central	55%	58%	54%	40%	58%	53%	18%
	Kwango	48%	91%	86%	95%	16%	67%	79%
	Kwilu	48%	71%	64%	90%	20%	58%	70%
	Lomami	47%	91%	91%	86%	37%	71%	54%
	Lualaba	54%	77%	76%	54%	65%	65%	23%
	Mai-ndombe	47%	85%	77%	92%	36%	67%	56%
	Maniema	57%	89%	85%	65%	65%	72%	32%
	Mongala	40%	87%	65%	78%	39%	62%	48%
	Nord-kivu	43%	91%	84%	94%	0%	63%	94%
	Nord-ubangi	55%	83%	82%	64%	34%	64%	49%
	Sankuru	36%	64%	56%	83%	81%	64%	47%
	Sud-kivu	49%	88%	85%	86%	0%	62%	88%
	Sud-ubangi	60%	75%	71%	69%	16%	58%	59%
	Tanganyka	47%	72%	71%	75%	64%	66%	28%
	Tshopo	41%	71%	67%	67%	52%	60%	30%
	Tshuapa	44%	84%	81%	94%	36%	68%	58%
**Haiti**	Artibonite	60%	78%	70%	67%	62%	67%	18%
	Centre	58%	90%	79%	70%	72%	74%	32%
	Grand'anse	66%	84%	76%	83%	78%	77%	18%
	Nippes	67%	64%	52%	29%	58%	54%	38%
	Nord	68%	73%	56%	42%	68%	61%	31%
	Nord-est	61%	66%	60%	45%	64%	59%	21%
	Nord-ouest	63%	58%	56%	4%	84%	53%	80%
	Ouest	59%	72%	63%	13%	76%	57%	63%
	Sud	56%	75%	49%	20%	98%	60%	78%
	Sud-est	63%	83%	75%	22%	68%	62%	61%
**Kenya**	Central	69%	76%	66%	64%	46%	64%	30%
	Coast	69%	71%	65%	53%	70%	66%	18%
	Eastern	71%	70%	60%	46%	64%	62%	25%
	Nairobi	61%	81%	72%	71%	72%	71%	20%
	North eastern	61%	83%	70%	42%	63%	64%	41%
	Nyanza	62%	69%	52%	31%	92%	61%	61%
	Rift valley	72%	67%	60%	14%	66%	56%	58%
	Western	61%	73%	62%	20%	63%	56%	53%

**Malawi**	Central region	68%	73%	64%	67%	67%	68%	9%
	Northern region	65%	75%	67%	78%	82%	73%	17%
	Southern region	69%	74%	65%	64%	72%	69%	10%
**Namibia**	Caprivi	58%	77%	49%	3%	97%	57%	94%
	Erongo	73%	63%	58%	18%	95%	61%	77%
	Hardap	78%	78%	69%	17%	83%	65%	66%
	Karas	72%	73%	68%	15%	92%	64%	77%
	Kavango	61%	71%	53%	21%	97%	60%	76%
	Khomas	74%	56%	50%	0%	75%	51%	75%
	Kunene	63%	69%	56%	14%	93%	59%	79%
	Ohangwena	66%	83%	68%	21%	100%	68%	79%
	Omaheke	75%	84%	82%	25%	100%	73%	75%
	Omusati	72%	79%	68%	42%	100%	72%	58%
	Oshana	68%	78%	66%	43%	100%	71%	57%
	Oshikoto	70%	70%	57%	18%	95%	62%	77%
	Otjozondjupa	74%	52%	47%	20%	97%	58%	77%
**Nepal**	Central	54%	71%	61%	73%	92%	70%	38%
	Eastern	54%	66%	60%	86%	93%	72%	39%
	Far-western	52%	76%	67%	88%	90%	75%	38%
	Mid-western	53%	71%	64%	88%	90%	73%	37%
	Western	54%	74%	69%	87%	94%	76%	40%
**Rowanda**	East	56%	91%	79%	81%	97%	81%	41%
	Kigali city	70%	56%	48%	69%	92%	67%	44%
	North	60%	84%	75%	80%	100%	80%	40%
	South	64%	91%	86%	87%	93%	84%	29%
	West	62%	90%	83%	77%	98%	82%	36%
**Senegal**	Dakar	79%	64%	62%	22%	67%	59%	57%
	Diourbel	83%	81%	80%	64%	60%	74%	23%
	Fatick	66%	91%	89%	37%	57%	68%	54%
	Kaffrine	47%	86%	86%	45%	72%	67%	41%
	Kaolack	51%	68%	67%	16%	66%	54%	52%
	Kedougou	45%	78%	78%	58%	66%	65%	33%
	Kolda	45%	86%	81%	61%	69%	69%	41%

	Louga	61%	58%	57%	53%	65%	59%	12%
	Matam	63%	73%	70%	58%	66%	66%	15%
	Saint-louis	62%	74%	74%	56%	60%	65%	18%
	Sedhiou	68%	100%	98%	51%	61%	75%	49%
	Tambacounda	61%	69%	66%	77%	66%	68%	16%
	This	61%	69%	68%	46%	67%	62%	23%
	Ziguinchor	70%	87%	86%	35%	64%	68%	52%
**Tanzania**	Arusha	62%	77%	68%	19%	14%	48%	63%
	Dar es salaam	49%	68%	67%	47%	11%	48%	57%
	Dodoma	73%	59%	55%	15%	52%	51%	58%
	Geita	54%	57%	54%	57%	17%	48%	40%
	Iringa	57%	58%	57%	58%	23%	51%	35%
	Kagera	55%	47%	41%	63%	15%	44%	48%
	Kaskazini pemba	59%	85%	78%	76%	29%	65%	56%
	Kaskazini unguja	62%	78%	74%	50%	86%	70%	36%
	Katavi	66%	83%	81%	40%	84%	71%	44%
	Kigoma	49%	80%	70%	82%	14%	59%	68%
	Kilimanjaro	52%	79%	66%	26%	25%	50%	54%
	Kusini pemba	61%	75%	66%	34%	41%	55%	41%
	Kusini unguja	61%	68%	64%	59%	86%	68%	27%
	Lindi	64%	78%	77%	15%	81%	63%	66%
	Manyara	52%	86%	77%	38%	26%	56%	60%
	Mara	53%	86%	78%	37%	35%	58%	51%
	Mbeya	54%	74%	70%	47%	22%	53%	52%
	Mjini magharibi	55%	59%	56%	59%	22%	50%	37%
	Morogoro	57%	65%	62%	56%	25%	53%	40%
	Mtwara	66%	45%	43%	16%	48%	44%	50%
	Mwanza	60%	48%	40%	73%	21%	49%	52%
	Njombe	51%	66%	56%	40%	13%	45%	53%
	Pwani	53%	54%	53%	51%	23%	47%	31%
	Rukwa	49%	44%	40%	54%	4%	38%	50%
	Ruvuma	51%	45%	36%	67%	28%	45%	39%
	Shinyanga	49%	95%	92%	70%	31%	67%	64%

	Simiyu	53%	79%	72%	64%	12%	56%	67%
	Singida	54%	90%	89%	75%	32%	68%	58%
	Tabora	51%	75%	74%	66%	15%	56%	60%
	Tanga	50%	90%	90%	38%	18%	57%	72%
**Uganda**	Central 1	52%	91%	78%	83%	100%	81%	48%
	Central 2	44%	91%	70%	77%	97%	76%	53%
	East central	55%	82%	69%	85%	100%	78%	45%
	Eastern	70%	60%	49%	57%	93%	66%	44%
	Kampala	54%	85%	68%	85%	100%	78%	46%
	North	45%	79%	34%	84%	100%	68%	66%
	Southwest	55%	82%	59%	82%	100%	76%	45%
	West-nile	55%	69%	57%	67%	100%	70%	45%
	Western	64%	93%	72%	94%	100%	85%	36%

For peer review only

Supplementary Table 3: Quality of PHC service delivery at the subnational level in the studied countries

Country	Division	Waiting Time	Provider Availability	Provider Competence	Person Centeredness	Safety	Comprehensive-ness	Average Quality score	Range across quality Subdomains *
Afghanistan	Balkh	89%	35%	28%	75%	28%	65%	53%	61%
	Herat	84%	34%	34%	50%	33%	52%	48%	51%
	Kabul	91%	20%	24%	34%	52%	67%	48%	71%
	Kandahar	100%	34%	19%	1%	55%	75%	47%	99%
	Kunduz	87%	42%	38%	89%	6%	69%	55%	83%
	Nangarhar	97%	48%	34%	45%	17%	78%	53%	80%
	Paktya	94%	54%	30%	22%	23%	73%	49%	72%
Bangladesh	Barisal	-	-	-	-	39%	67%	53%	28%
	Chittagong	-	-	-	-	39%	64%	52%	25%
	Dhaka	-	-	-	-	40%	61%	50%	21%
	Khulna	-	-	-	-	39%	64%	52%	25%
	Mymensingh	-	-	-	-	31%	65%	48%	34%
	Rajshahi	-	-	-	-	44%	68%	56%	24%
	Rangpur	-	-	-	-	45%	50%	47%	5%
	Sylhet	-	-	-	-	33%	68%	51%	35%
DRC	Bas-uele	74%	68%	39%	45%	35%	70%	55%	39%
	Equateur	90%	86%	36%	46%	28%	74%	60%	62%
	Haut-katanga	82%	89%	45%	40%	22%	86%	61%	67%
	Haut-lomami	78%	65%	43%	22%	45%	87%	57%	65%
	Haut-uele	63%	64%	34%	20%	26%	70%	46%	50%
	Ituri	89%	75%	39%	24%	39%	56%	54%	65%
	Kasai	95%	82%	30%	8%	19%	53%	48%	87%
	Kasai-central	73%	78%	39%	45%	30%	68%	56%	48%

	Kasai-oriental	69%	72%	38%	39%	28%	79%	54%	51%
	Kinshasa	44%	69%	48%	22%	29%	79%	48%	57%
	Kongo central	83%	88%	44%	33%	34%	88%	62%	55%
	Kwango	87%	87%	40%	11%	28%	71%	54%	76%
	Kwilu	65%	67%	37%	12%	37%	87%	51%	75%
	Lomami	70%	66%	37%	28%	36%	91%	55%	63%
	Lualaba	65%	63%	47%	40%	34%	91%	56%	57%
	Mai-ndombe	75%	86%	38%	23%	27%	78%	55%	63%
	Maniema	78%	71%	37%	46%	42%	94%	61%	57%
	Mongala	85%	89%	36%	50%	21%	71%	59%	68%
	Nord-kivu	65%	78%	42%	47%	30%	58%	54%	48%
	Nord-ubangi	77%	76%	48%	9%	49%	79%	56%	70%
	Sankuru	88%	87%	35%	30%	27%	49%	53%	61%
	Sud-kivu	75%	65%	40%	33%	36%	81%	55%	48%
	Sud-ubangi	71%	82%	44%	49%	43%	68%	60%	39%
	Tanganyka	80%	87%	33%	31%	42%	52%	54%	56%
	Tshopo	81%	88%	37%	20%	27%	71%	54%	68%
	Tshuapa	82%	88%	38%	41%	30%	69%	58%	58%
**Haiti**	Artibonite	50%	62%	36%	15%	51%	79%	49%	64%
	Centre	66%	70%	33%	7%	56%	88%	53%	81%
	Grand'anse	48%	71%	37%	8%	61%	85%	51%	77%
	Nippes	42%	59%	34%	18%	45%	75%	45%	57%
	Nord	52%	67%	36%	13%	55%	83%	51%	70%
	Nord-est	46%	68%	39%	10%	53%	77%	49%	67%
	Nord-ouest	64%	67%	49%	34%	76%	70%	60%	42%
	Ouest	70%	57%	44%	48%	51%	78%	58%	34%
	Sud	93%	54%	37%	44%	45%	64%	56%	56%
	Sud-est	53%	72%	58%	58%	60%	89%	65%	36%

Kenya	Central	30%	60%	36%	9%	55%	81%	45%	72%
	Coast	49%	63%	35%	14%	70%	90%	54%	76%
	Eastern	54%	63%	35%	15%	58%	83%	51%	68%
	Nairobi	53%	72%	32%	20%	56%	83%	52%	63%
	North eastern	73%	59%	44%	39%	66%	89%	62%	50%
	Nyanza	84%	34%	46%	71%	78%	72%	64%	50%
	Rift valley	65%	77%	50%	70%	71%	71%	67%	27%
	Western	75%	75%	51%	68%	67%	76%	69%	25%
Malawi	Central region	49%	43%	38%	51%	50%	75%	51%	37%
	Northern region	46%	42%	37%	41%	46%	77%	48%	40%
	Southern region	49%	39%	37%	43%	51%	72%	48%	35%
Namibia	Caprivi	30%	70%	52%	29%	81%	93%	59%	64%
	Erongo	59%	49%	52%	76%	65%	77%	63%	28%
	Hardap	49%	69%	51%	45%	86%	84%	64%	41%
	Karas	65%	59%	48%	45%	78%	87%	64%	42%
	Kavango	34%	48%	49%	30%	75%	94%	55%	64%
	Khomas	49%	69%	51%	84%	86%	55%	66%	37%
	Kunene	73%	76%	59%	79%	78%	86%	75%	27%
	Ohangwena	8%	55%	59%	63%	70%	93%	58%	85%
	Omaheke	58%	79%	67%	79%	91%	90%	77%	33%
	Omusati	39%	59%	49%	35%	69%	92%	57%	57%
	Oshana	25%	62%	60%	65%	59%	84%	59%	59%
	Oshikoto	34%	74%	64%	53%	54%	95%	62%	61%
	Otjozondjupa	46%	48%	47%	26%	61%	79%	51%	53%
Nepal	Central	96%	31%	26%	27%	42%	76%	50%	70%
	Eastern	90%	47%	31%	40%	44%	76%	55%	59%
	Far-western	94%	58%	34%	37%	42%	79%	57%	60%

	Mid-western	91%	45%	32%	27%	49%	74%	53%	64%
	Western	96%	41%	33%	37%	49%	75%	55%	63%
Rowanda	East	31%	41%	40%	13%	21%	79%	37%	66%
	Kigali city	52%	51%	45%	51%	31%	57%	48%	26%
	North	17%	34%	41%	27%	24%	76%	37%	59%
	South	43%	33%	35%	19%	32%	83%	41%	64%
	West	42%	37%	44%	18%	24%	78%	40%	60%
Senegal	Dakar	71%	63%	42%	24%	67%	70%	56%	47%
	Diourbel	99%	71%	42%	10%	81%	77%	63%	89%
	Fatick	94%	70%	42%	10%	72%	58%	58%	84%
	Kaffrine	71%	61%	47%	4%	53%	52%	48%	67%
	Kaolack	78%	69%	48%	29%	58%	53%	56%	49%
	Kedougou	86%	77%	49%	8%	45%	56%	53%	78%
	Kolda	76%	71%	44%	6%	49%	51%	50%	70%
	Louga	63%	49%	34%	1%	59%	74%	47%	73%
	Matam	72%	24%	28%	1%	75%	74%	46%	74%
	Saint-louis	43%	49%	33%	2%	68%	73%	45%	71%
	Sedhiou	91%	72%	38%	1%	79%	64%	58%	90%
	Tambacounda	62%	59%	32%	0%	69%	81%	50%	81%
	This	74%	60%	43%	20%	61%	64%	54%	54%
	Ziguinchor	97%	74%	37%	12%	74%	66%	60%	85%
Tanzania	Arusha	74%	76%	45%	44%	63%	59%	60%	32%
	Dar es salaam	59%	82%	42%	68%	35%	72%	60%	47%
	Dodoma	76%	76%	40%	44%	52%	56%	57%	36%
	Geita	71%	87%	35%	13%	26%	71%	51%	74%
	Iringa	42%	60%	44%	78%	30%	75%	54%	48%
	Kagera	76%	70%	45%	52%	37%	74%	59%	39%

	Kaskazini pemba	28%	72%	46%	77%	35%	76%	56%	49%
	Kaskazini unguja	71%	74%	39%	19%	27%	76%	51%	57%
	Katavi	22%	81%	47%	65%	66%	87%	61%	65%
	Kigoma	46%	74%	35%	67%	31%	83%	56%	52%
	Kilimanjaro	28%	56%	40%	41%	24%	83%	45%	59%
	Kusini pemba	84%	82%	35%	33%	54%	68%	59%	51%
	Kusini unguja	67%	82%	38%	23%	25%	74%	51%	59%
	Lindi	58%	77%	44%	67%	58%	89%	65%	45%
	Manyara	66%	61%	34%	57%	42%	63%	54%	32%
	Mara	54%	79%	48%	27%	47%	57%	52%	52%
	Mbeya	69%	74%	33%	24%	36%	61%	49%	50%
	Mjini magharibi	68%	59%	35%	46%	50%	78%	56%	43%
	Morogoro	61%	50%	36%	16%	61%	70%	49%	54%
	Mtwara	72%	57%	33%	50%	41%	66%	53%	39%
	Mwanza	41%	68%	38%	54%	44%	71%	53%	33%
	Njombe	55%	65%	43%	78%	36%	68%	58%	42%
	Pwani	41%	71%	47%	44%	36%	70%	52%	35%
	Rukwa	81%	76%	45%	27%	40%	60%	55%	54%
	Ruvuma	62%	43%	29%	19%	36%	75%	44%	56%
	Shinyanga	50%	76%	36%	48%	39%	65%	52%	40%
	Simiyu	64%	69%	37%	38%	34%	58%	50%	35%
	Singida	50%	74%	48%	81%	34%	70%	60%	47%
	Tabora	39%	64%	39%	30%	44%	63%	46%	34%
	Tanga	50%	70%	52%	49%	34%	80%	56%	46%
**Uganda**	Central 1	64%	46%	44%	20%	27%	81%	47%	61%
	Central 2	69%	31%	54%	64%	23%	65%	51%	46%

	East central	53%	46%	56%	71%	27%	70%	54%	44%
	Eastern	50%	51%	56%	72%	37%	86%	59%	49%
	Kampala	47%	36%	60%	82%	23%	76%	54%	59%
	North	22%	21%	39%	36%	17%	61%	33%	44%
	Southwest	33%	51%	40%	39%	36%	72%	45%	39%
	West-nile	66%	49%	48%	65%	22%	64%	52%	44%
	Western	34%	42%	43%	39%	24%	73%	43%	49%

For peer review only

For peer review only

The RECORD statement – checklist of items, extended from the STROBE statement, that should be reported in observational studies using routinely collected health data.

	Item No.	STROBE items	Location in manuscript where items are reported	RECORD items	Location in manuscript where items are reported
**Title and abstract**					
	1	(a) Indicate the study's design with a commonly used term in the title or the abstract (b) Provide in the abstract an informative and balanced summary of what was done and what was found	(a) line 24, page 2<br><br>(b) lines 21-45, page 2	RECORD 1.1: The type of data used should be specified in the title or abstract. When possible, the name of the databases used should be included.<br><br>RECORD 1.2: If applicable, the geographic region and timeframe within which the study took place should be reported in the title or abstract.<br><br>RECORD 1.3: If linkage between databases was conducted for the study, this should be clearly stated in the title or abstract.	1.1: lines 24-26, page 2<br><br>1.2: lines 26-28, page 2<br><br>1.3: lines 24-25, page 2
**Introduction**					
Background rationale	2	Explain the scientific background and rationale for the investigation being reported	Lines 63-89, page 4		
Objectives	3	State specific objectives, including any prespecified hypotheses	Lines 90-94, page 4		
**Methods**					
Study Design	4	Present key elements of study design early in the paper	Lines 97-119, page 4		
Setting	5	Describe the setting, locations, and relevant dates, including periods of recruitment, exposure, follow-up, and data collection	Lines 99-119, page 4-5<br>Table 1-page 5		

For peer review only

Participants	6	*(a) Cohort study* - Give the eligibility criteria, and the sources and methods of selection of participants. Describe methods of follow-up<br>*Case-control study* - Give the eligibility criteria, and the sources and methods of case ascertainment and control selection. Give the rationale for the choice of cases and controls<br>*Cross-sectional study* - Give the eligibility criteria, and the sources and methods of selection of participants<br><br>*(b) Cohort study* - For matched studies, give matching criteria and number of exposed and unexposed<br>*Case-control study* - For matched studies, give matching criteria and the number of controls per case	Lines 99-119, page 4-5<br>Table 1, page 5	RECORD 6.1: The methods of study population selection (such as codes or algorithms used to identify subjects) should be listed in detail. If this is not possible, an explanation should be provided.<br><br>RECORD 6.2: Any validation studies of the codes or algorithms used to select the population should be referenced. If validation was conducted for this study and not published elsewhere, detailed methods and results should be provided.<br><br>RECORD 6.3: If the study involved linkage of databases, consider use of a flow diagram or other graphical display to demonstrate the data linkage process, including the number of individuals with linked data at each stage.	6.1: NA- secondary analysis of publicly available anonymized data was used<br>6.2: NA- secondary analysis of publicly available anonymized data was used<br><br>6.3: Figure S1 Supplementary methods
Variables	7	Clearly define all outcomes, exposures, predictors, potential confounders, and effect modifiers. Give diagnostic criteria, if applicable.	Lines 125-147, Page 6<br><br>Table S1, supplementary methods	RECORD 7.1: A complete list of codes and algorithms used to classify exposures, outcomes, confounders, and effect modifiers should be provided. If these cannot be reported, an explanation should be provided.	Lines 125-145, Page 5<br><br>Table S1, supplementary methods
Data sources/ measurement	8	For each variable of interest, give sources of data and details of methods of assessment (measurement).<br>Describe comparability of assessment methods if there is more than one group	Lines 118-141, Page 5<br><br>Table S2, supplementary file 1		

For peer review only

Bias	9	Describe any efforts to address potential sources of bias	Lines 159-170, page 7		
Study size	10	Explain how the study size was arrived at	Secondary analysis of public data was conducted. The selected sample was explained<br>Lines 99-103, page 4-5<br>Table 1, page 5		
Quantitative variables	11	Explain how quantitative variables were handled in the analyses. If applicable, describe which groupings were chosen, and why	Lines 125-147, page 6		
Statistical methods	12	(a) Describe all statistical methods, including those used to control for confounding<br>(b) Describe any methods used to examine subgroups and interactions<br>(c) Explain how missing data were addressed<br>(d) *Cohort study* - If applicable, explain how loss to follow-up was addressed<br>*Case-control study* - If applicable, explain how matching of cases and controls was addressed<br>*Cross-sectional study* - If applicable, describe analytical methods taking account of sampling strategy<br>(e) Describe any sensitivity analyses	a) lines 149-170, page 7<br><br>b) NA<br><br>c)lines 153, 158 - page 7<br><br>d) NA<br><br>e) NA		

For peer review only

Data access and cleaning methods		..		RECORD 12.1: Authors should describe the extent to which the investigators had access to the database population used to create the study population.<br><br>RECORD 12.2: Authors should provide information on the data cleaning methods used in the study.	12.1: Data is publicly available so section 12.1 does not apply<br><br>12.2: NA as access to already cleaned data was provided through DHS website
Linkage		..		RECORD 12.3: State whether the study included person-level, institutional-level, or other data linkage across two or more databases. The methods of linkage and methods of linkage quality evaluation should be provided.	12.3: lines 149-158, pages 7
**Results**					
Participants	13	(a) Report the numbers of individuals at each stage of the study (*e.g.*, numbers potentially eligible, examined for eligibility, confirmed eligible, included in the study, completing follow-up, and analysed)<br>(b) Give reasons for non-participation at each stage.<br>(c) Consider use of a flow diagram	Lines 168-169, page 6	RECORD 13.1: Describe in detail the selection of the persons included in the study (*i.e.,* study population selection) including filtering based on data quality, data availability and linkage. The selection of included persons can be described in the text and/or by means of the study flow diagram.	Table 1, page 4-6 and Supplementary Figure 21 in Supplementary Methods
Descriptive data	14	(a) Give characteristics of study participants (*e.g.*, demographic, clinical, social) and information	Demographic of patients is not applicable; characteristics of		

For peer review only

		on exposures and potential confounders<br>(b) Indicate the number of participants with missing data for each variable of interest<br>(c) *Cohort study* - summarise follow-up time (*e.g.*, average and total amount)	included surveys are described in Table 1 -page 5		
Outcome data	15	*Cohort study* - Report numbers of outcome events or summary measures over time<br>*Case-control study* - Report numbers in each exposure category, or summary measures of exposure<br>*Cross-sectional study* - Report numbers of outcome events or summary measures	Table 2 – page 8<br>Table 3- page 9-10<br>Table 4- page 10-11		
Main results	16	(a) Give unadjusted estimates and, if applicable, confounder-adjusted estimates and their precision (e.g., 95% confidence interval). Make clear which confounders were adjusted for and why they were included<br>(b) Report category boundaries when continuous variables were categorized<br>(c) If relevant, consider translating estimates of relative risk into absolute risk for a meaningful time period	Table 3, page 9-10<br>&<br>Table 4, page 10-11<br>standard errors were included and confounders are added as footnotes<br>NA<br>NA		
Other analyses	17	Report other analyses done—e.g., analyses of subgroups and	NA		

For peer review only

		interactions, and sensitivity analyses			
**Discussion**					
Key results	18	Summarise key results with reference to study objectives	Lines 273-307, page 11-12		
Limitations	19	Discuss limitations of the study, taking into account sources of potential bias or imprecision. Discuss both direction and magnitude of any potential bias	Lines 323-337, page 12	RECORD 19.1: Discuss the implications of using data that were not created or collected to answer the specific research question(s). Include discussion of misclassification bias, unmeasured confounding, missing data, and changing eligibility over time, as they pertain to the study being reported.	Lines 323-337, page 12
Interpretation	20	Give a cautious overall interpretation of results considering objectives, limitations, multiplicity of analyses, results from similar studies, and other relevant evidence	Lines 273-322, page 11-12		
Generalisability	21	Discuss the generalisability (external validity) of the study results	NA – this is a multi-country study, all countries with available SPA survey were considered		
**Other Information**					
Funding	22	Give the source of funding and the role of the funders for the present study and, if applicable, for the original study on which the present article is based	Lines 364-366, page 13		
Accessibility of protocol, raw data, and programming code		..		RECORD 22.1: Authors should provide information on how to access any supplemental information such as the study protocol, raw data, or programming code.	Lines 358-360, page 13

For peer review only

*Reference: Benchimol EI, Smeeth L, Guttmann A, Harron K, Moher D, Petersen I, Sørensen HT, von Elm E, Langan SM, the RECORD Working Committee. The REporting of studies Conducted using Observational Routinely-collected health Data (RECORD) Statement. *PLoS Medicine* 2015; in press.

*Checklist is protected under Creative Commons Attribution (CC BY) license.

For peer review only
